# Exploratory use of intraprocedural transesophageal echocardiography to guide implantation of the leadless pacemaker

**DOI:** 10.1016/j.hroo.2022.10.005

**Published:** 2022-10-18

**Authors:** Bashaer Gheyath, Roshni Vijay Khatiwala, Shaomin Chen, Zhifan Fu, Neil Beri, Carter English, Heejung Bang, Uma Srivatsa, Nayereh Pezeshkian, Kwame Atsina, Dali Fan

**Affiliations:** ∗ Division of Cardiovascular Medicine, Department of Internal Medicine, University of California Davis, Sacramento, California; † Department of Cardiology and Institute of Vascular Medicine, Peking University Third Hospital, Beijing, China; ‡ Department of Geriartics, Peking University First Hospital, Beijing, China; § Division of Biostatistics, Department of Public Health Sciences, University of California Davis, Sacramento, California

**Keywords:** Intraprocedural imaging, Leadless pacemaker, Nonfluoroscopic imaging, Septal pacing, Transesophageal echocardiography

## Abstract

**Background:**

Fluoroscopy is the standard tool for transvenous implantation of traditional and leadless pacemakers (LPs). LPs are used to avoid complications of conventional pacemakers, but there still is a 6.5% risk of major complications. Mid–right ventricular (RV) septal device implantation is suggested to decrease the risk, but helpful cardiac landmarks cannot be visualized under fluoroscopy. Transesophageal echocardiography (TEE) is an alternative intraprocedural imaging method.

**Objective:**

The purpose of this study was to explore the spatial relationship of the LP to cardiac landmarks via TEE and their correlations with electrocardiographic (ECG) parameters, and to outline an intraprocedural method to confirm mid-RV nonapical lead positioning.

**Methods:**

Fifty-six patients undergoing implantation of LP with TEE guidance were enrolled in the study. Device position was evaluated by fluoroscopy, ECG, and TEE. Distances between the device and cardiac landmarks were measured by TEE and analyzed with ECG parameters with and without RV pacing.

**Results:**

Mid-RV septal positioning was achieved in all patients. TEE transgastric view (0°–40°/90°–130°) was the optimal view for visualizing device position. Mean tricuspid valve–LP distance was 4.9 ± 0.9 cm, mean pulmonary valve–LP distance was 4.2 ± 1 cm, and calculated RV apex–LP distance was 2.9 ± 1 cm. Mean LP paced QRS width was 160.8 ± 28 ms and increased from 117.2 ± 34 ms at baseline. LP RV pacing resulted in left bundle branch block pattern on ECG and 37.8% QRS widening by 43.5 ± 29 ms.

**Conclusion:**

TEE may guide LP implantation in the nonapical mid-RV position. Further studies are required to establish whether this technique reduces implant complications compared with conventional fluoroscopy.

**Key Findings**

▪ Currently, fluoroscopic imaging is standard practice for leadless pacemaker (LP) implantation, but transesophageal echocardiography (TEE) is a reasonable alternative.
▪ This proof-of-concept study demonstrates the use of TEE cardiac landmarks and electrocardiographic gating to guide optimal LP placement.
▪ We propose a protocol for intraprocedural imaging for LP implantation procedures.
▪ Further studies are required to establish whether this technique reduces implant complications compared with conventional fluoroscopy and/or intracardiac echocardiography.

## Introduction

Fluoroscopy is the standard imaging modality used during permanent pacemaker implantation, including the US Food and Drug Administration–approved leadless pacemaker (LP). The LP established itself as a device to avoid complications common to conventional pacemakers in the evolving world of electrophysiology. By omitting the need for transvenous leads and subcutaneous pocket creation, an LP avoids several complications. Nevertheless, in early experiences there was still a 6.5% risk of serious device-related complications, including cardiac perforation, tamponade, elevated pacing thresholds, and dislodgment.^1^ In 2020, the LP was approved for the treatment of patients with atrioventricular block, making nearly 50% of all pacemaker patients eligible candidates for this leadless system, which will translate to a larger number of patients with leadless devices in our aging population.

This LP system uses 4 self-expanding nitinol tines to anchor onto the right ventricular (RV) myocardium (before 2022). To obtain optimal pacing thresholds at implantation, device repositioning and redeployment may be required. It is recommended that at least 2 tines be engaged in tissue to hold the device securely; this can be done fluoroscopically by utilizing orthogonal views, intravenous (IV) contrast, and a “tug” test in addition to ensuring electrical measurements are within recommended values (pacing threshold <1.0 V at 0.24 ms, pacing impedance 400–1500 V, and R-wave amplitude >5 mV). However, no specific RV positioning had been recommended.^2^

The major complication of apical implantation is RV perforation. Reports have suggested that midseptal implantation may decrease the risk of serious complications.^3^^,^^4^ More patients had the LP implanted in a septal location in the postapproval registry compared to those in the investigational device exemption (IDE) study (52% vs 33%).^5^, ^6^, ^7^ Little guidance is currently available regarding alternative intraprocedural imaging other than conventional fluoroscopy. In traditional pacemaker systems, generic fluoroscopic RV septal implantation criteria have proven to be unreliable with RV leads placed in the free wall despite being thought to be positioned in the septum via fluoroscopy.^8^, ^9^, ^10^ For this reason, the definition of septal implantation in the literature may not be entirely accurate, and a broader term of “RV nonapical pacing” has been suggested.^11^ This raises major concerns about the safety and efficacy of relying solely on fluoroscopic criteria for lead positioning.

Helpful anatomic landmarks for implantation, such as the tricuspid valve (TV) and pulmonary valve (PV), cannot be visualized fluoroscopically. The purpose of this study was to establish the spatial relationship of the LP to cardiac landmarks via transesophageal echocardiography (TEE) and their correlations with electrocardiographic (ECG) parameters, and to outline a reproducible intraprocedural method to confirm mid-RV septal lead positioning.

## Methods and materials

This study was a single-center observational retrospective electronic medical record review of patients who underwent LP implantation in the electrophysiology laboratory of University of California Davis Medical Center (UCDH) from February 2019 to February 2022. The study was approved by the institutional review board at UCDH and adhered to the Helsinki Declaration in human research as revised in 2013.

Inclusion criteria were all adult patients undergoing intraprocedural TEE-guided LP implantation between February 2019 and February 2022. All LP implantation guided only by fluoroscopy were excluded. All devices were Micra (Medtronic, Minneapolis, MN) pacemakers. All implantations were performed using the Philips FD 10-10 fluoroscopic system (Philips, Andover, MA). The Siemens SC2000 system was used for TEE. The GE CardioLab recording system was used for standard 12-lead ECG monitoring during implantation. All intraprocedural ECG data were processed with the CardioLab data station (GE, Waukesha, WI), and all TEE imaging data were processed with the Syngo dynamic system (Siemens, Malvern, PA).

**Figure 1:**
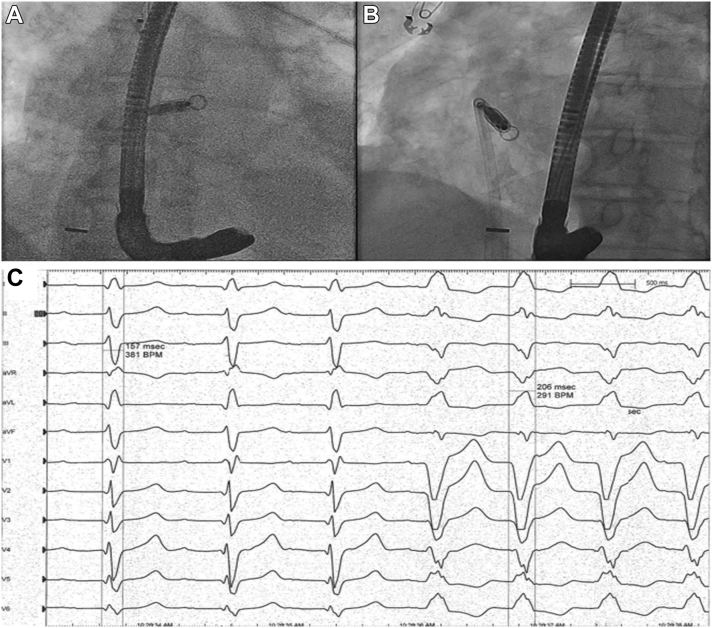
Standard fluoroscopic views of leadless pacemaker (LP) implantation: anteroposterior **(A)** and left anterior oblique 40° **(B)** views. **C:** Standard 12-lead electrocardiographic monitoring. QRS complex width was measured before (first 3 QRS complexes) and after (last 4 QRS complexes) LP pacing.

**Figure 2:**
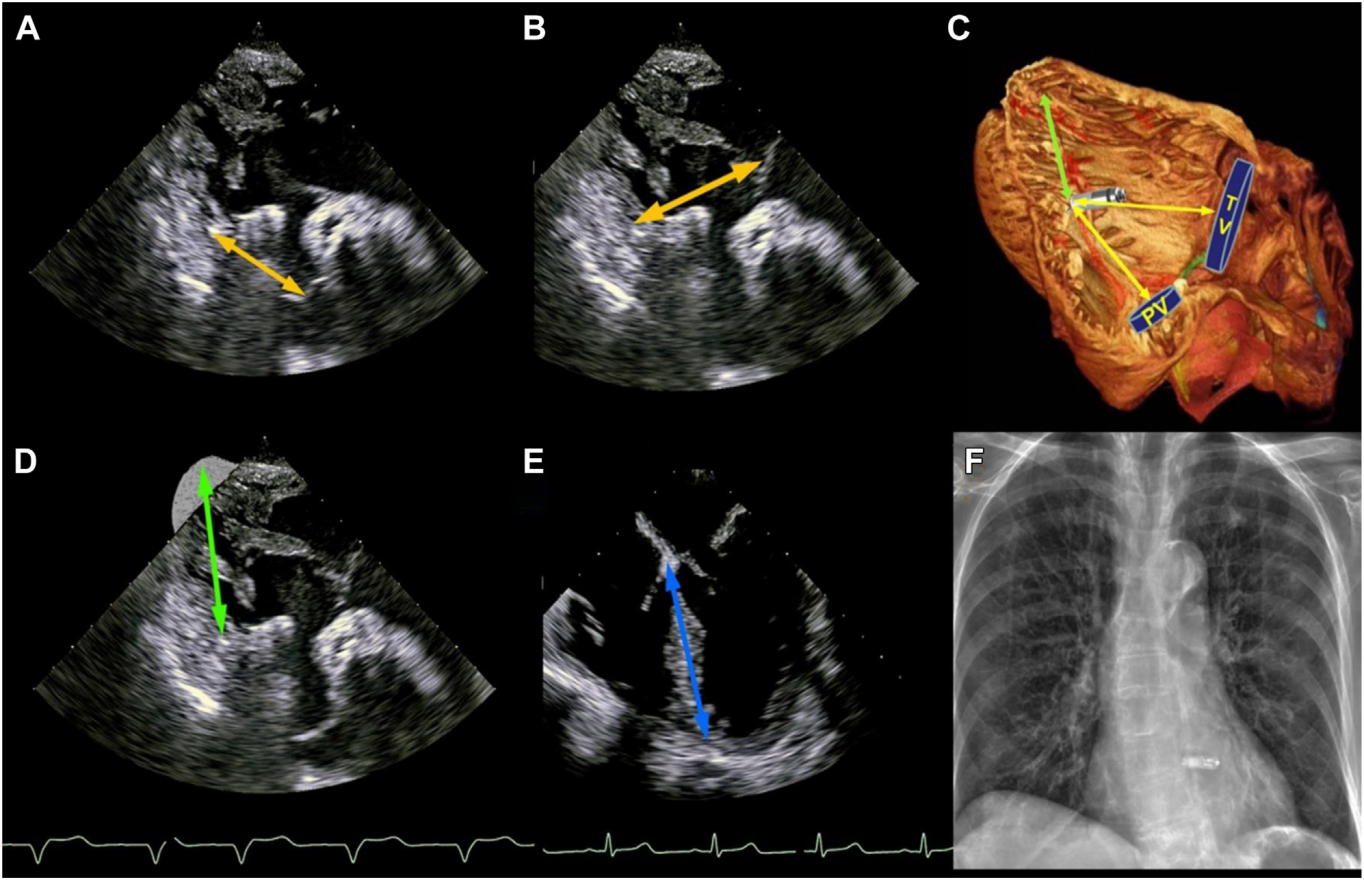
Transesophageal echocardiographic images in the deep gastric view (130°–140°) with obtained PV-LP **(A),** TV-LP **(B),** derived RV apex-LP **(D),** as illustrated **(C),** and IVS length measurement in the midesophageal view at 0° **(E). F:** Postimplantation x-ray film. IVS = intraventricular septum; LP = leadless pacemaker; PV = pulmonary valve; RV = right ventricle; TV = tricuspid valve. (Figure 1C was adapted from Stephenson RS, Atkinson A, Kottas P, et al. High resolution 3-dimensional imaging of the human cardiac conduction system from microanatomy to mathematical modeling. Sci Rep 2017;7:7188.)

The procedures were performed with patients in the fasting state following standard protocol utilizing informed consent and under general anesthesia.^12^ The position and stability of the LP were confirmed fluoroscopically during the procedure by using orthogonal views (Figure 1), IV contrast, and a “tug” test in all patients. All TEEs were performed using a standard TEE protocol (Supplemental Appendix) at UCDH by 1 echo board-certified cardiologist as the second proceduralist. Postoperative posteroanterior/lateral x-ray films were taken for all patients before discharge (Figure 2F). Twelve-lead ECG data were obtained for every patient before and after pacing. Using multichannel ECGs, all 12 leads were lined up in a time-synchronized manner. QRS width was measured from the earliest deflection point of any chest or limb lead to the latest deflection point of any chest or limb lead. This gives the highest and most consistent values of the QRS duration and is more accurate than those obtained by conventional measurements of any individual chest or limb lead.^13^

The primary outcomes of interest were TV-LP distance, PV-LP distance, and RV apex–LP distance (in centimeters), post–LP pacing QRS width (in milliseconds), and change in paced QRS width (in percentage). Secondary outcomes included all immediate intraprocedural complications. Adopting identical criteria as in the Micra IDE study, major complications were defined as system- and procedure-related events resulting in death, permanent loss of device function, hospitalization, prolonged hospitalization by 48 hours, or system revision.^7^

The following data were extracted: date of procedure, indication, patient’s age, gender, left ventricular ejection fraction (before pacing) and comorbidities. QRS complex width pre- and post-LP pacing were recorded by the same operator on the CardioLab data station (Figure 1C). The TEEs were individually reviewed by the same operator, and the following measurements were obtained: TV-LP distance and PV-LP distance were measured in the transgastric 0°–40°/90°–130° views. The measurements were obtained from the tip of the LP to the delineated cardiac landmarks (center of coaptation of the PV in diastole [Figure 2A] and the TV in systole [Figure 2B]). Intraventricular septum (IVS) length was measured in the midgastric 4 chamber 0°–20° view at end-diastole from the TV septal leaflet insertion to the RV apex (Figure 2E). The RV apex–LP distance was calculated by subtraction (IVS – ½[TV-LP + PV-LP]) (Figure 2D).

All measurements and numerical data are given as mean ± SD for continuous variables, and categorical variables are summarized as frequency (%). The paired *t*-test was used to compare continuous variables accounting for pairing as needed (eg, for pre- vs post-data within the same patient or for 2 independent groups), and the correlation between variables was studied using Pearson correlation coefficient. These parametric methods may be well justified because the data are reasonably symmetric. SAS Version 9.4 (SAS Institute, Cary, NC) was used for data analyses.

## Results

### Patient population

A total of 56 patients with procedural TEEs were included in the study. Demographic, clinical, and procedural characteristics are listed in Table 1. Average age of the patients was 74.1 ± 12 years, and 64% of the participants were male. Average left ventricular ejection fraction was 53.6% ± 9%. Average IVS length was 7.5 ± 1.2 cm. Baseline characteristics and medical treatments of the 56 consecutive patients who received a LP system are summarized in Table 1.

**Table 1:** Demographic characteristics (N = 56)

Age (y)	74.1 ± 13
Male	35 (64)
Indication for pacing	
Grade 2 or 3 atrioventricular block	31 (55)
Sick sinus syndrome	27 (48)
Comorbidities	
Hypertension	44 (78)
Ischemic heart disease	19 (34)
Previous myocardial infarction	10 (18)
Chronic kidney disease	25 (45)
Diabetes	19 (34)
Heart failure	22 (39)
Left ventricular ejection fraction (%)	53.6 ± 9

Values are given as mean ± SD or n (%).

### ECG changes

Average QRS width was 117.2 ± 34 ms at baseline, and average QRS width post-LP pacing was 160.8 ± 28 ms. LP pacing resulted in mean QRS widening by 43.5 ± 29 ms (43.9% ± 33%; *P* <.01). Correlation between baseline and post-LP pacing QRS width was 0.57 (*P* <.01). This suggested the wider the QRS at baseline, the wider the QRS post-LP pacing. Correlation between baseline QRS width and IVS length was 0.41 (*P* <.01). Correlation between post–LP pacing QRS width and IVS length was 0.25 (*P* = .06). This suggested the wider the QRS at baseline as well as postpacing, the longer the IVS (Table 2).

**Table 2:** Pearson correlation coefficients of the obtained measurements with *P* value (N = 56)

	Inherent QRS	Paced QRS	IVS length (TEE)	TV-LP (TEE)	PV-LP (TEE)	LP–apex average (TEE)
Native QRS	1	0.57	0.41	0.31	0.32	0.18
		(<.0001)	(0.002)	(0.02)	(0.02)	(0.18)
Paced QRS		1	0.25	0.28	0.48	–0.07
			(0.06)	(0.04)	(0.0002)	(0.61)
IVS length (TEE)			1	0.51	0.47	0.72
				(<.0001)	(0.0003)	(<.0001)
TV-LP (TEE)				1	0.57	–0.12
					(<.0001)	(0.36)
PV-LP (TEE)					1	–0.20
						(0.13)

IVS = intraventricular septum; LP = leadless pacemaker; PV = pulmonary valve; TEE = transesophageal echocardiography; TV = tricuspid valve.

### TEE measurements

Average TV-LP distance was 4.9 ± 0.9 cm, and average PV-LP distance was 4.2 ± 1 cm. Average RV apex–LP distance was 2.9 ± 1 cm. The correlation between baseline QRS width and TV-LP was 0.31 (*P* = .02), and the correlation between post–LP pacing QRS width and TV-LP was 0.28 (*P* = .04). The correlation between baseline QRS width and LP-PV was 0.32 (*P* = .02), and the correlation between post–LP pacing QRS width and PV-LP was 0.48 (*P* <.01). The correlation between baseline QRS width and RV apex–LP distance was 0.17 (*P* = .21), and the correlation between post–LP pacing QRS width and RV apex–LP distance was 0.07 (*P* = .61). The correlation between IVS and RV apex–LP distance was 0.72 (*P* <.01).

### Follow-up

Patients were discharged an average of 2.9 ± 4 days following implantation, after normal function of the pacemaker was confirmed and a chest radiograph was obtained. Follow-up was performed an average of 16.5 ± 18 days after the procedure in the outpatient pacemaker clinic.

## Discussion

This proof-of-concept study demonstrated that TEE as a supplemental imaging modality to guide LP implantation is feasible. The LP guide catheters and the cardiac markers (TV, PV, and papillary muscles) were adequately visible and beneficial for localization throughout the procedure. During the study period, a total of 77 LP implantations (38 VR and 39 AV) were performed in our institution, and 21 of the implantations used only fluoroscopy, most of which were during the initial phase of the procedural implantation at our institution, so we did not perform comparisons between the fluoroscopy-guided only cohort and the TEE/fluoroscopy–guided cohort because the numbers were too small and learning curves of the implanters were skewed.

To the best of our knowledge, this was the first time intraprocedural TEE was used to define the TV-LP, PV-LP, and RV apex–LP distances. These parameters had several advantages over use of contrast to determine the RV nonapical location. First, using the TV-LP and PV-LP distances allowed the operator to determine how far was the LP device from the RV apex. This was crucial because the RV apex is the thinnest part of the RV and is most vulnerable to perforation. It provided an additional layer of monitoring for intraprocedural complications during LP placement. Second, we found the septal papillary muscle of the TV apparatus was reliably visualized in the majority of cases in the gastric 90°–130° RV long-axis view. This important landmark on the RV side of the IVS could serve as an important landmark for localization of the septum.^14^ We used this septal papillary muscle as the reference point but did not aim to implant the LP onto the papillary muscle because it might reduce stability and negatively influence the LP pacing parameters. Numerically, the LP was found to be closer to the PV (4.2 cm) than the TV (4.9 cm), which is in keeping with suggested nonapical implantation. Third, we found that the TEE transgastric (0°–40°/90°–130°) biplane view was the optimal view to visualize device position, especially the LP relationship with the IVS and RV apex.

We used TEE to assist LP implantation with patients under general anesthesia only. The type of sedation used for LP implantation was decided jointly by the patients and procedural physicians clinically. During the study period, most of the LP implantations were performed with patients under general anesthesia to ensure patient safety because it was a new procedure to our institution. As the procedure evolved, procedural sedation transitioned from general anesthesia to monitored anesthesia care sedation (ie, conscious sedation). TEE might cause additional patient discomfort, and assessment of risks/benefits needs to be evaluated on an individual basis. Alternatively, intracardiac echocardiography (ICE) might be used to guide the procedure. In our 3 patients in whom ICE was used, we found that the RV septum and LP device were best visualized with ICE with the ICE probe in the RV cavity instead of the right atrium (data not shown); however, the numbers were too small for comparison. Traditionally, right anterior oblique/left anterior oblique orthogonal views in fluoroscopy are used for pacemaker lead positioning within the IVS, and IV contrast is used to visualize RV landmarks such as the RV apex.^15^ We used IV contrast in the majority of our cases besides intraprocedural TEE, yet the final LP position in relation to the fluoroscopic RV landmarks was difficult to quantitate, so we did not perform any comparison of TEE RV-LP landmarks and fluoroscopic RV landmarks.

Although the midesophageal 60°–90° short-axis view allowed visualization of the LP, TV, and PV in the same 2-dimensional imaging plane, the IVS was not visible in this view.^16^ Although The midesophageal 0°–30° 4-chamber view provided good visualization of the IVS, the LP and the pulmonic valve were rarely visible, and the pulmonic valve usually was absent.^12^ Finally, intraprocedural TEE allowed real-time monitoring for early identification of iatrogenic pericardial effusion, if present.

The periprocedural complication rate of LP implantation was low, with pericardial effusion or tamponade observed in 1%–2% of cases.^6^^,^^9^ Previously described septal positioning of the LP device was suggested to have a favorable effect on the risk for perforation.^6^^,^^7^ However, in many relevant studies, devices that were thought to be septally implanted under fluoroscopy were implanted in the RV apex.^6^^,^^9^^,^^11^^,^^17^ Surprisingly, in 4 of the 5 patients with LP-related pericardial effusion or tamponade, the devices were implanted in the septal position.^11^ In a study by Kaczmarek et al,^16^ who investigated septal implantations of LP devices, a single heart perforation occurred when a delivery sheath was directed to the free wall of the RV. Previous experience with conventional pacemakers suggests that fluoroscopy might be insufficient for guiding precise lead implantation in the IVS; therefore, use of intraprocedural TEE seems to be advisable.^3^^,^^18^ Contrary to the study by Kaczmarek et al,^16^ who suggested that an upper to midesophageal position of the TEE probe was the best location to visualize and facilitate septal positioning of the LP during implantation, our study favored the TEE transgastric (0°–40°/90°–130°) biplane view because it was reproducible in all study participants.

RV pacing–induced QRS widening is a well-known phenomenon. In our study, we found no statistical correlation between the TEE-defined TV-LP, PV-LP, and LP-apex distances and the native and paced QRS complexes. However, our study showed that the wider the native QRS at baseline, the wider the QRS post–LP pacing. This was consistent with the notion that the intraventricular conduction system disease is a diffuse process, and that the preexisting conduction system disease is an important contributor to determining the postpacing QRS width. Moreover, there is a significant QRS axis shift post–LP pacing. Preliminary analysis of our study cohort demonstrated that LP pacing resulted in a shift of the QRS complexes rightward in the frontal plane and posteriorly in the horizontal plane (data not shown). Further analysis of the relationship of LP anatomic position and QRS shift is under way.

Lastly, the average length of hospital stay was 2.9 days post–LP implantation, which was longer than expected. This likely was due to the higher proportion of inpatients in our study cohort.

### Study limitations

Several limitations restrict the generalizability of the study results. First, this was a retrospective study of a small sample with inherent biases. Because of the small sample size, there was a risk of type II statistical error, and we could not perform adjusted analyses. In addition, there was no control group of patients who underwent fluoroscopy-only implantation of LP to serve as a comparison; therefore, no clinical inference can be made. Given this was a single-center study, there was an inherent lack of external validity to support widespread changes in clinical practice. The study duration was short, and the number of participants was too small to detect rare, previously reported complications. Finally, we were unable to account for repositioning due to the retrospective nature of the study and nuances in reporting repositioning in procedural notes.

## Conclusion

This study is the first report of a systematic intraprocedural TEE protocol to guide nonapical mid-RV implantation of LPs. Important cardiac landmarks to LP distances (which are considered surrogates for midventricular and nonapical implantation) were measured and reported. The study suggests that TEE may be useful to navigate midventricular and nonapical implantation of LPs while avoiding perforation. Our preliminary study proposed a method to protocolize intraprocedural imaging for LP implantation procedures. Use of ICE may be considered on a case-by-case basis to avoid risks of general anesthesia. Larger studies comparing the different echocardiographic modalities to traditional fluoroscopy are needed to conclusively determine the cost-effectiveness and patient safety concerns of utilizing various imaging modalities to guide LP implantation. More data are needed to determine whether TEE will lead to fewer intraprocedural complications.

## Appendix

Updated Appendix

## References

[1] Reddy V.Y., Exner D.V., Cantillon D.J. (2015). Percutaneous implantation of an entirely intracardiac leadless pacemaker. N Engl J Med.

[2] Ritter P., Duray G.Z., Steinwender C. (2015). Early performance of a miniaturized leadless cardiac pacemaker: the Micra Transcatheter Pacing Study. Eur Heart J.

[3] Hai J.J., Fang J., Tam C.C. (2019). Safety and feasibility of a midseptal implantation technique of a leadless pacemaker. Heart Rhythm.

[4] Kirkfeldt R.E., Johansen J.B., Nohr E.A., Jorgensen O.D., Nielsen J.C. (2014). Complications after cardiac implantable electronic device implantations: an analysis of a complete, nationwide cohort in Denmark. Eur Heart J.

[5] Hsu J.C., Varosy P.D., Bao H., Dewland T.A., Curtis J.P., Marcus G.M. (2013). Cardiac perforation from implantable cardioverter-defibrillator lead placement: insights from the National Cardiovascular Data Registry. Circ Cardiovasc Qual Outcomes.

[6] Duray G.Z., Ritter P., El-Chami M. (2017). Long-term performance of a transcatheter pacing system: 12-month results from the Micra Transcatheter Pacing Study. Heart Rhythm.

[7] Reynolds D.W., Ritter P. (2016). A leadless intracardiac transcatheter pacing system. N Engl J Med.

[8] Sharma G., Salahuddin S., Sanders P. (2016). Inadequacy of fluoroscopy and electrocardiogram in predicting septal position in RVOT pacing–validation with cardiac computed tomography. Indian Heart J.

[9] Pang B.J., Joshi S.B., Lui E.H. (2014). Validation of conventional fluoroscopic and ECG criteria for right ventricular pacemaker lead position using cardiac computed tomography. Pacing Clin Electrophysiol.

[10] Mǎrgulescu A.D., Şuran B.M., Rimbaş R.C., Dulgheru R.E., Silişte C., Vinereanu D. (2012). Accuracy of fluoroscopic and electrocardiographic criteria for pacemaker lead implantation by comparison with three-dimensional echocardiography. J Am Soc Echocardiogr.

[11] Shimony A., Eisenberg M.J., Filion K.B., Amit G. (2012). Beneficial effects of right ventricular non-apical vs. apical pacing: a systematic review and meta-analysis of randomized-controlled trials. Europace.

[12] Hahn R.T., Abraham T., Adams M.S. (2013). Guidelines for performing a comprehensive transesophageal echocardiographic examination: recommendations from the American Society of Echocardiography and the Society of Cardiovascular Anesthesiologists. J Am Soc Echocardiogr.

[13] Lepeschkin E., Surawicz B. (1952). The measurement of the duration of the QRS interval. Am Heart J.

[14] Minguito-Carazo C., Benito-González T., Estévez-Loureiro R. (2021). Interventional Treatment for Structural Heart Disease.

[15] Das A., Kahali D. (2018). Ventricular septal pacing: optimum method to position the lead. Indian Heart J.

[16] Kaczmarek K., Cygankiewicz I., Czarniak B. (2019). Septal implantation of the Micra transcatheter pacing system guided by intraprocedural transesophageal echocardiography. Kardiol Pol.

[17] El-Chami M.F., Al-Samadi F., Clementy N. (2018). Updated performance of the Micra transcatheter pacemaker in the real-world setting: a comparison to the investigational study and a transvenous historical control. Heart Rhythm.

[18] El-Chami M., Roberts P., Kypta A. (2016). How to implant a leadless pacemaker with a tine-based fixation. J Cardiovasc Electrophysiol.

