# Supplementary material for: Exploratory use of intraprocedural transesophageal echocardiography to guide implantation of the leadless pacemaker
Source: Heart Rhythm O2. 2022 Oct 18;4(1):18–23. doi: 10.1016/j.hroo.2022.10.005 (PMC9877395; doi:10.1016/j.hroo.2022.10.005)
Supplement: Updated Appendix [file mmc1.pdf]

Echocardiography in Micra Leadless Pacemaker Implantation

A Visual Guide

Viewpoints of TEE in Leadless Pacemaker Implantation

- Guidewire/Sheath Deployment
- The Delivery System
- Pre-Delivery: Anchor Location Optimization
- Deployment
- Tether (“Tug) Test/ Tether Removal
- Monitor and Measure Micra Position Post-Final Implant

Guidewire/Sheath Deployment

- Guidewire/Sheath Deployment
  - Mid-esophageal bicaval view observing IAS, IVC, SVC
  - Guidewire
    - Observe guidewire advancing form the IVC to SVC with Curls observed in SVC
    - Confirm wire is deep in SVC prior to proceeding with sheath
  - Sheath
    - After advancing sheath to appropriate position with wire, remove wire and confirm sheath position
    - Important to observe sheath position in view as sheath can easily cause penetrating complication to IAS, RA, or RV free wall.

The Guide Wire

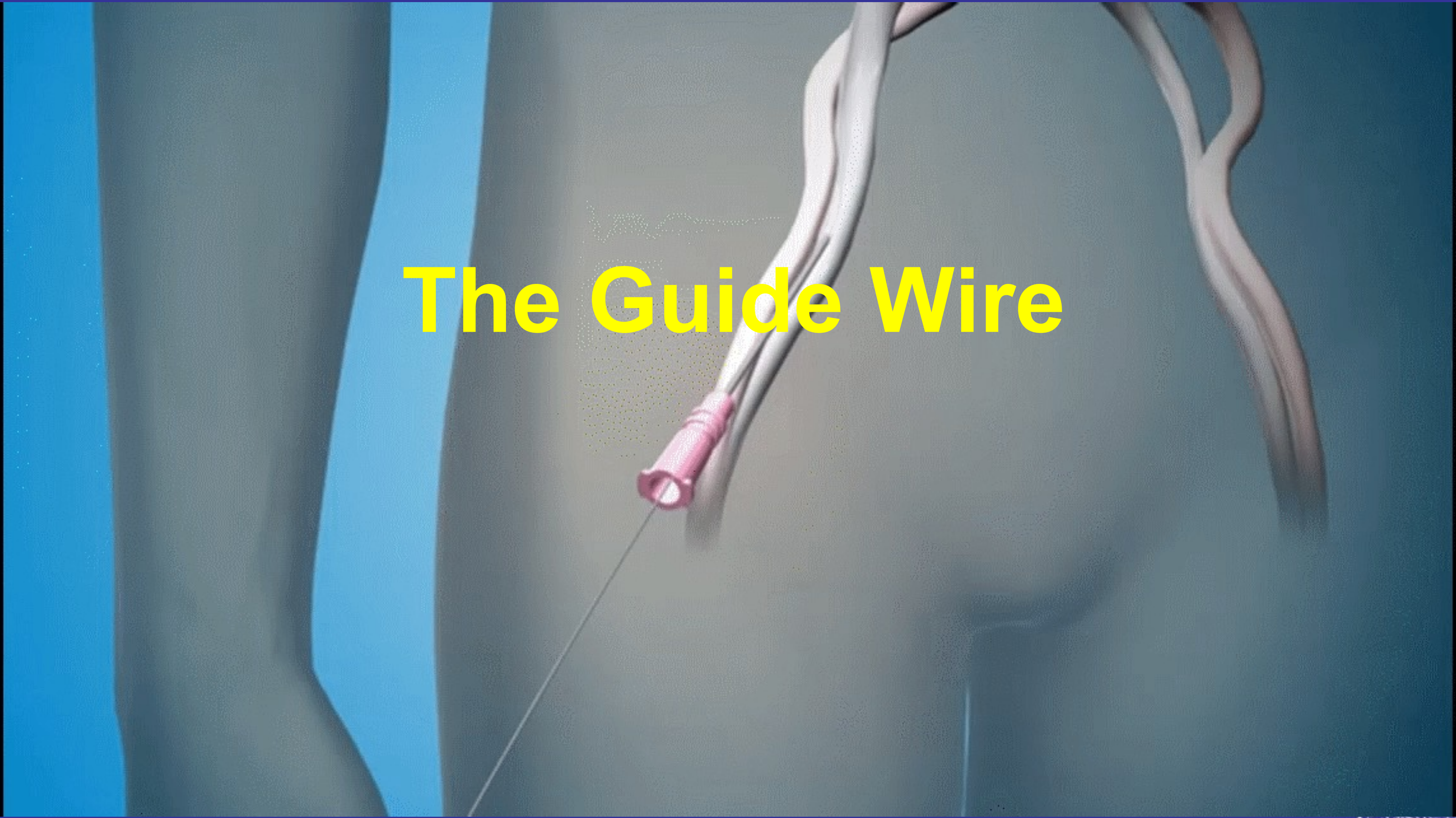

Wire From IVC into SVC

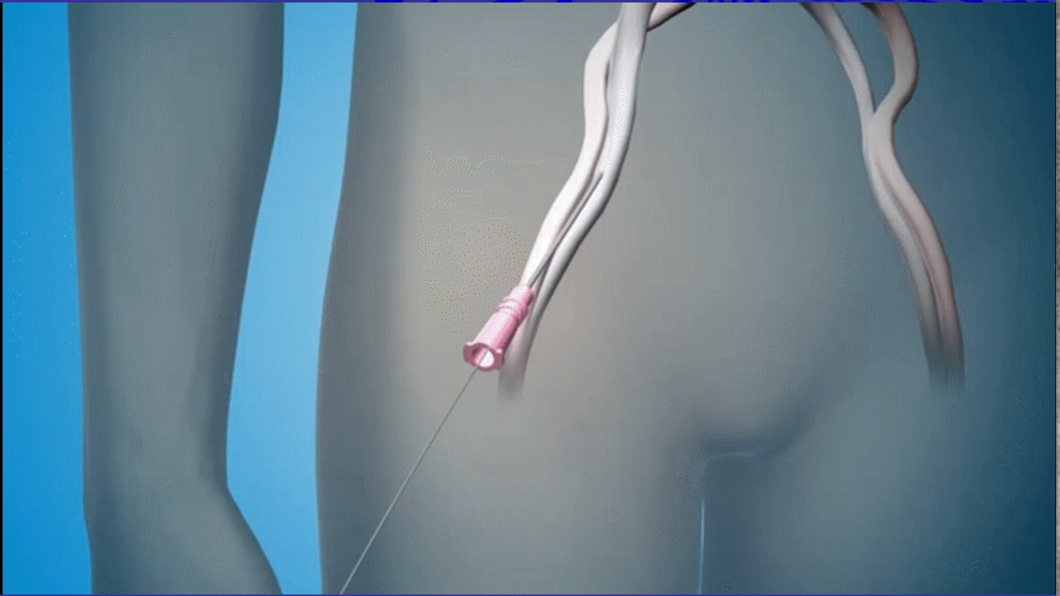

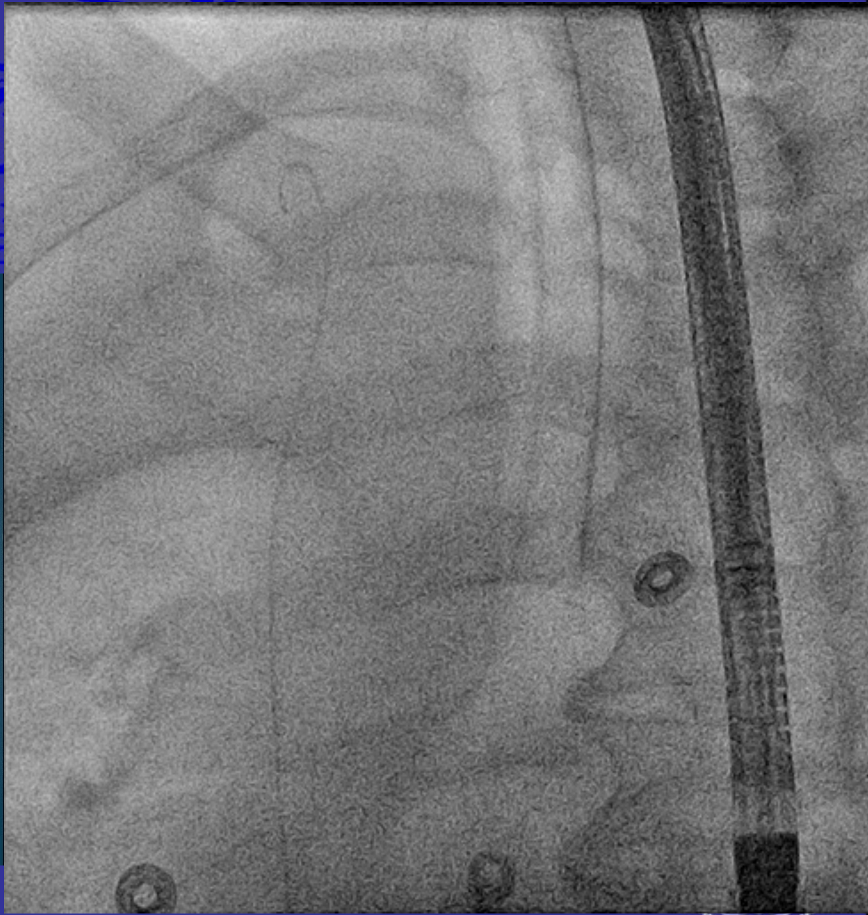

Wire From IVC into SVC

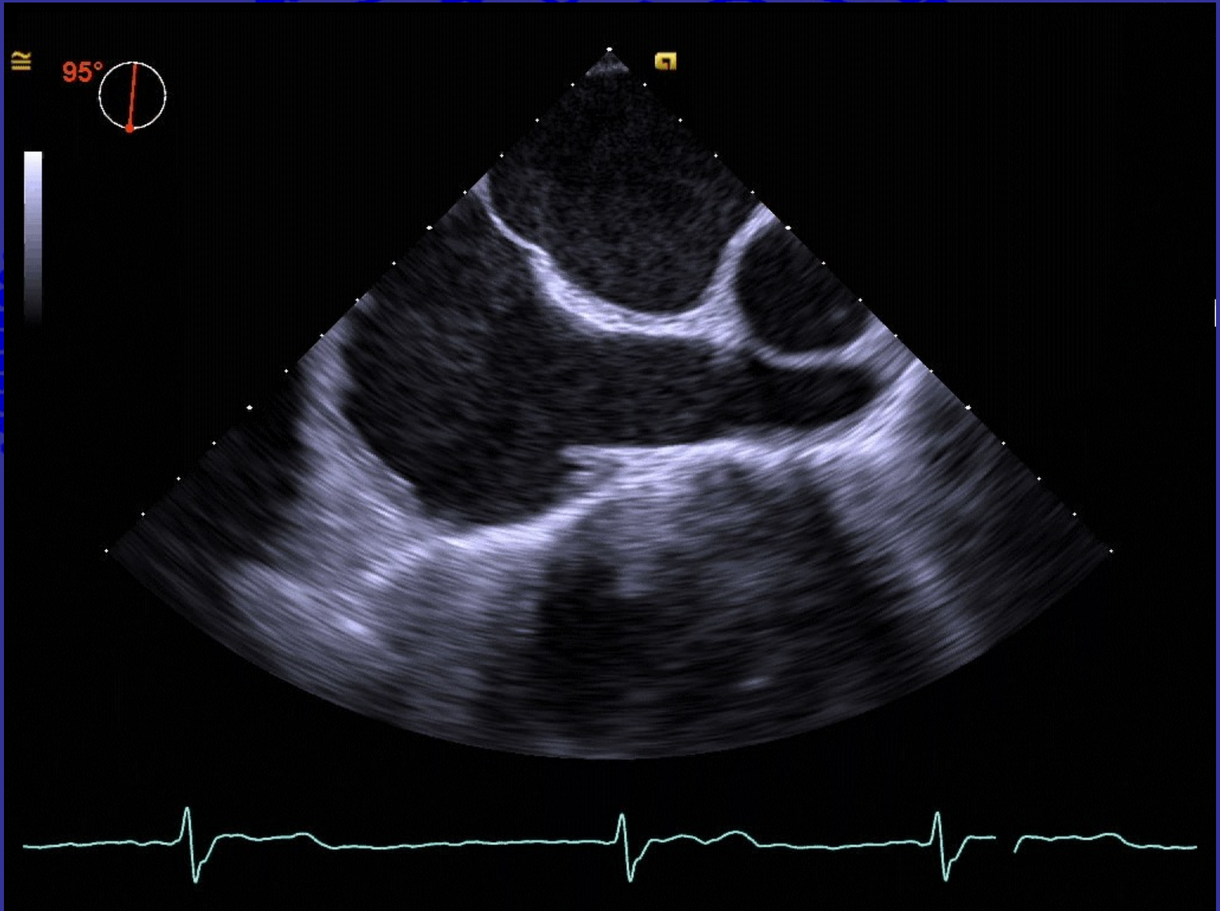

Wire From IVC into SVC

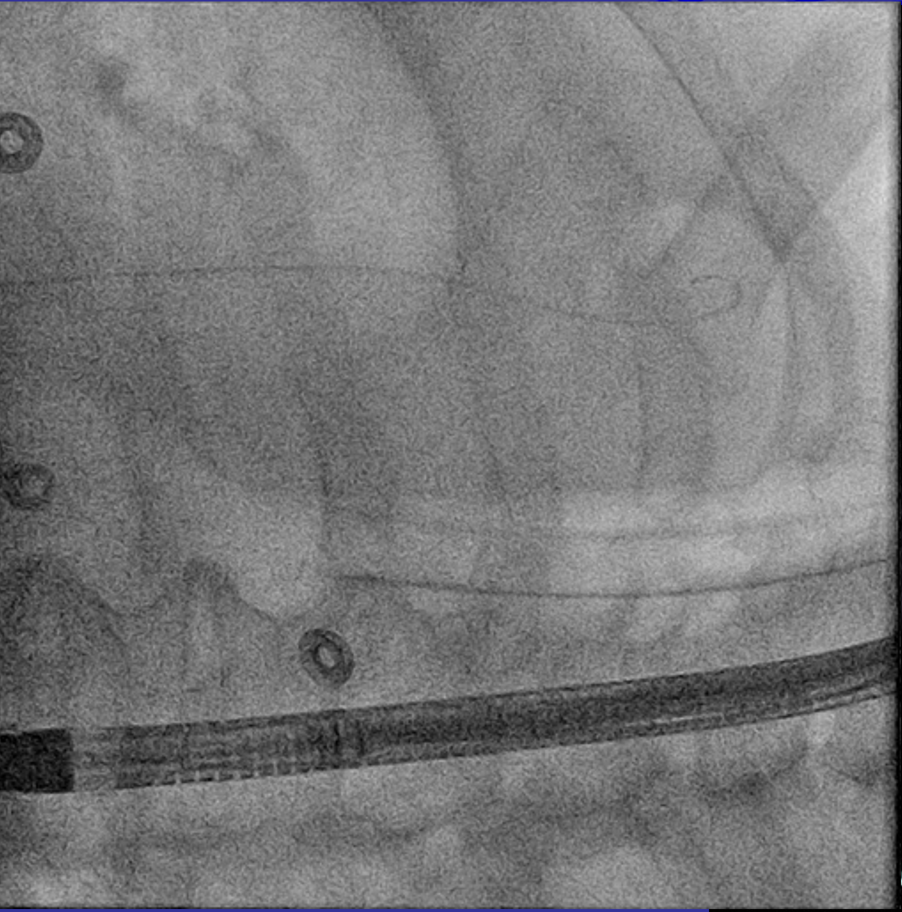

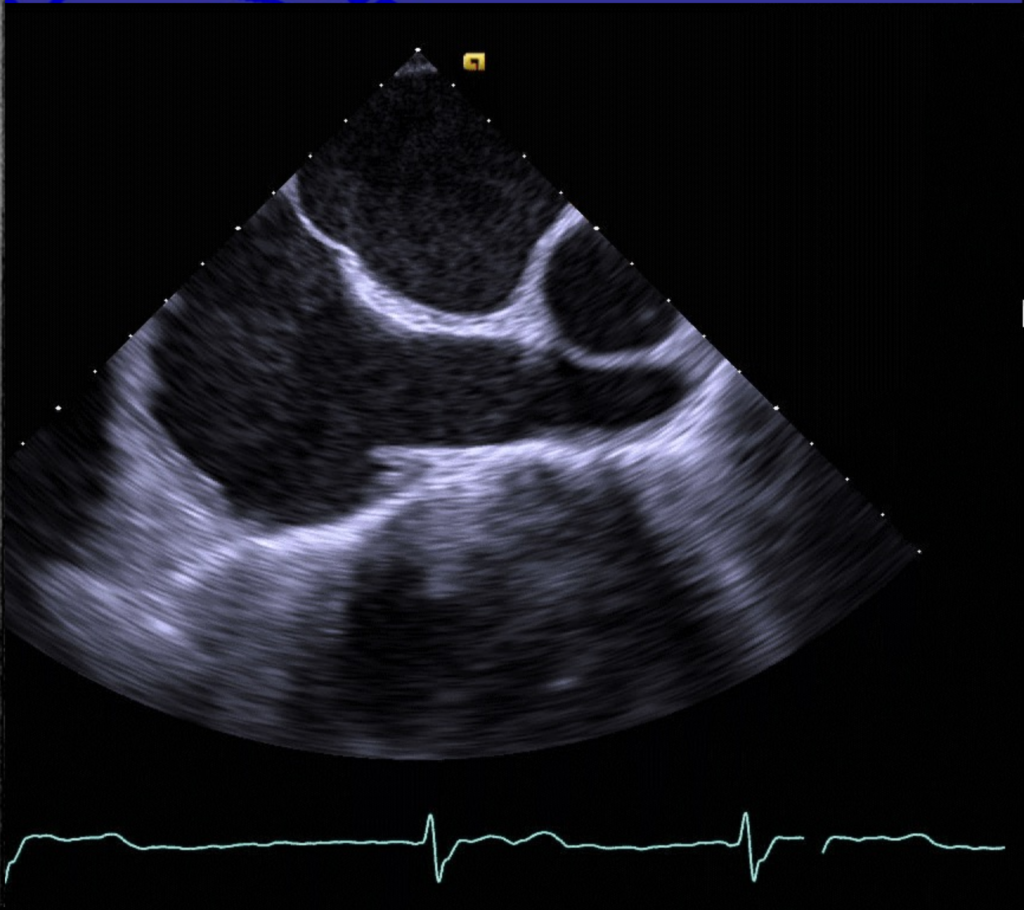

Wire From IVC into SVC, curl 1

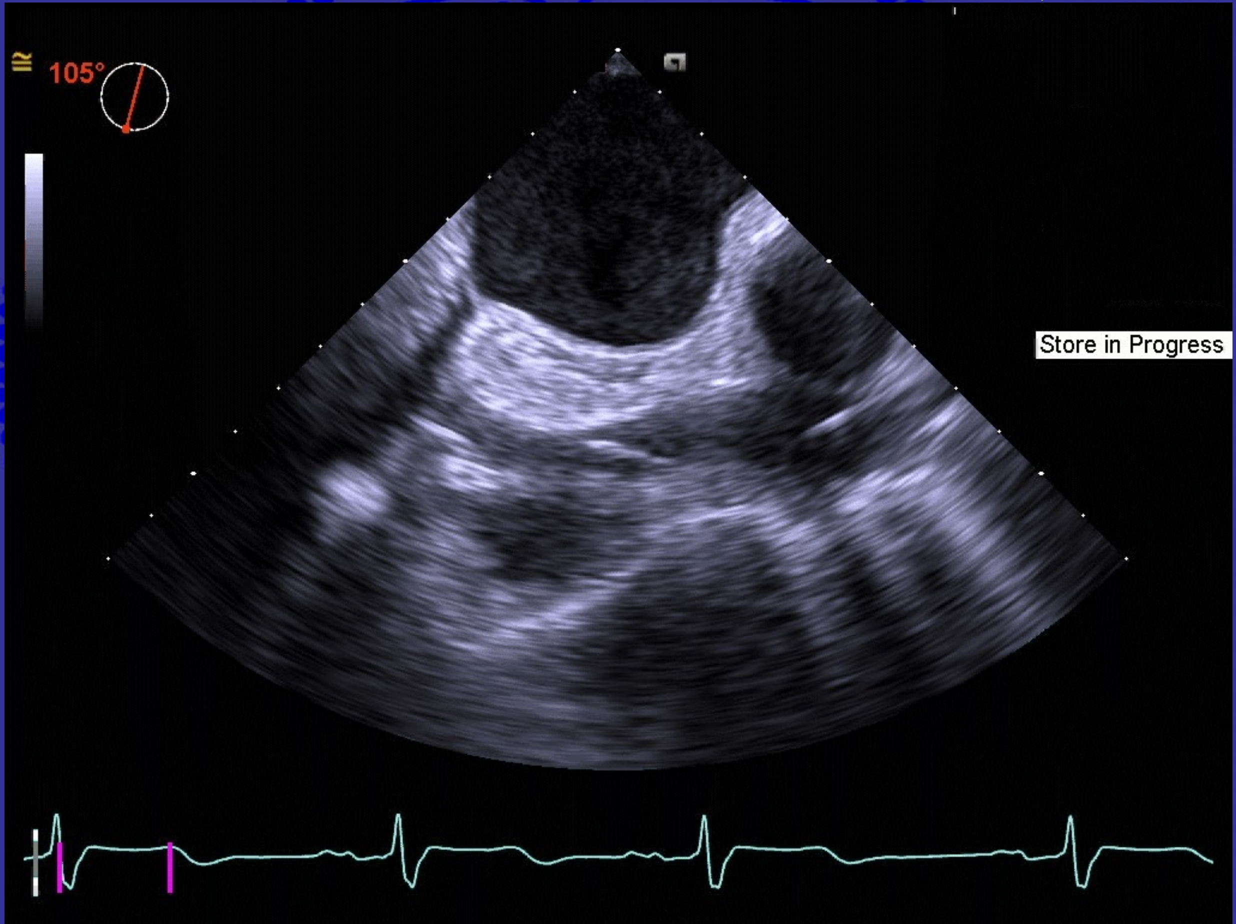

Wire From IVC into SVC, curl 2

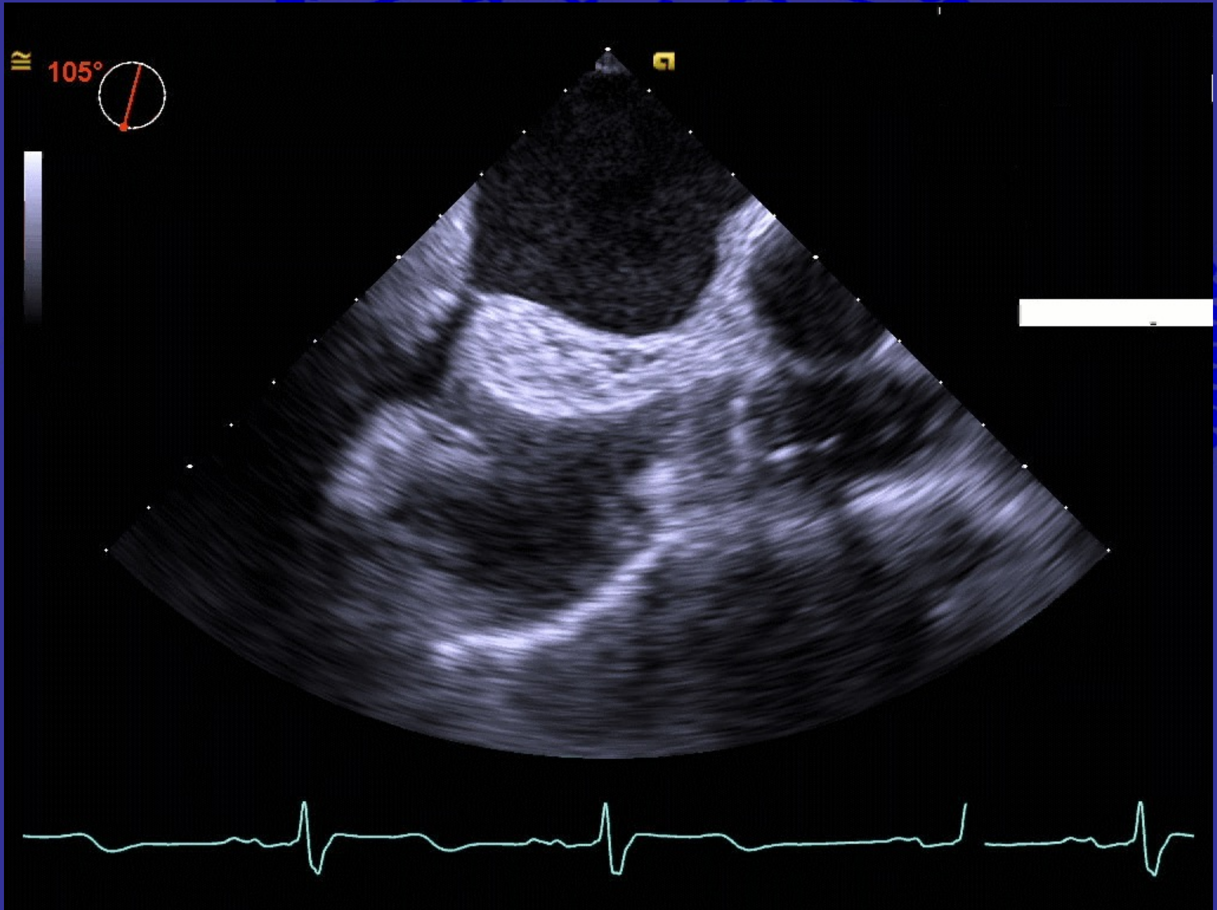

Wire From IVC Deep into SVC

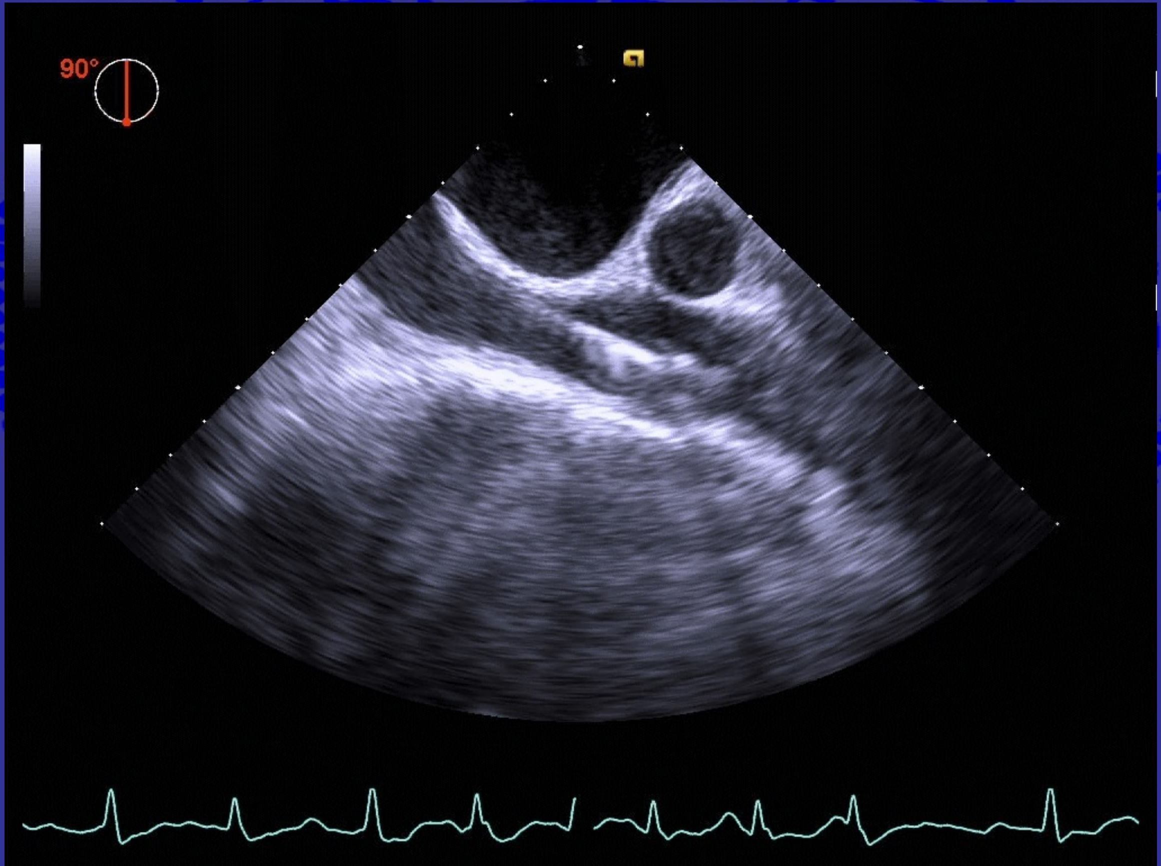

The Sheath

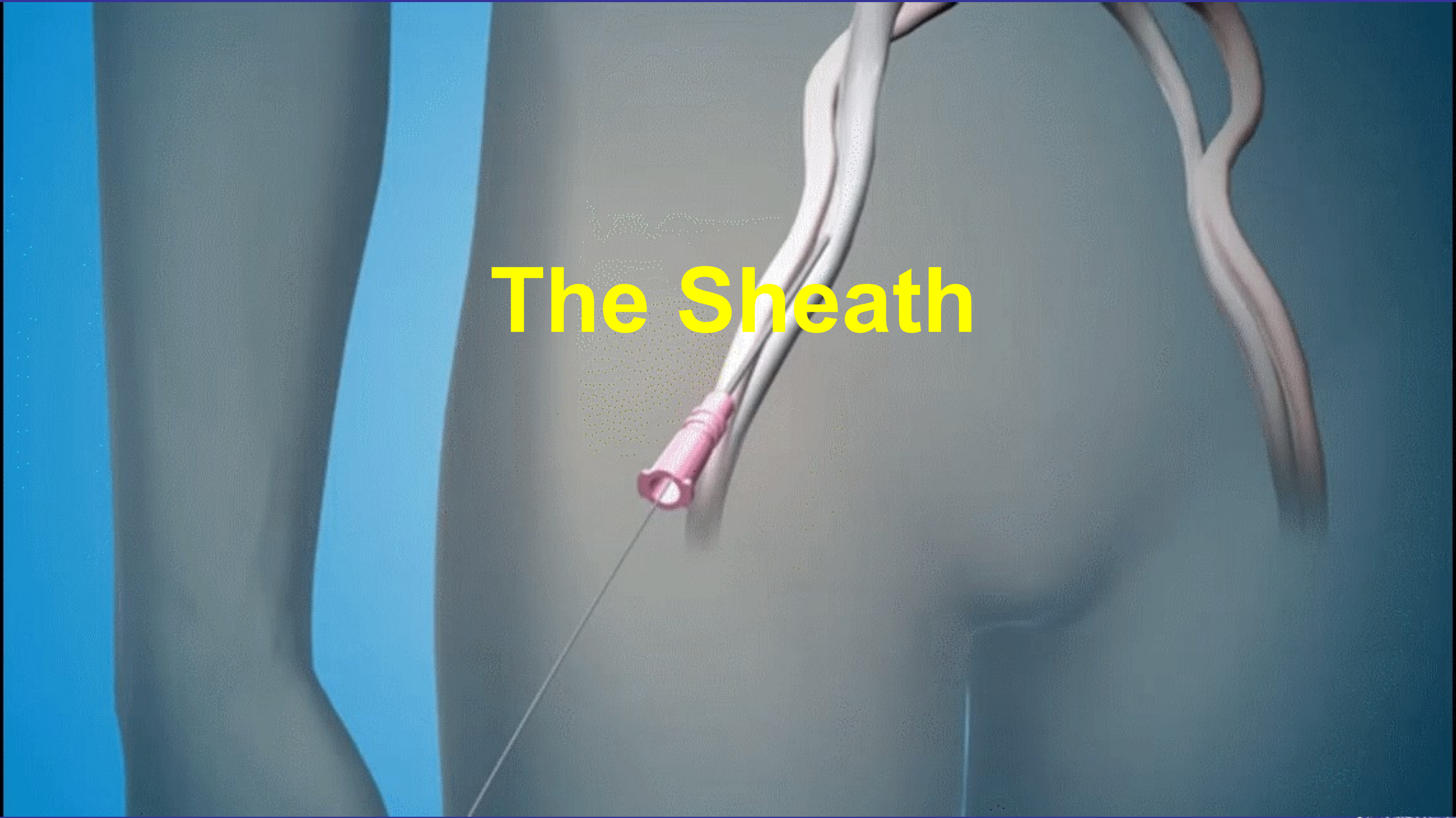

Advancing Sheath without wire

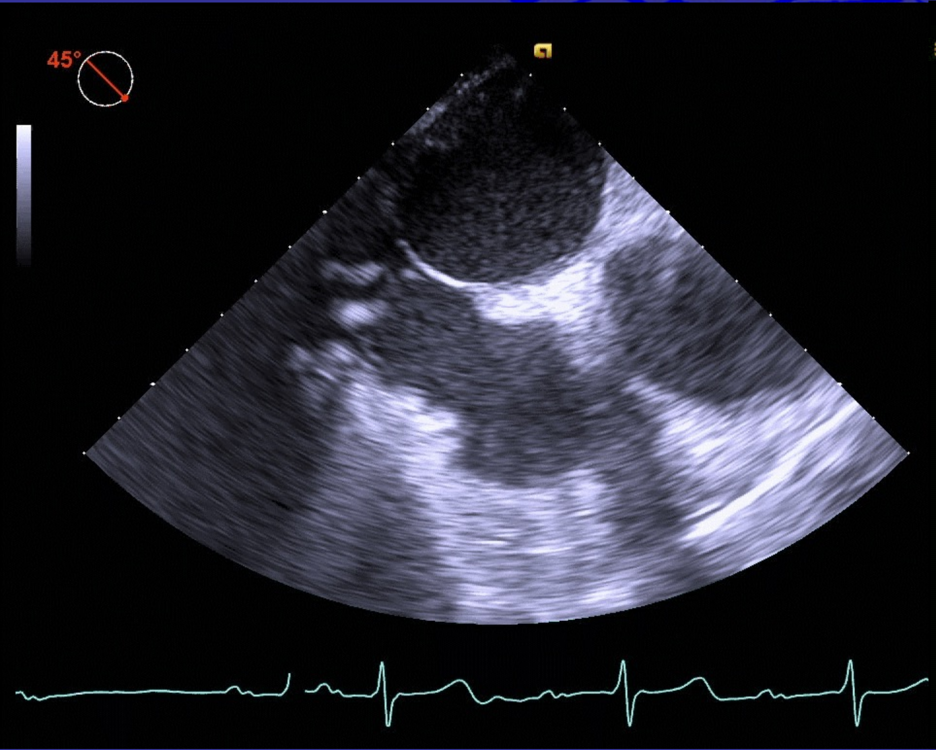

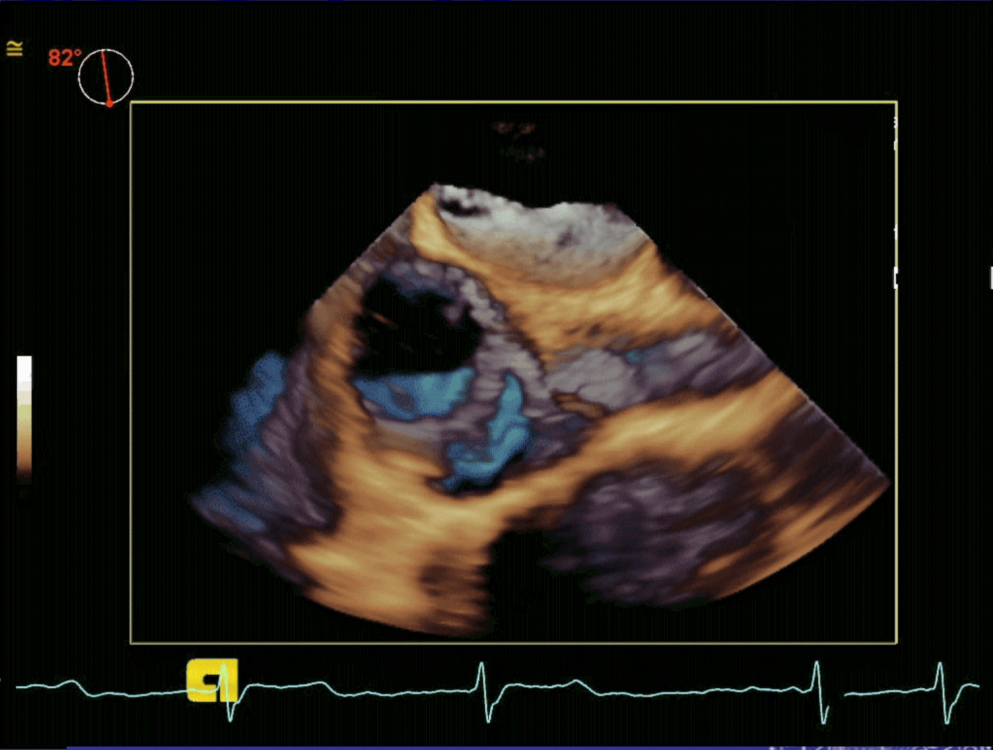

Advancing Sheath touching IA septum with a short pause

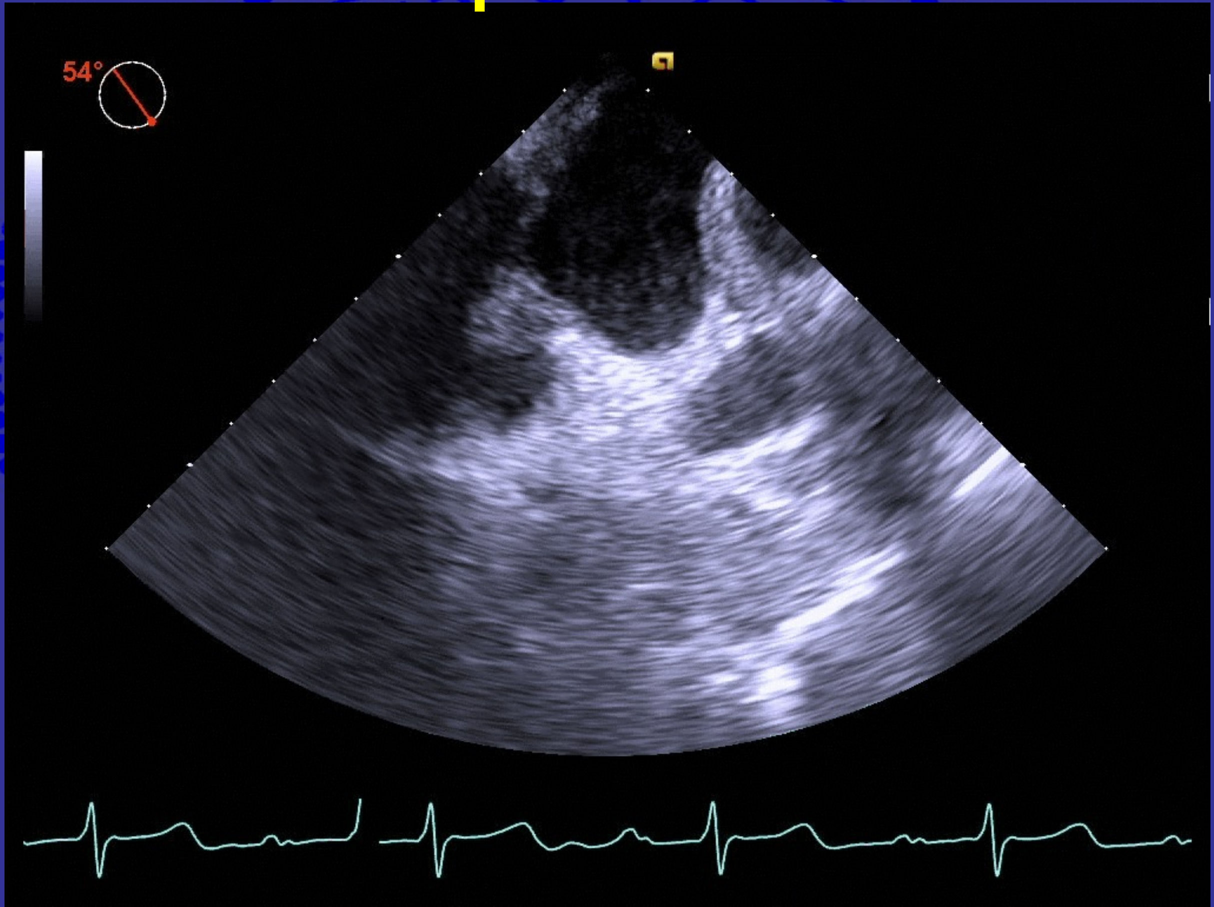

Sheath going in

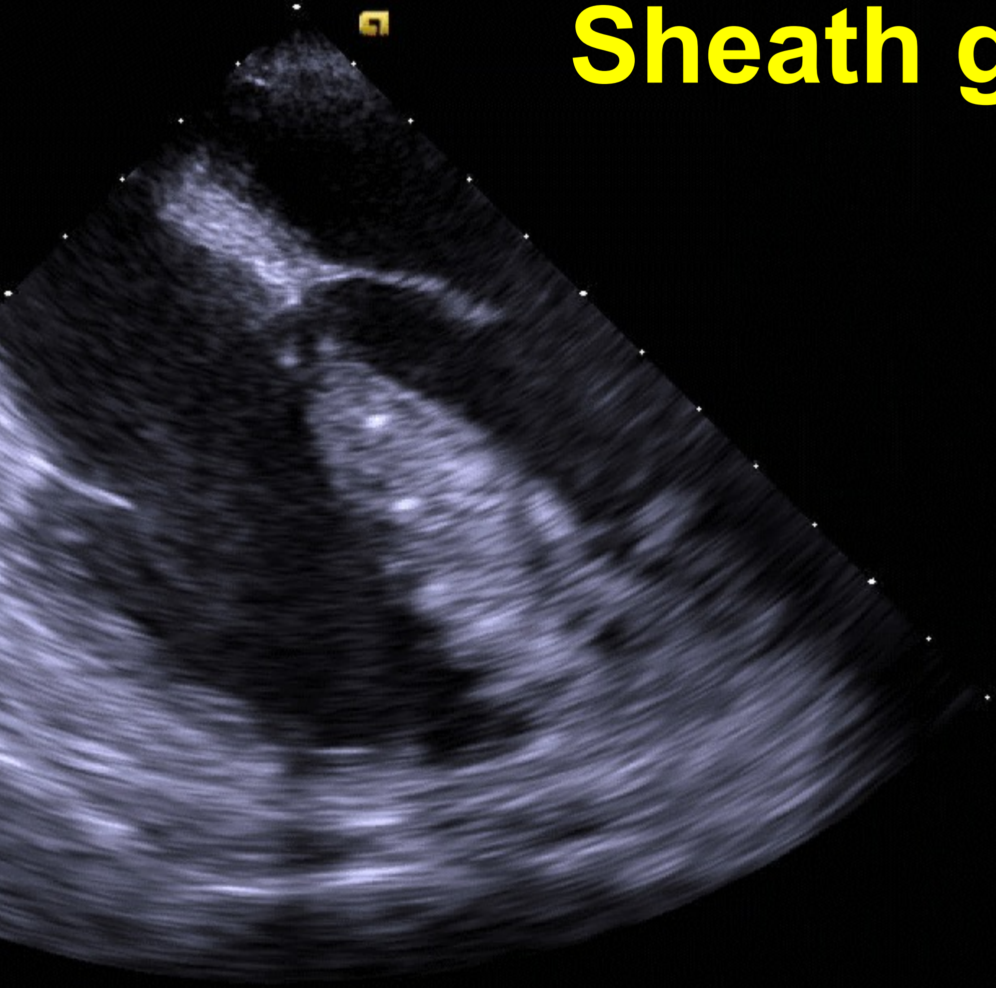

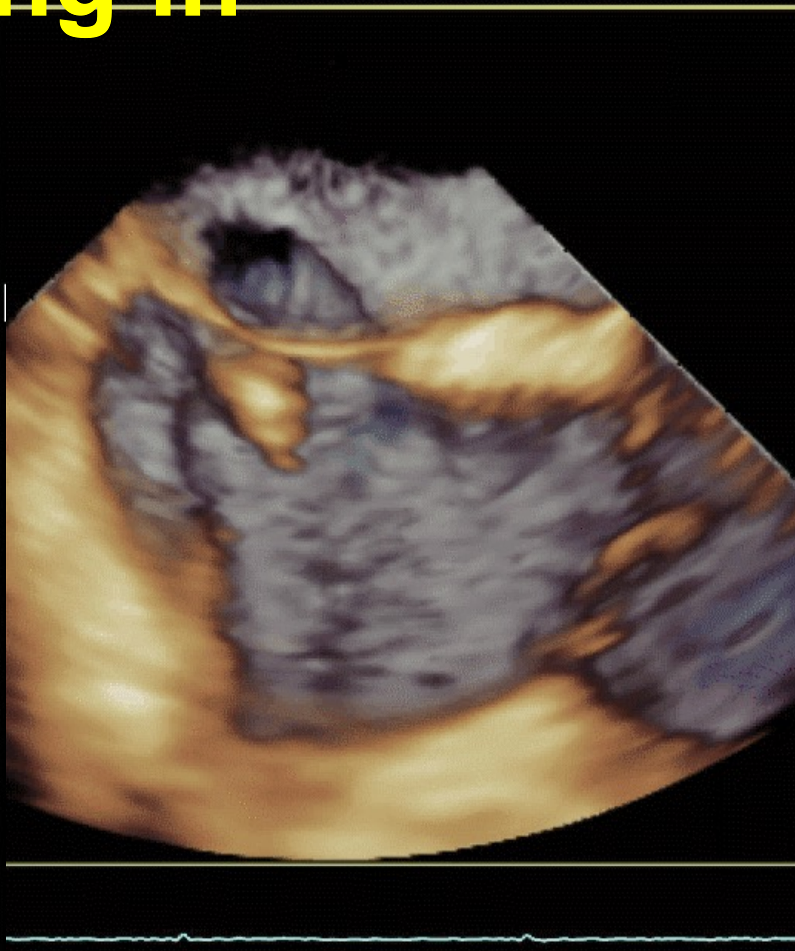

Sheath going in

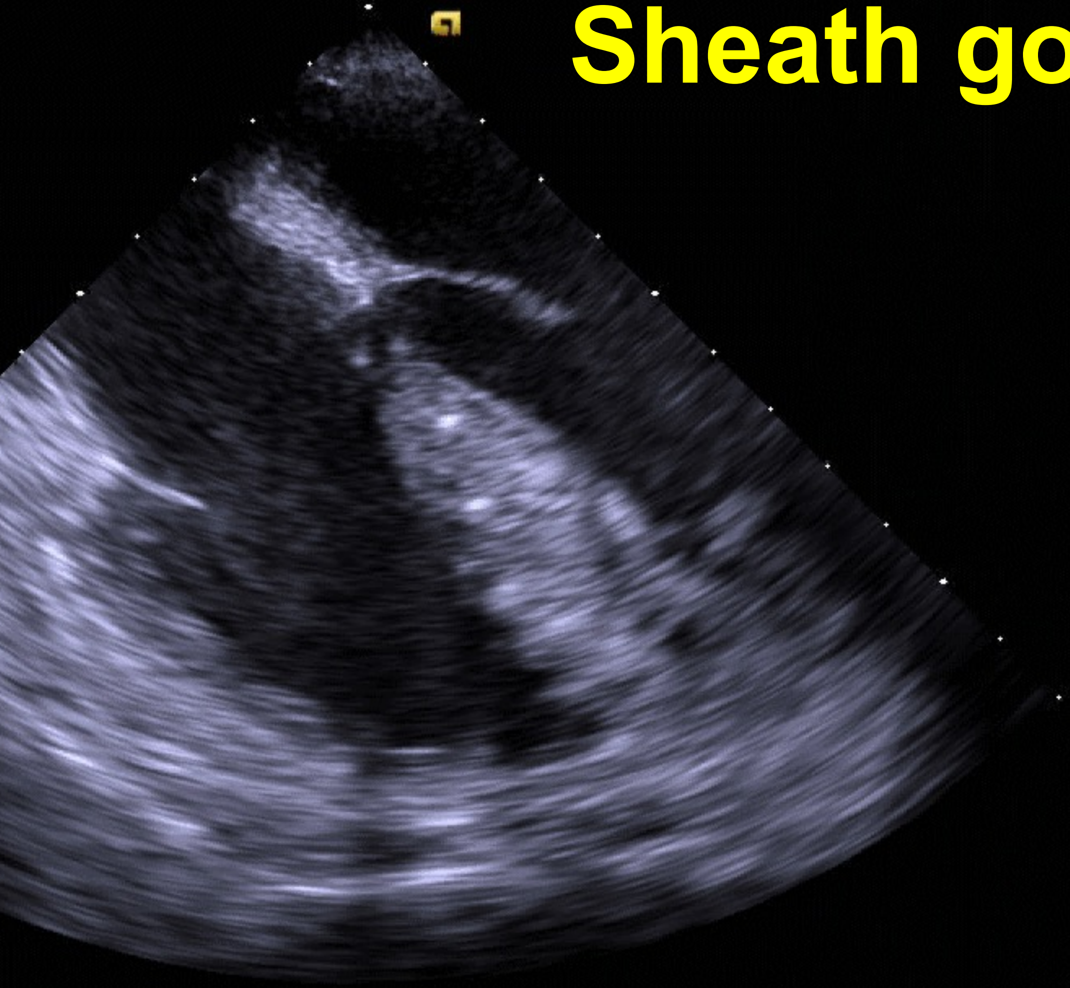

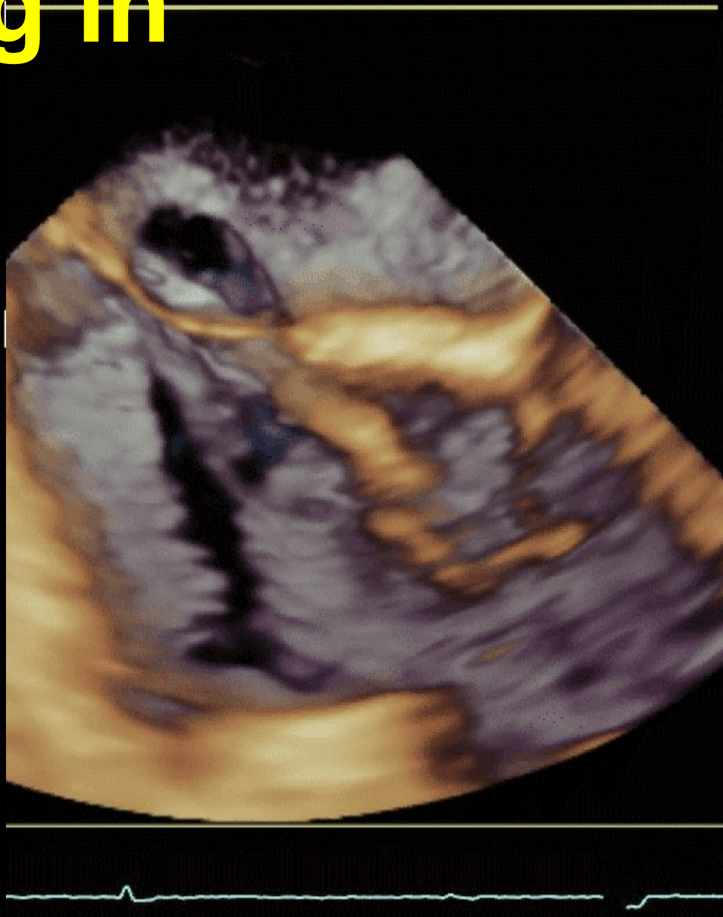

The Delivery System

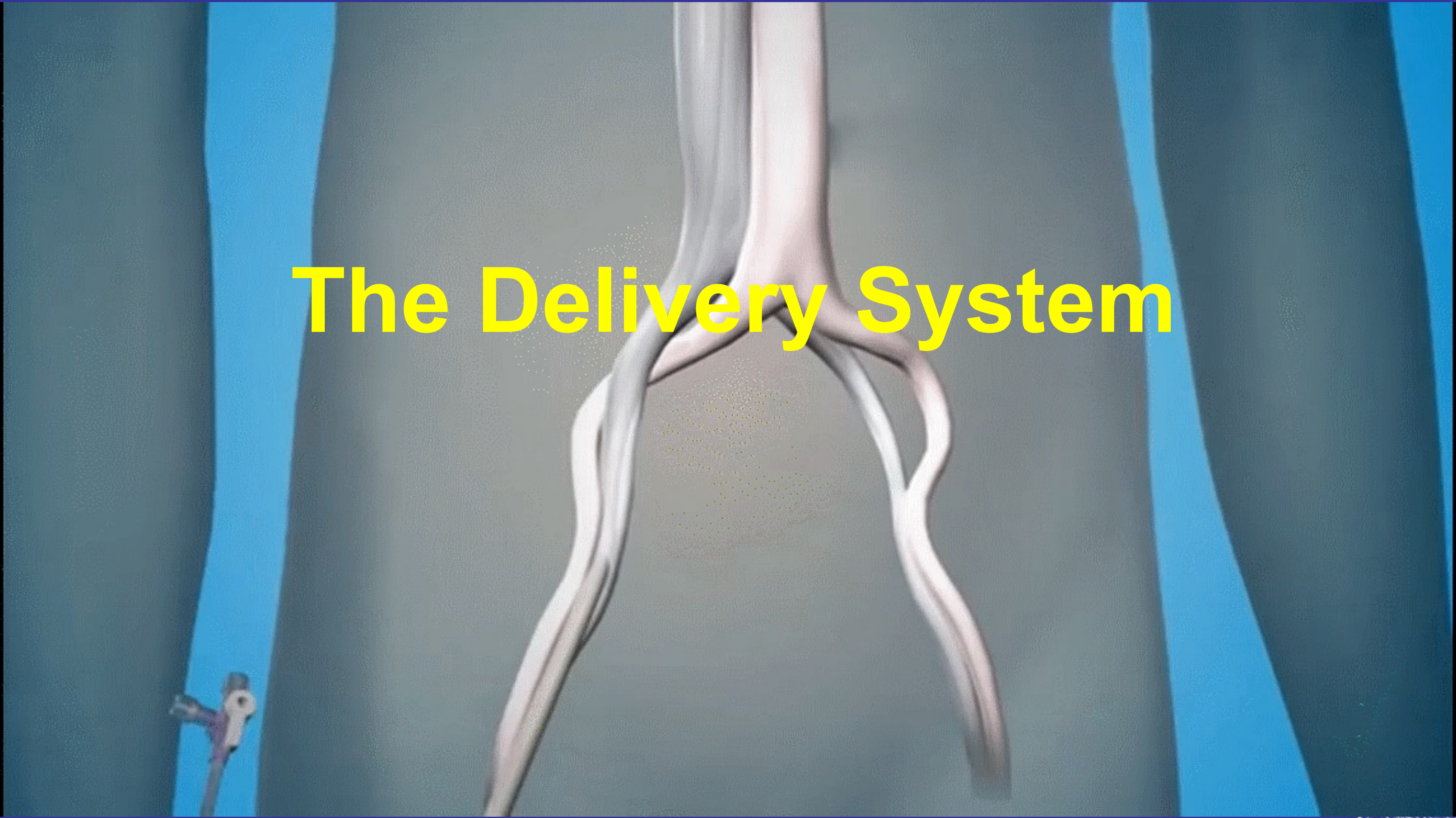

The Delivery System

- The Delivery System
  - Confirm delivery system present out of sheath
  - Monitor delivery system turning to the TV
  - Once at appropriate orientation, monitor delivery system crossing the tricuspid valve
  - Important to be aware angle of device to optimal TV entry can vary due to anatomical variability
  - Ensure delivery system is neither too posterior (directed toward the aorta) nor too anterior (directed to RV free wall).

Delivery System Turning to TV

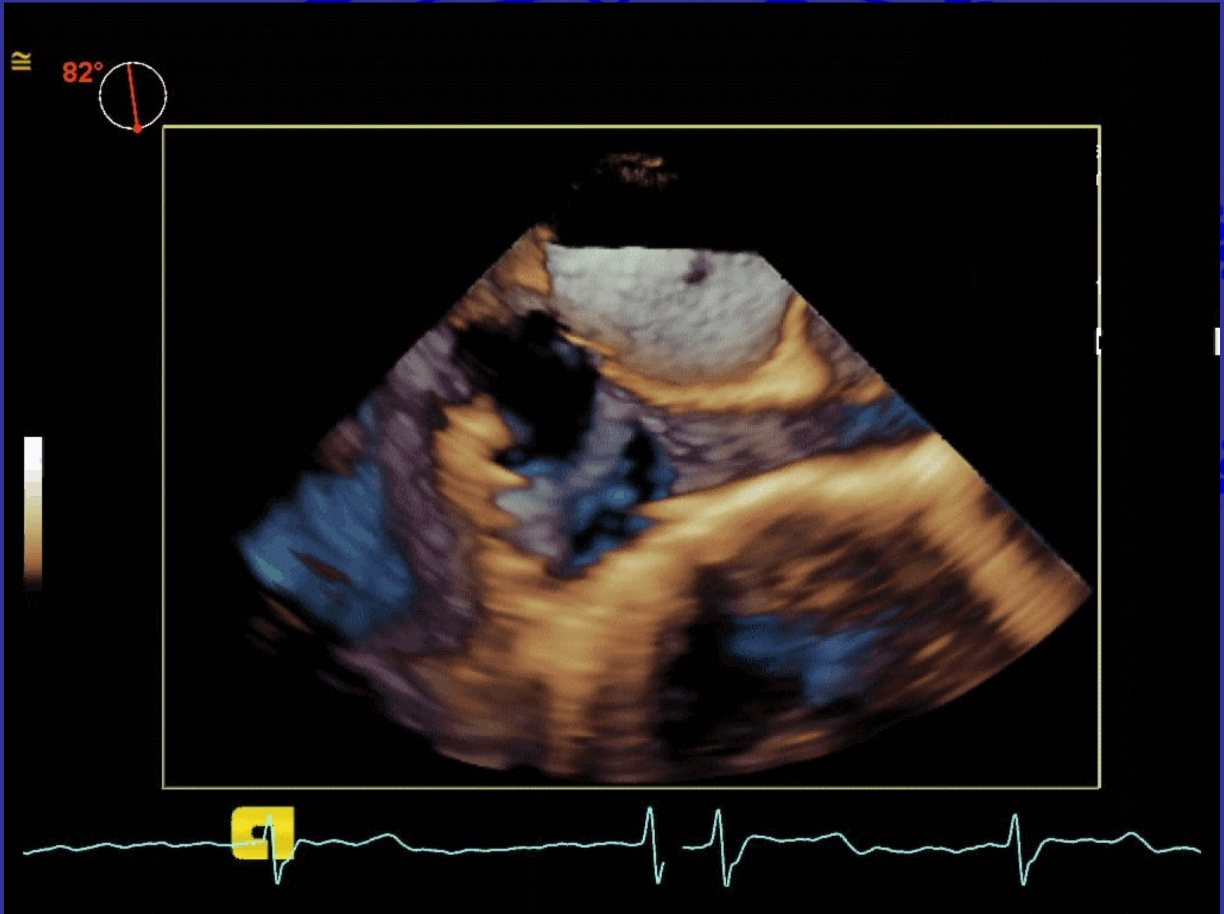

Delivery System Turning to TV

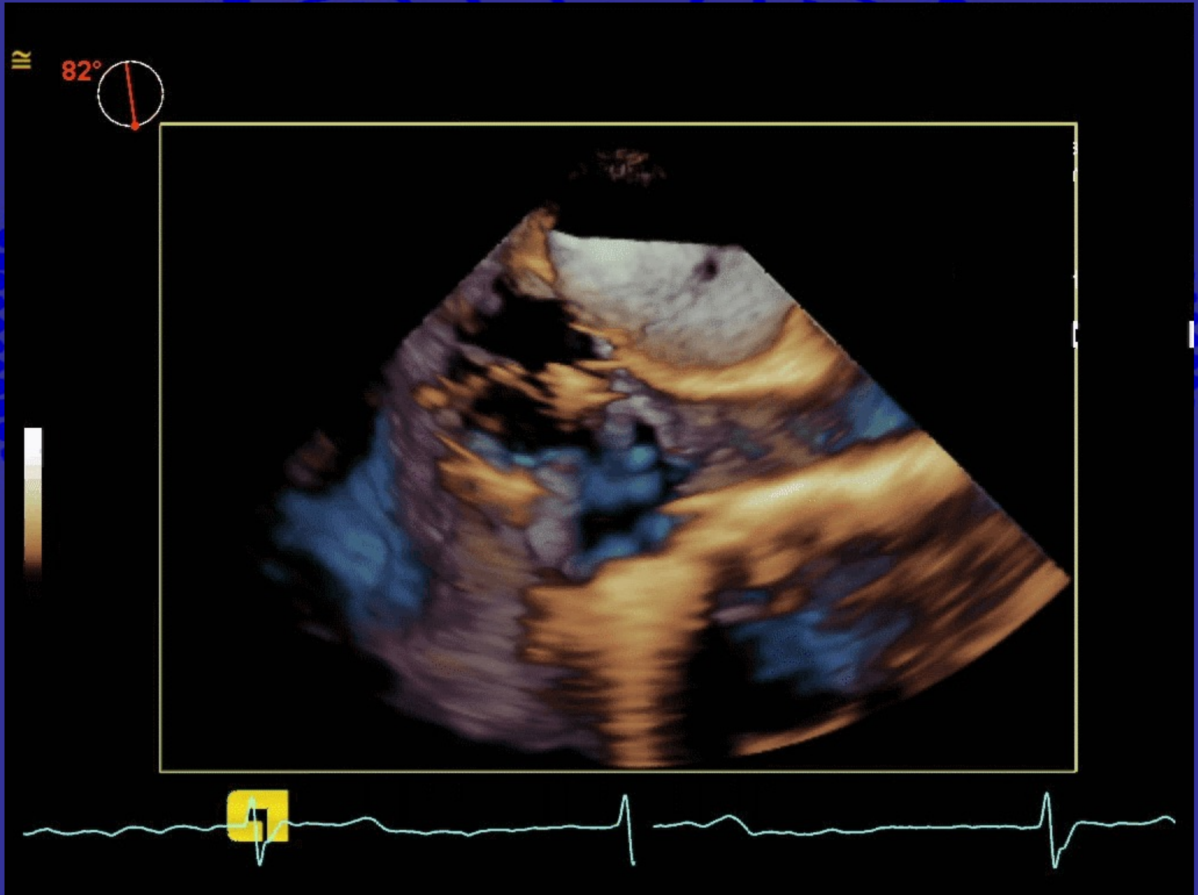

Delivery System Crossing the Tricuspid Valve

Delivery System Turning to TV

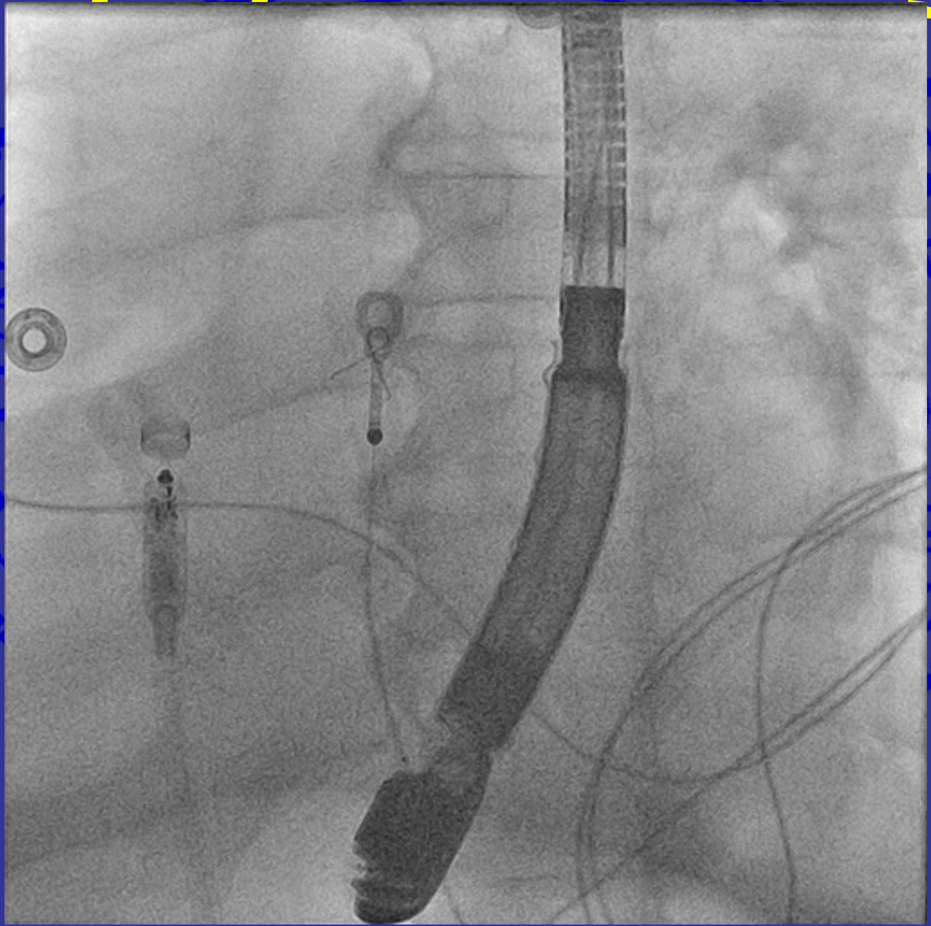

Delivery System Crossing the TV 2D

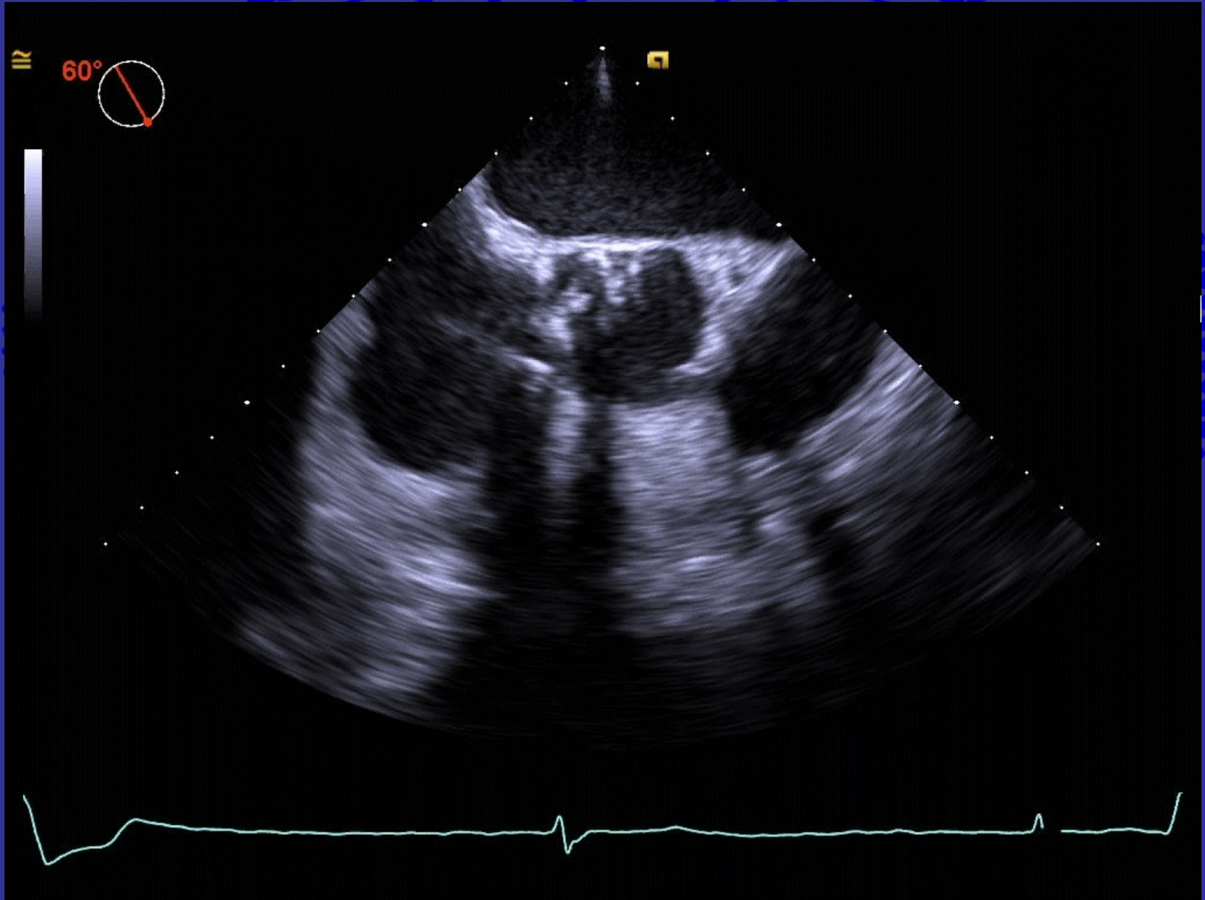

Delivery System Crossing the TV 2D

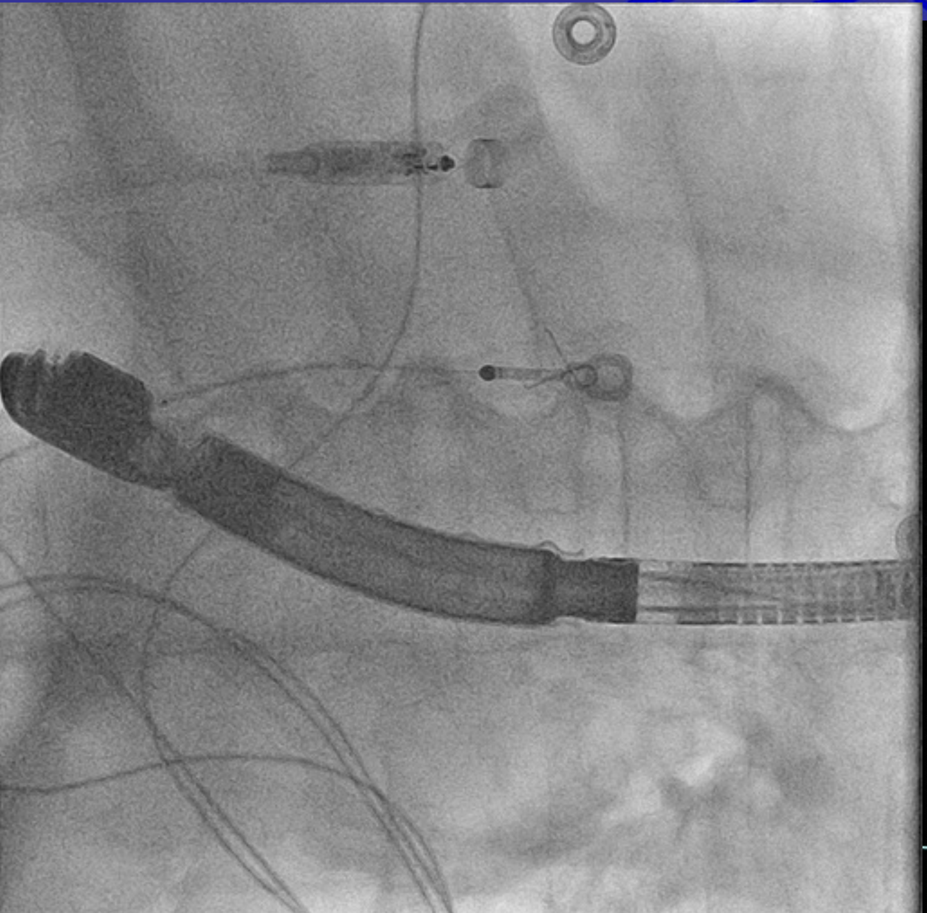

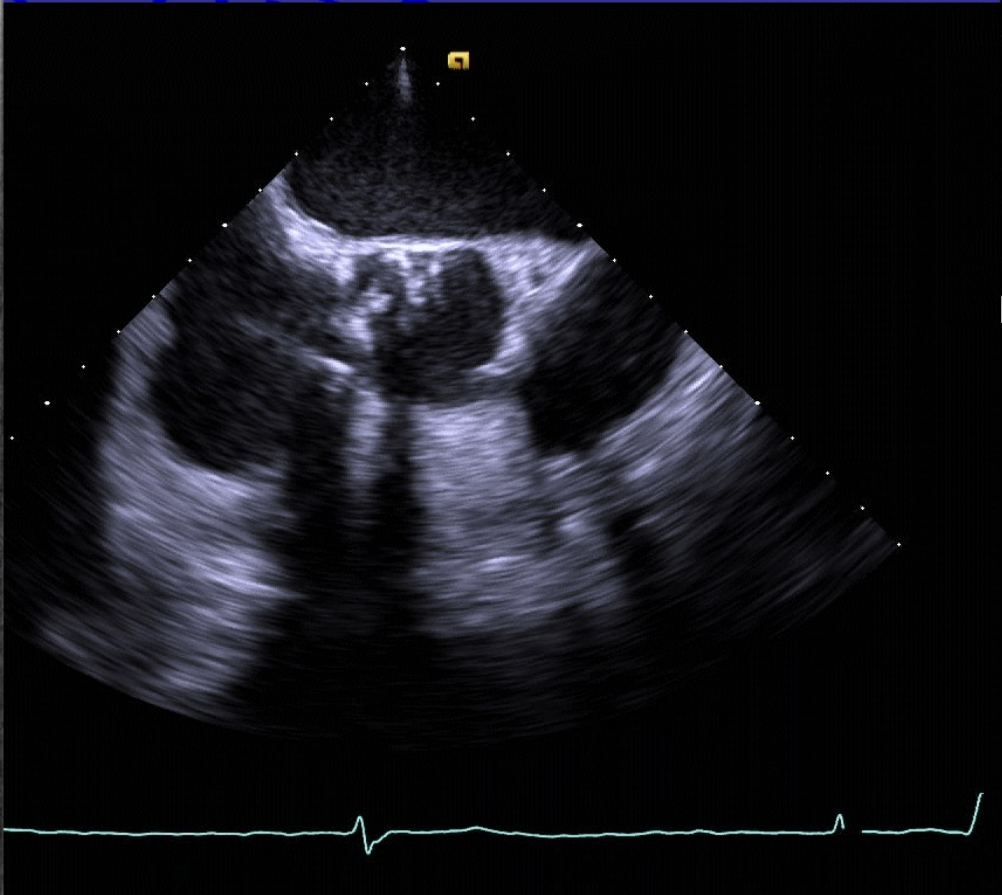

Delivery System Crossing the TV

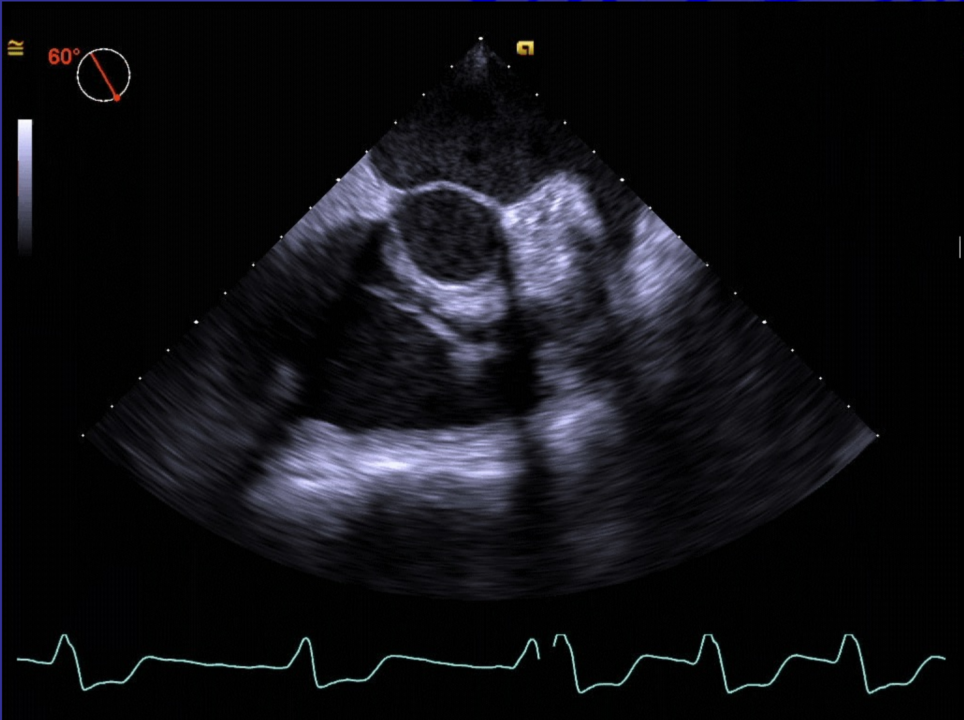

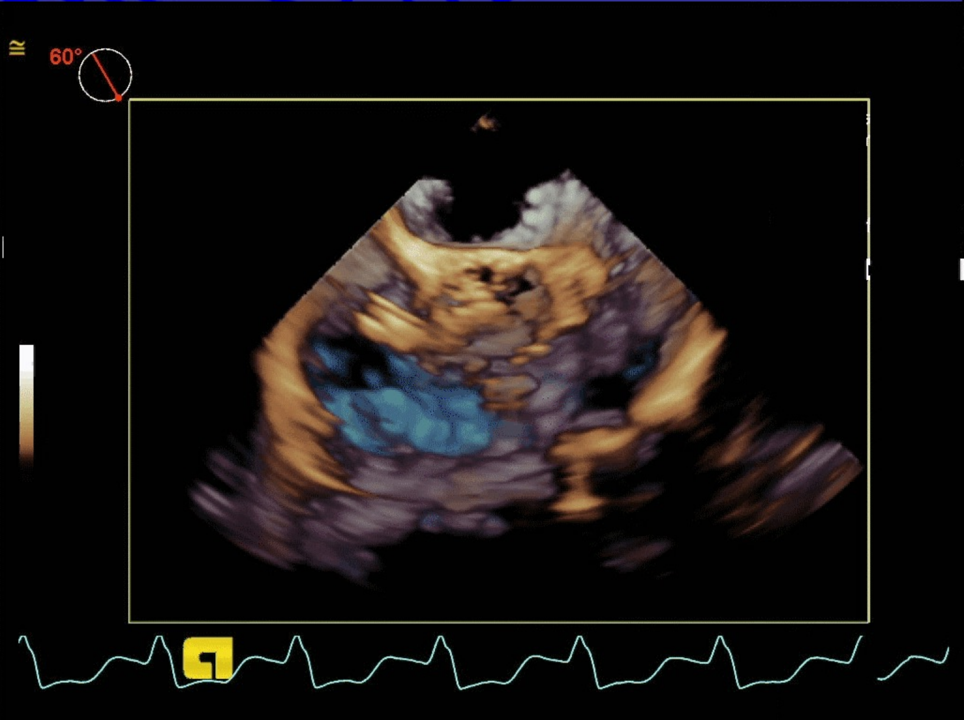

Delivery System Crossing the TV 2D
Another case

Delivery System Crossing the TV 2D

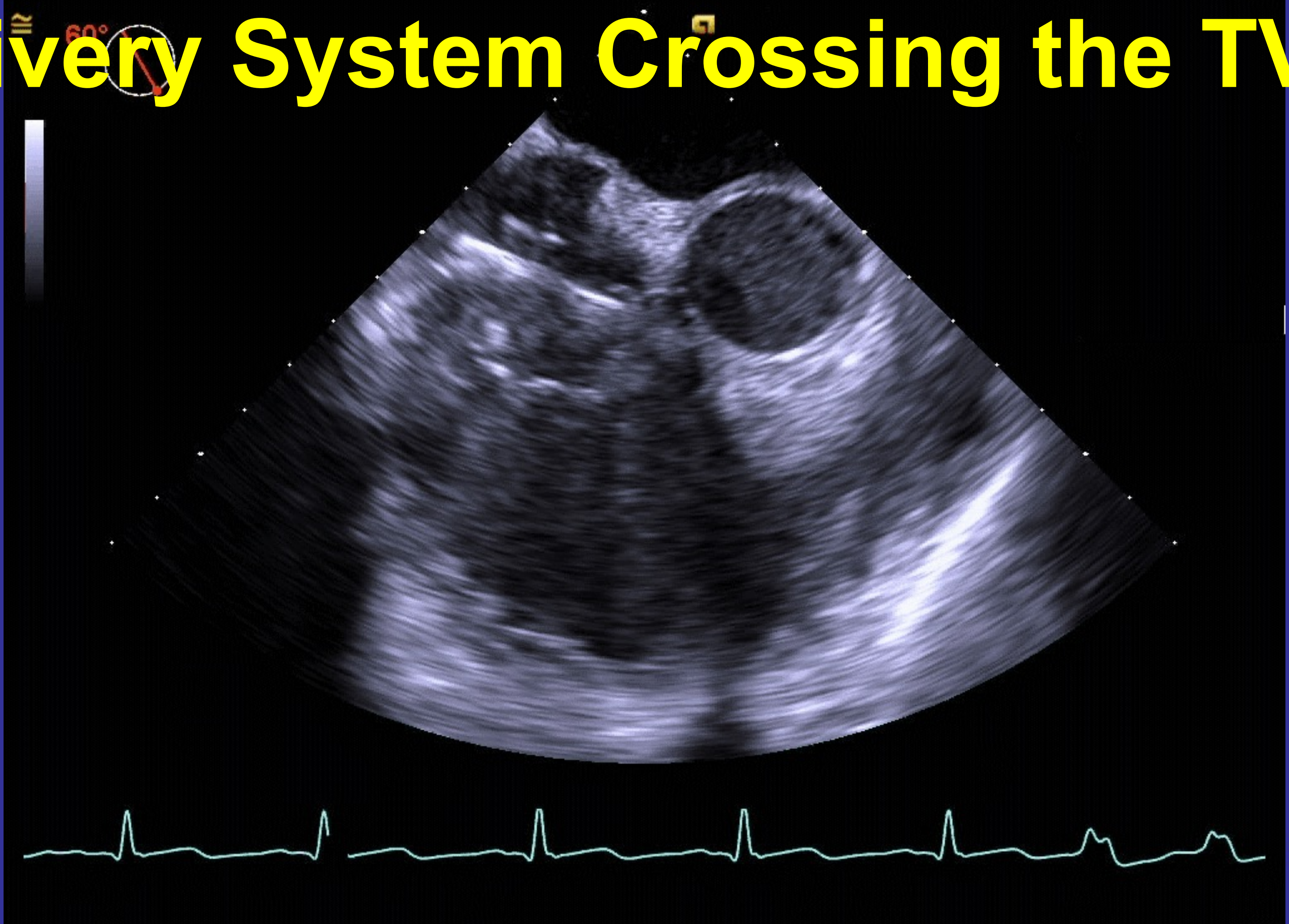

Delivery System Crossing the TV 2D

Micra is too anterior – pulling back

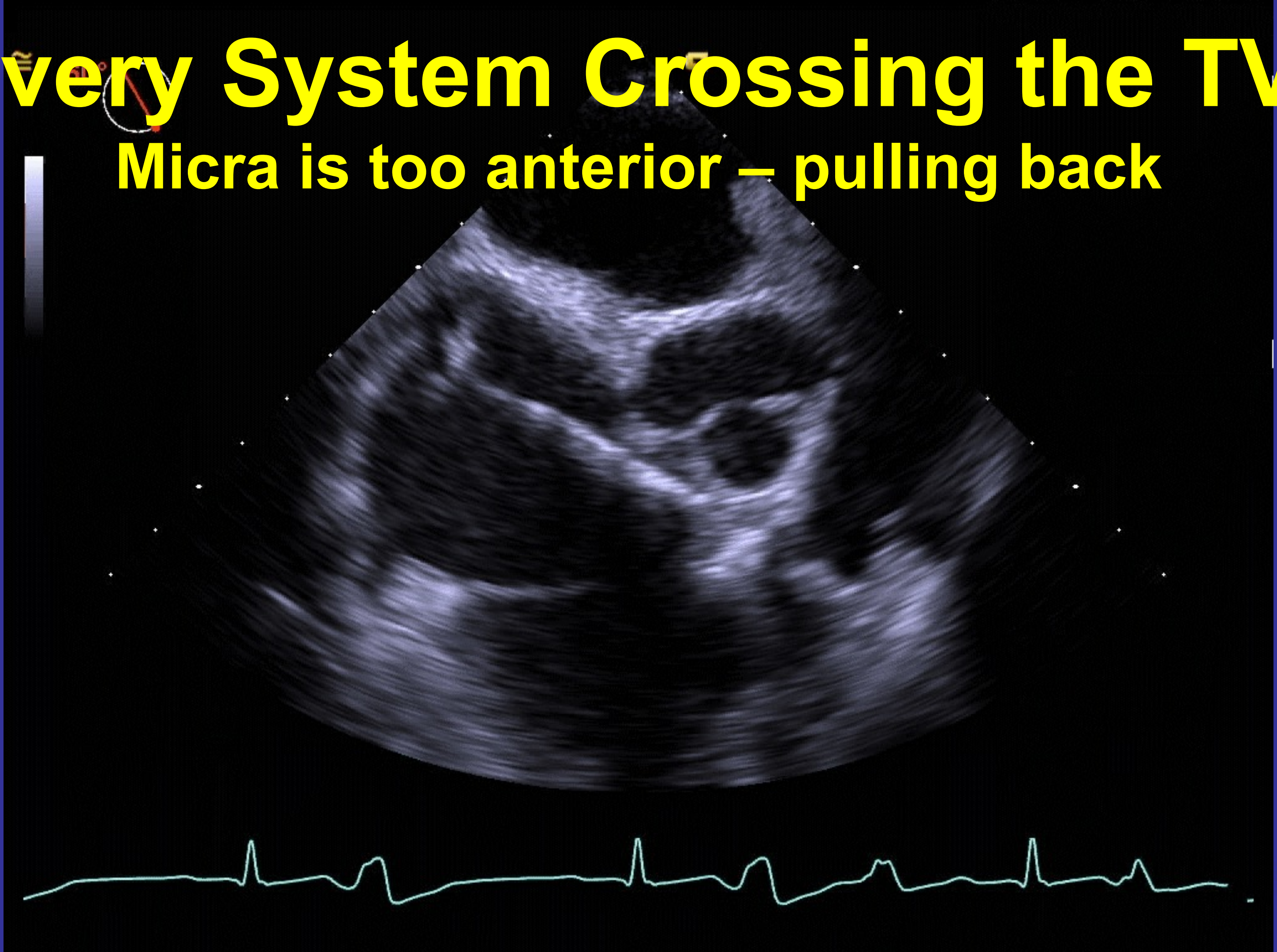

Delivery System Crossing the TV 2D

Micra is well-positioned at basal septum after pull back

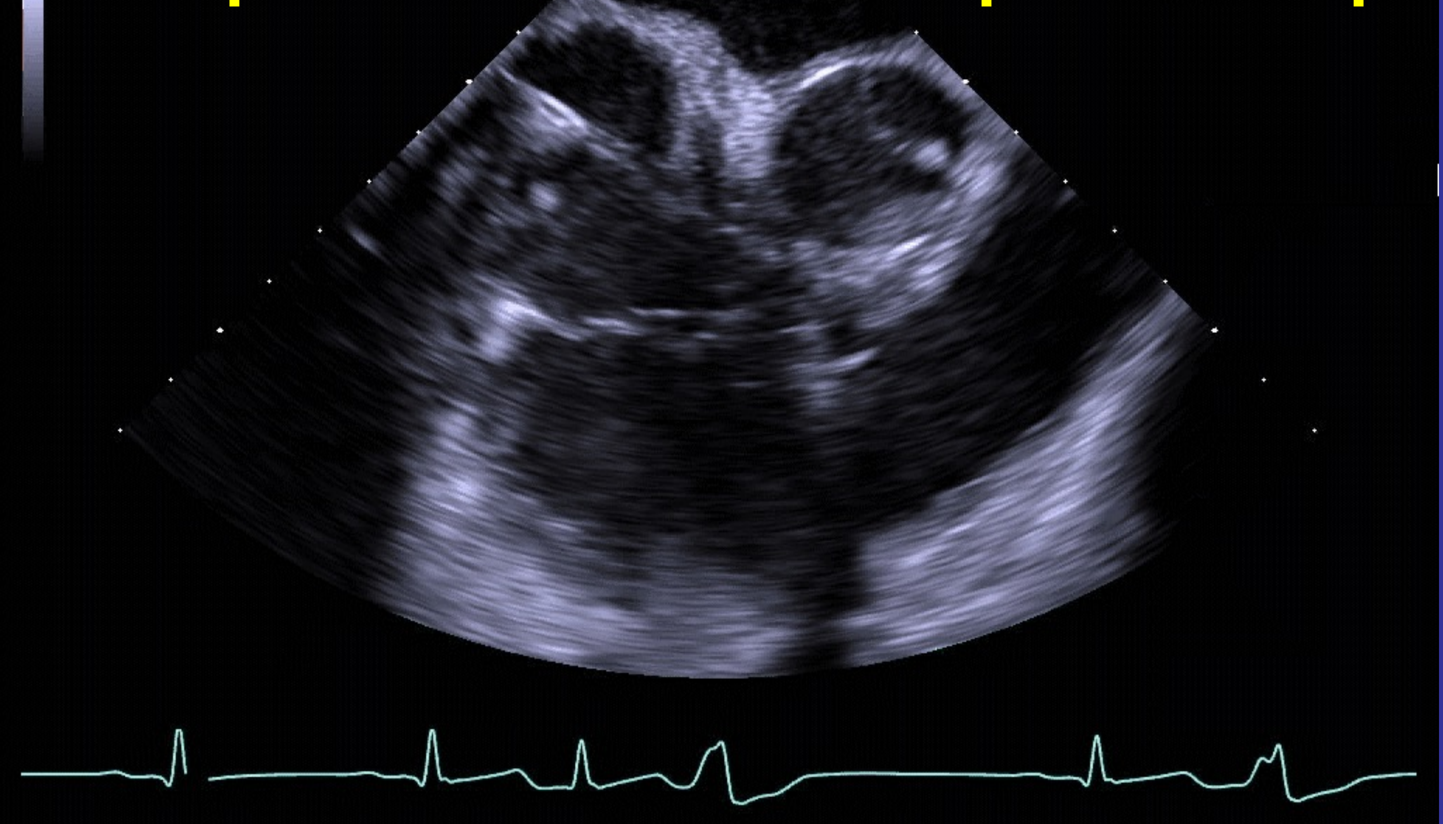

Before Delivery, Optimize the anchor location

Pre-Delivery

- Pre-Delivery: Anchor Location Optimization
  - Avoiding the anchor from being too basal and apical
  - Trans-gastric view: biplane long and short axis views of the RV
  - Trans-gastric view (remaining in biplane) to monitor device engaging the septum
  - Observe for Goose-necking
  - Dye injection using fluoroscopy in AP/RAO and LAO views to fluoroscopically confirm deployment location.
  - Mid-esophageal Short Axis View: Optimal view of tricuspid valve, device, and pulmonary valve all in same view. Biplane can additionally assist to optimize image.
  - Mid-esophageal 4-Chamber View (RV focused): Visualize RV, septum, device, and tricuspid valve.

Gastric View Biplane, Engaging the Septum

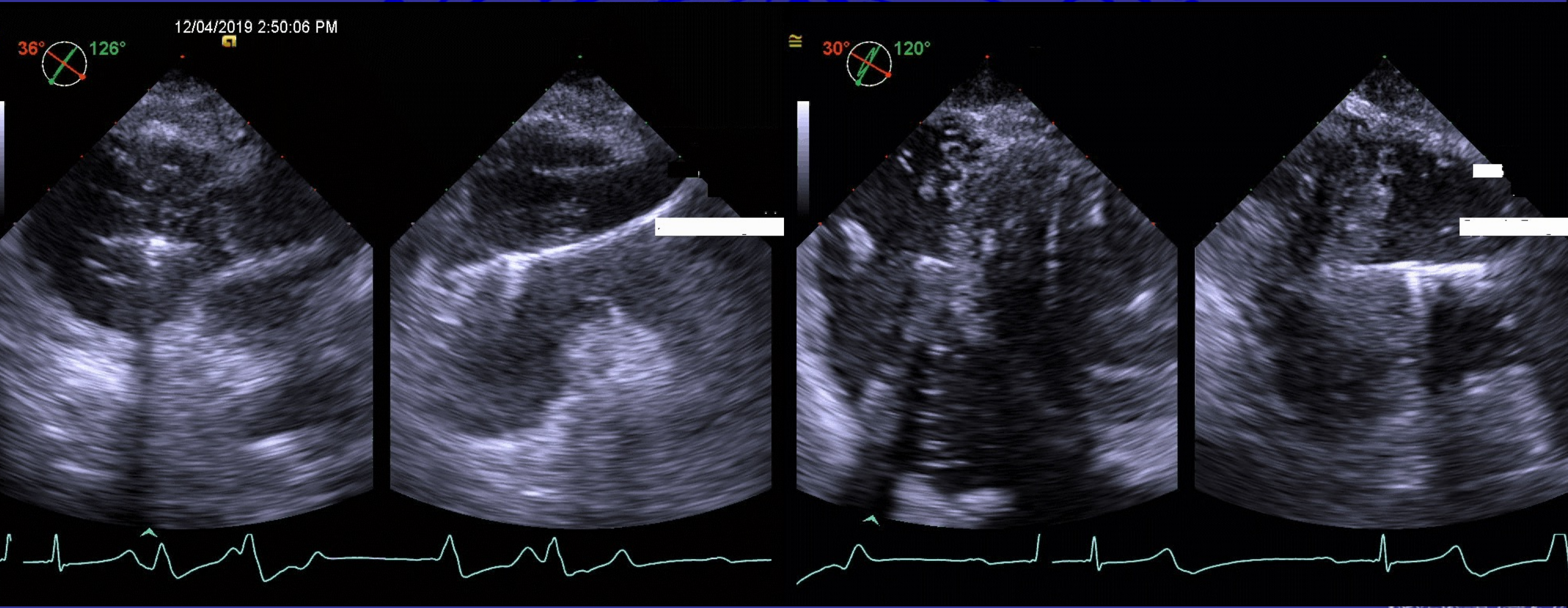

Too Basal

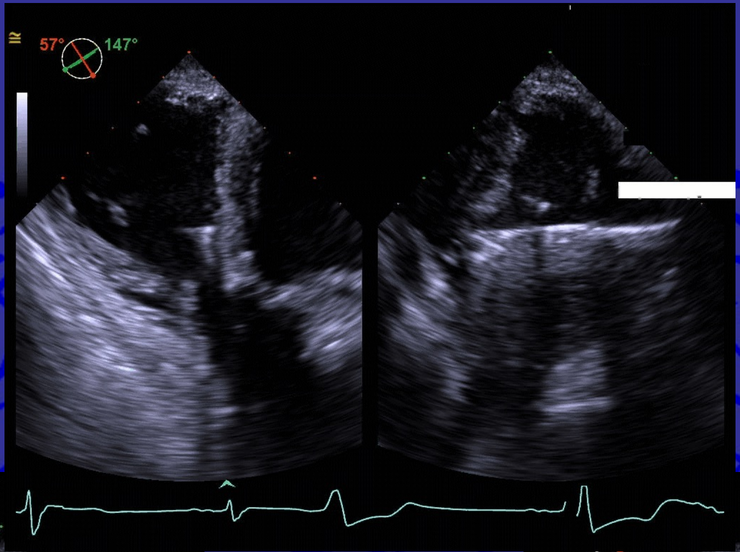

Too Apical

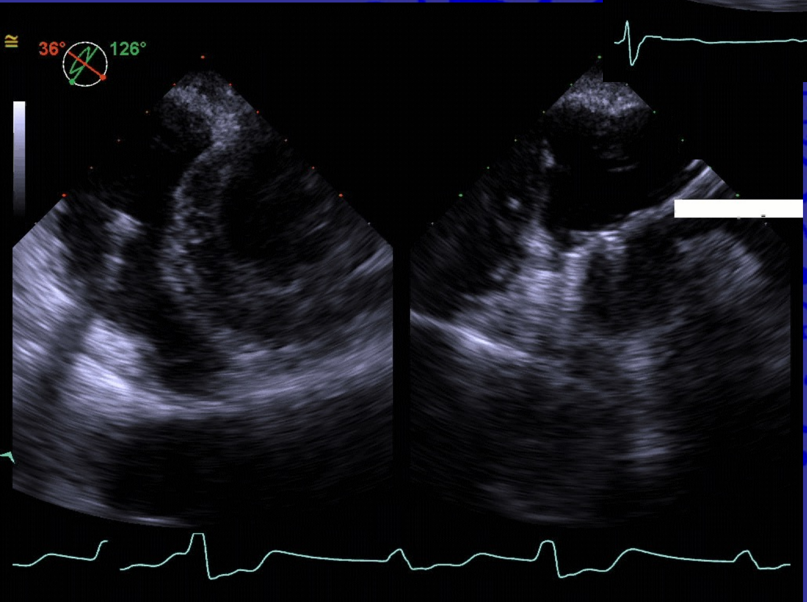

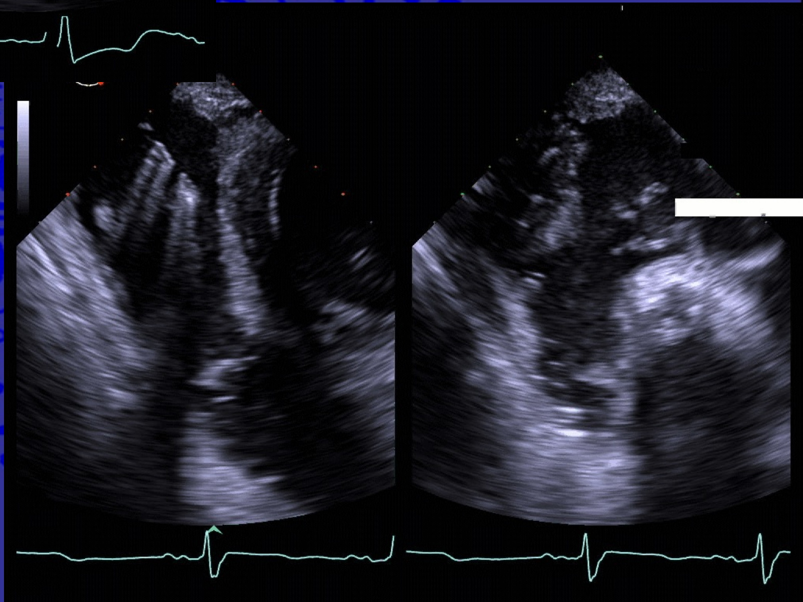

Gastric View Biplane, Engaging the Septum

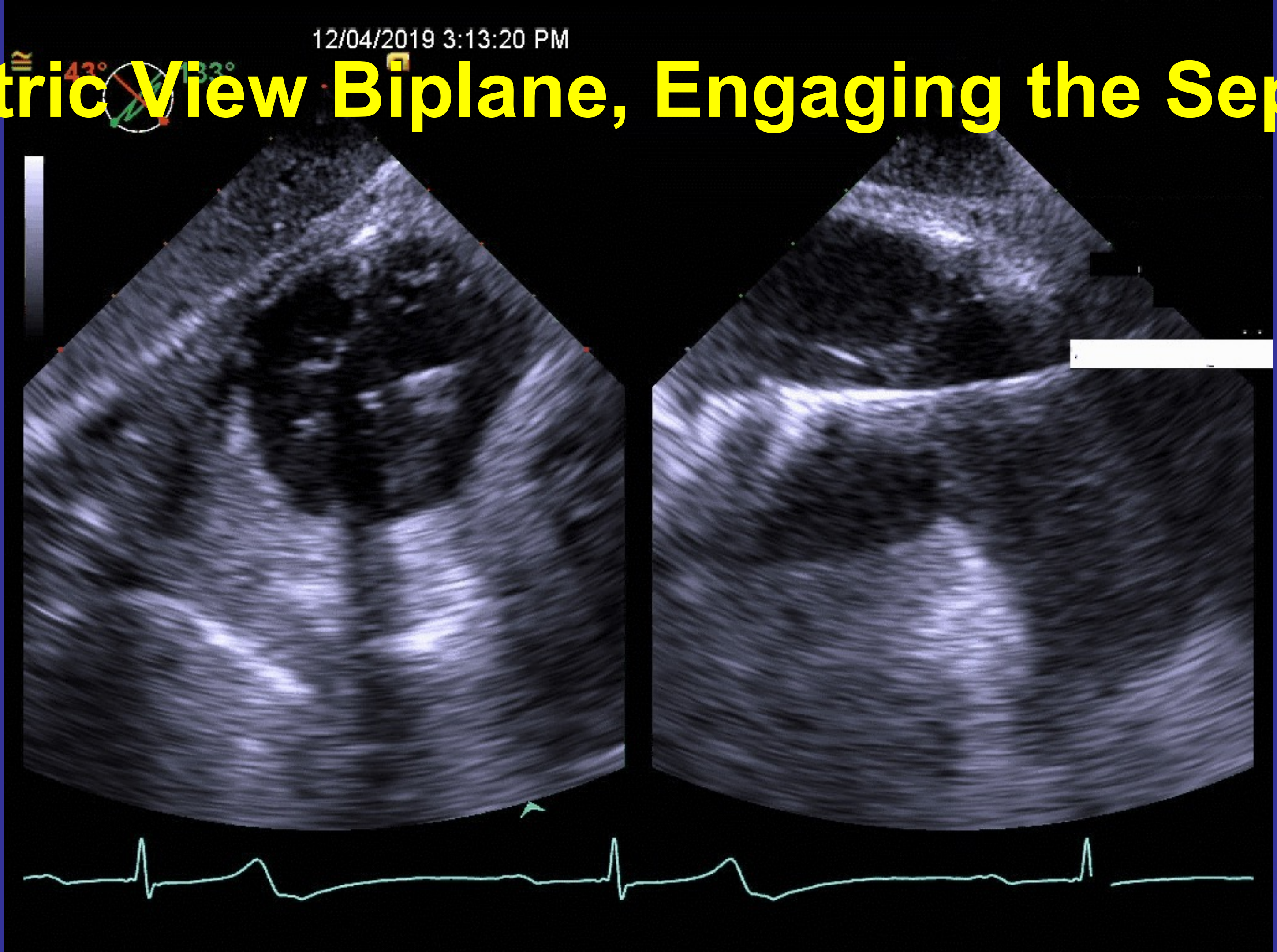

Gastric View Biplane, Goose-necking

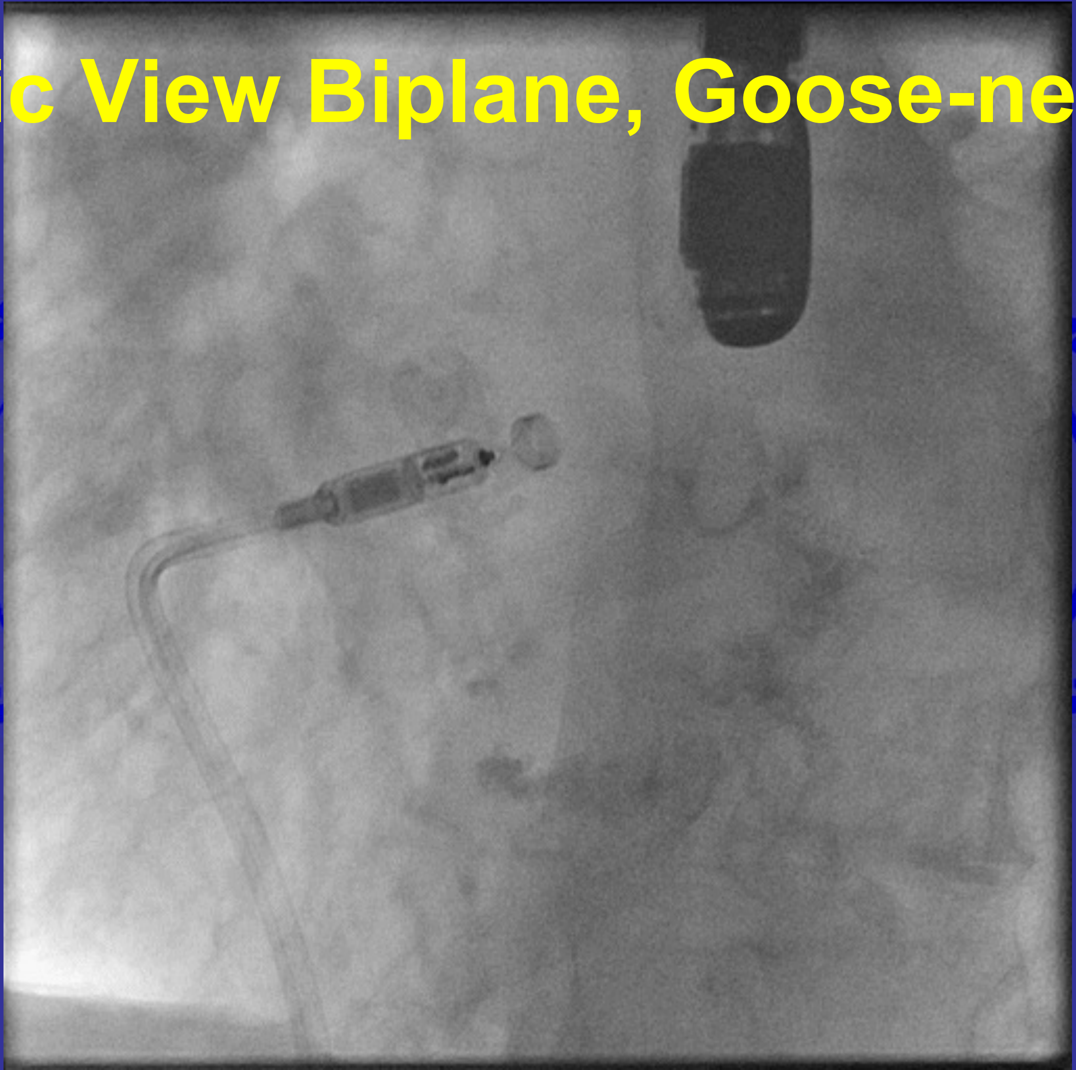

Gastric View Biplane, Goose-necking

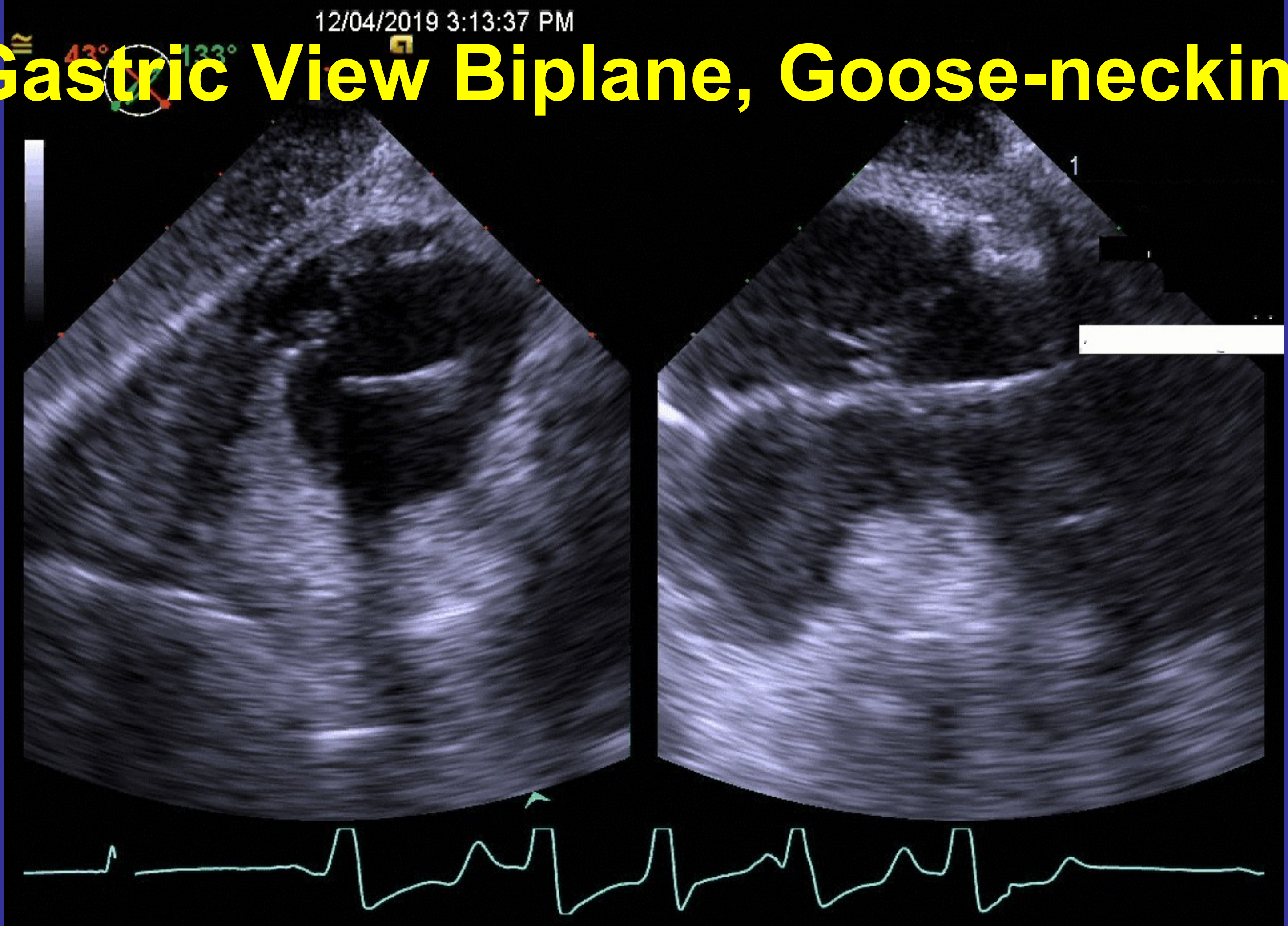

Gastric View Biplane, Goose-necking

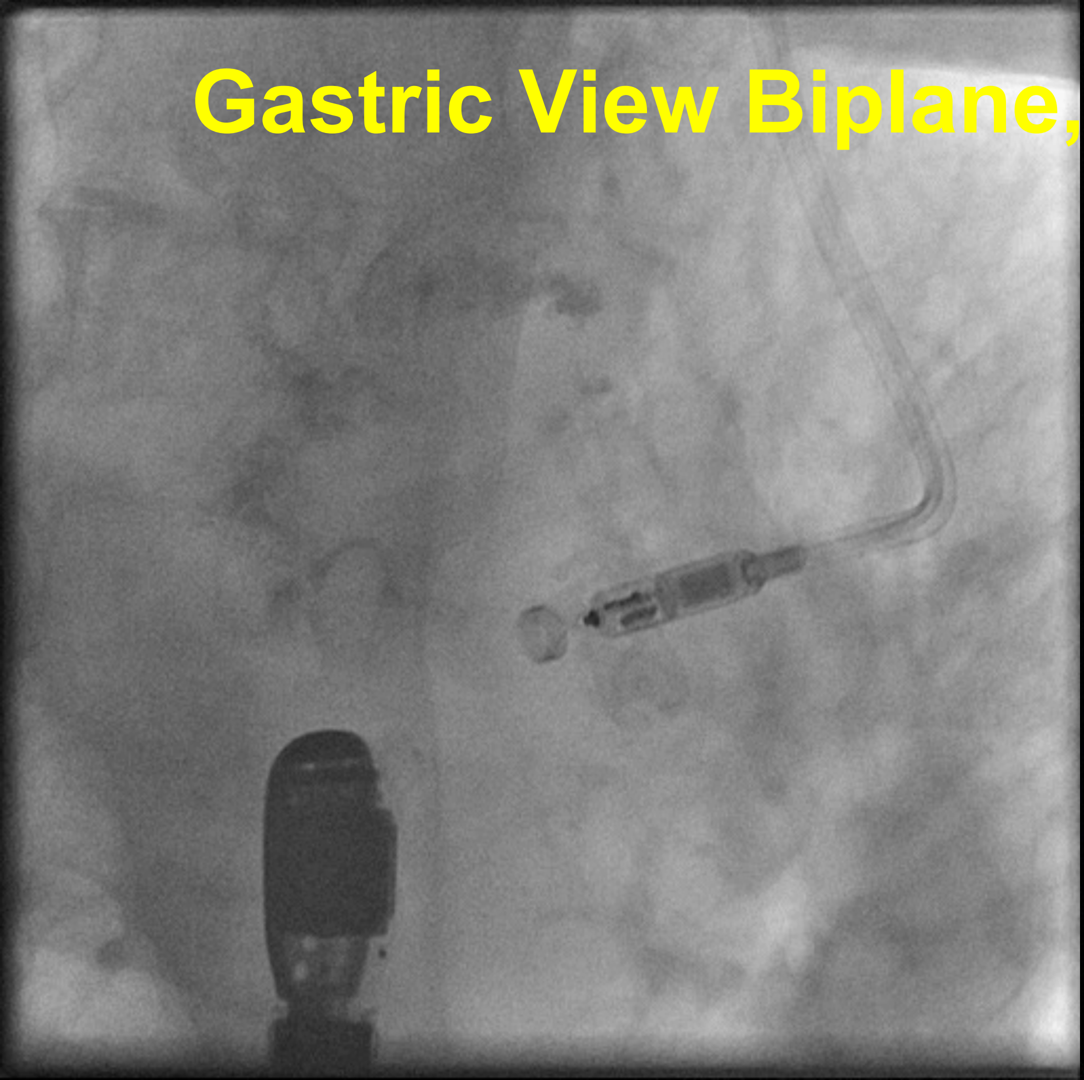

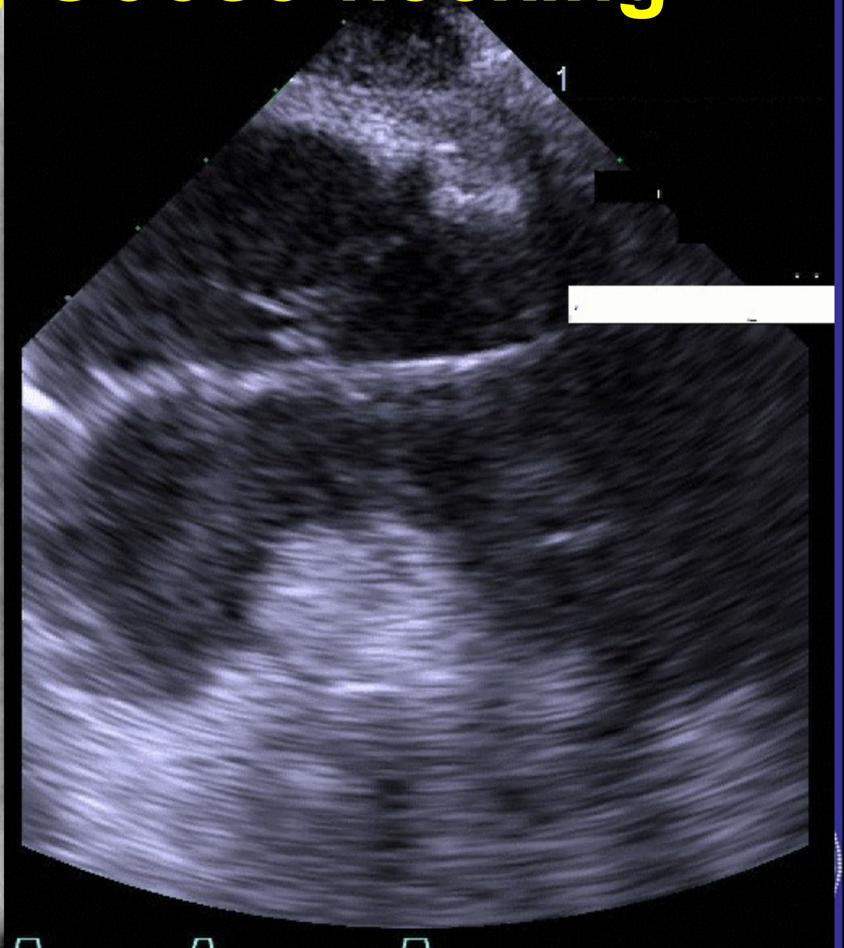

Dye injection

Dye injection: AP/RAO

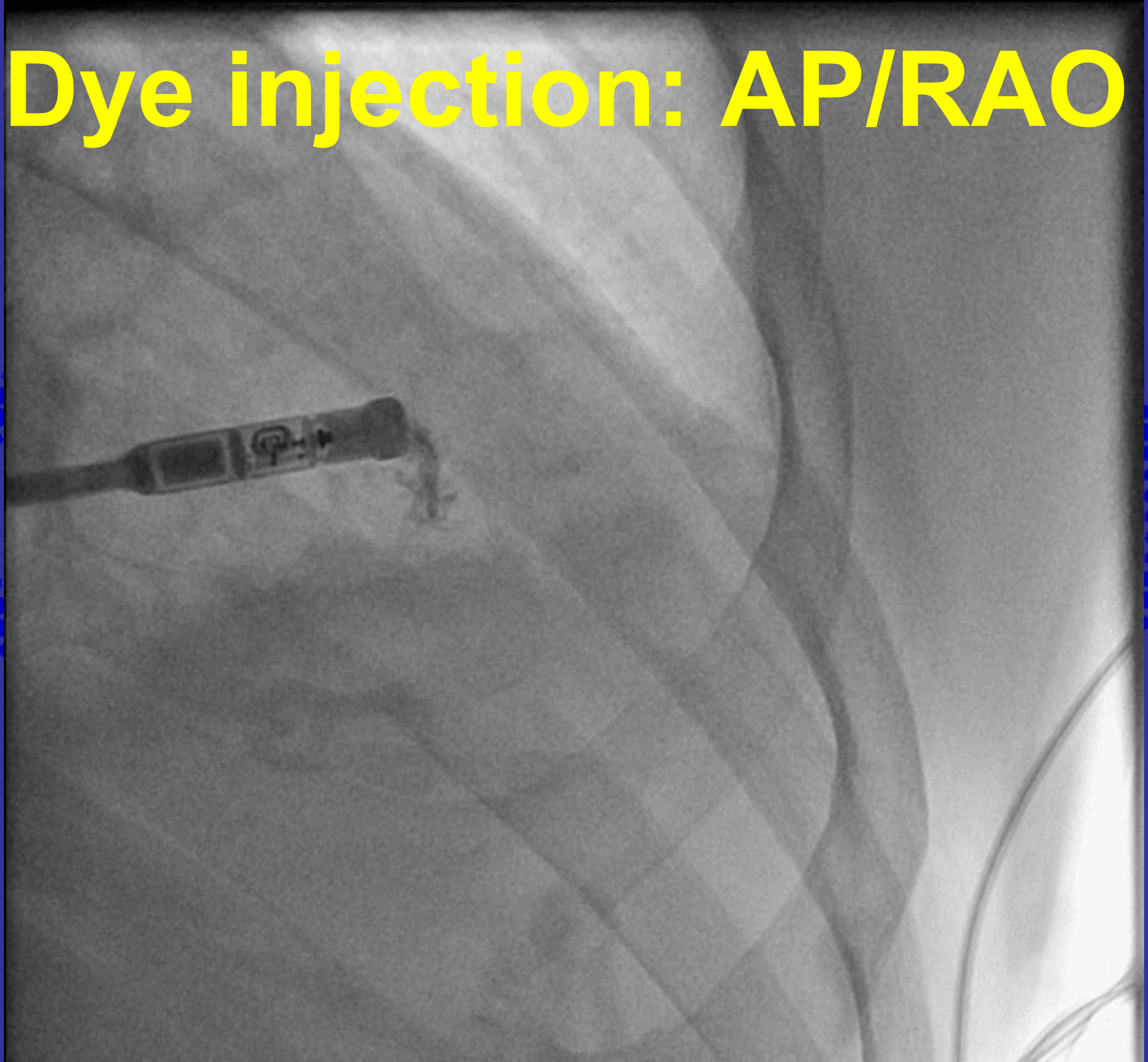

Dye injection: LAO

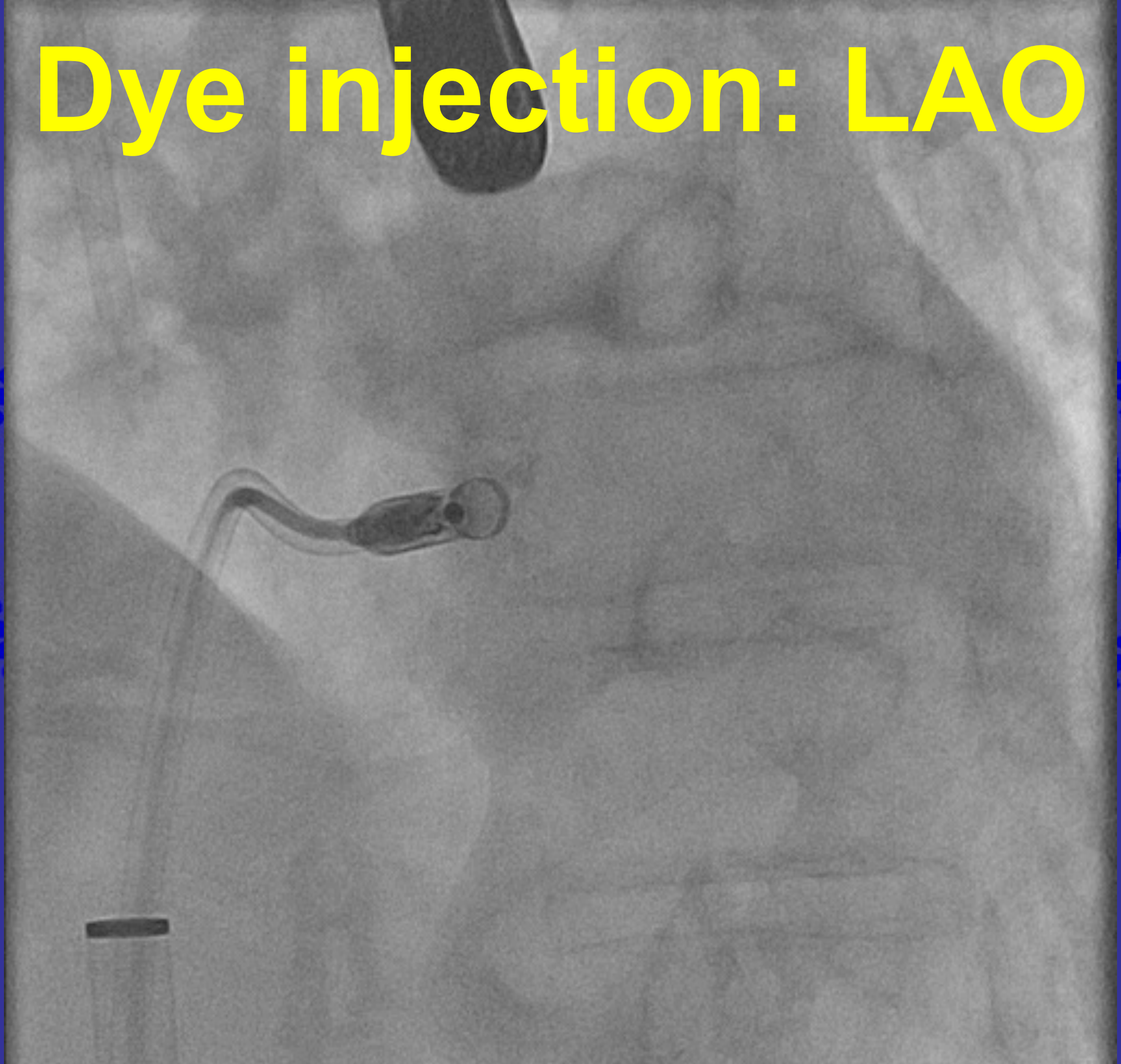

Deployment

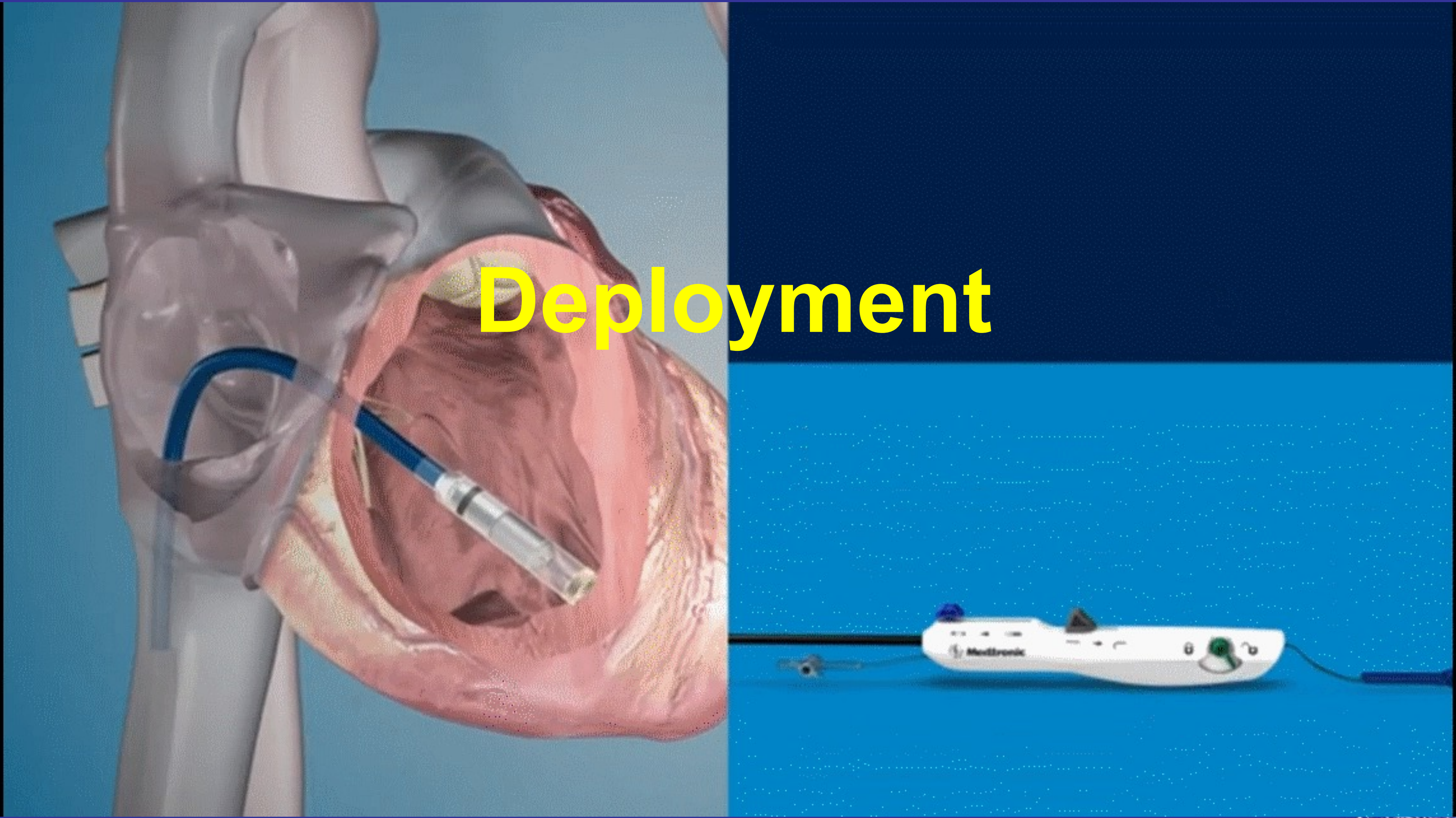

Deployment

- Deployment
  - Remain in trans-gastric biplane, observe micra position and deployment and observed goose neck, tether remains attached
  - Device then released half-way, remain in goose neck. If unable to perform trans-gastric then advise mid-esophageal view of tricuspid valve, RV, and pulmonary valve with device in view.
  - Device released, tether remains attached. Consider panning/sweeping through biplane image to optimize view of device, septum, and tether. Septum and tether may come in/out of plane
  - Watch deliver system move from RV to RA attempting to continue observation of tether and device

Micra Release: success

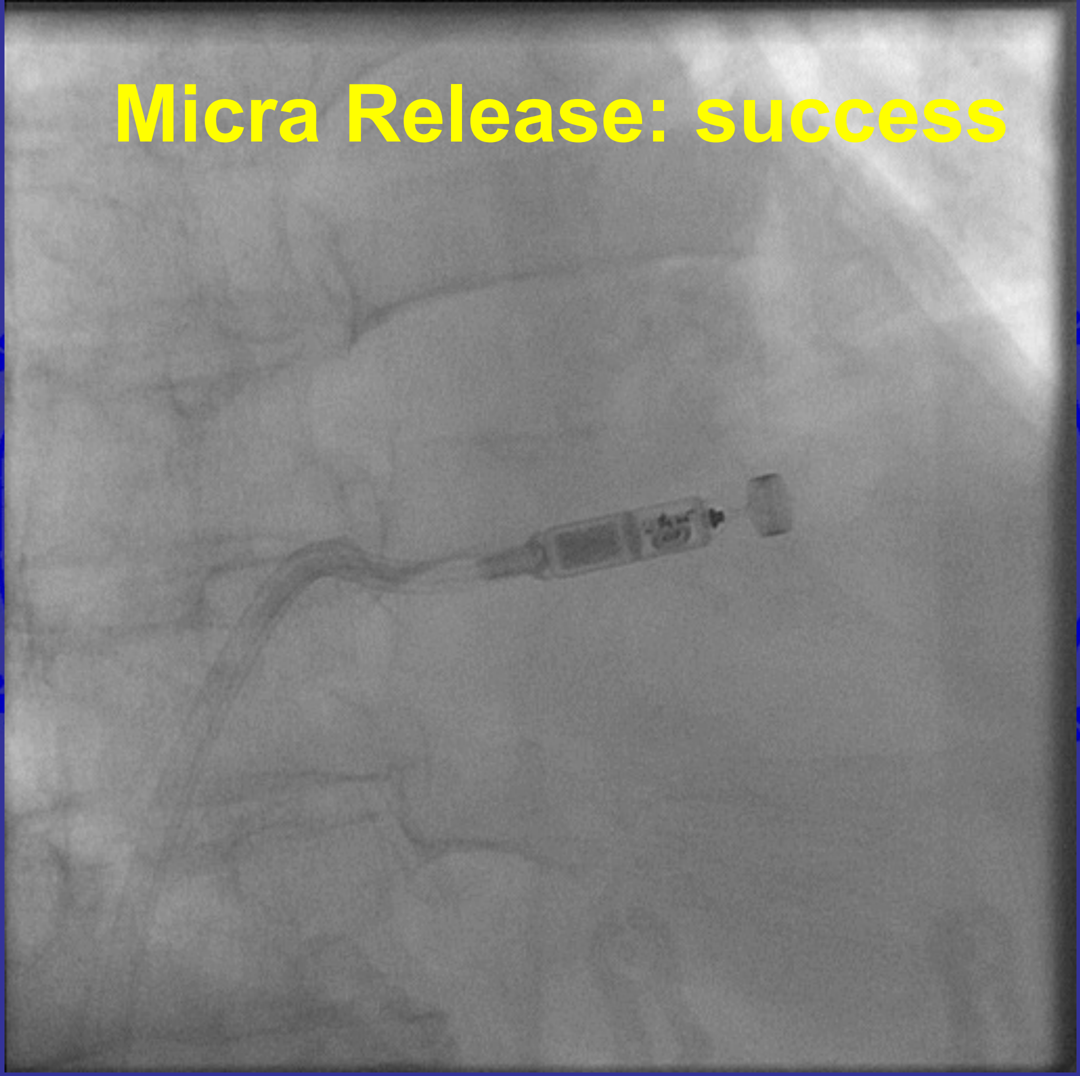

Micra deployed and Goose neck

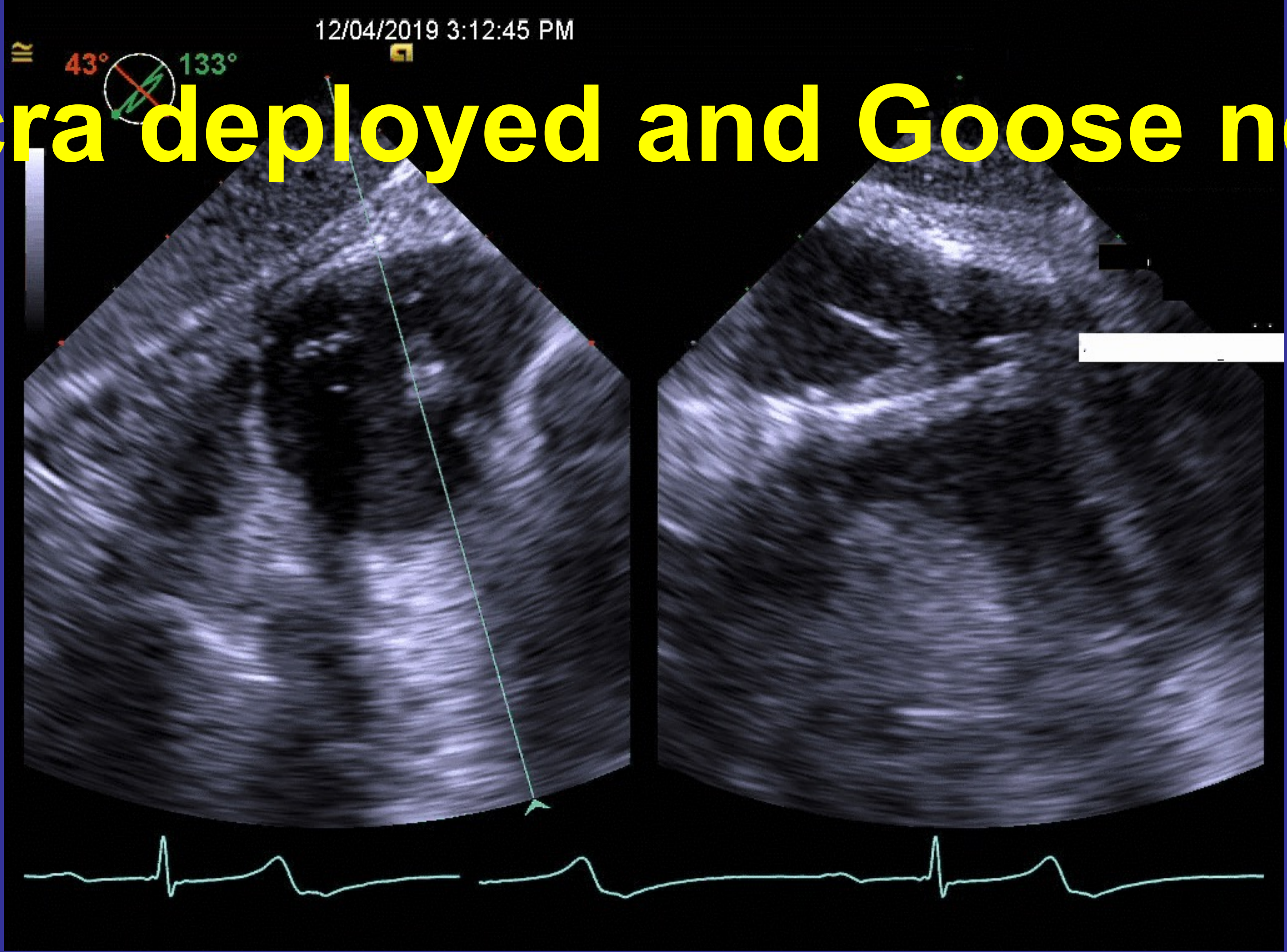

Micra deployment - replay

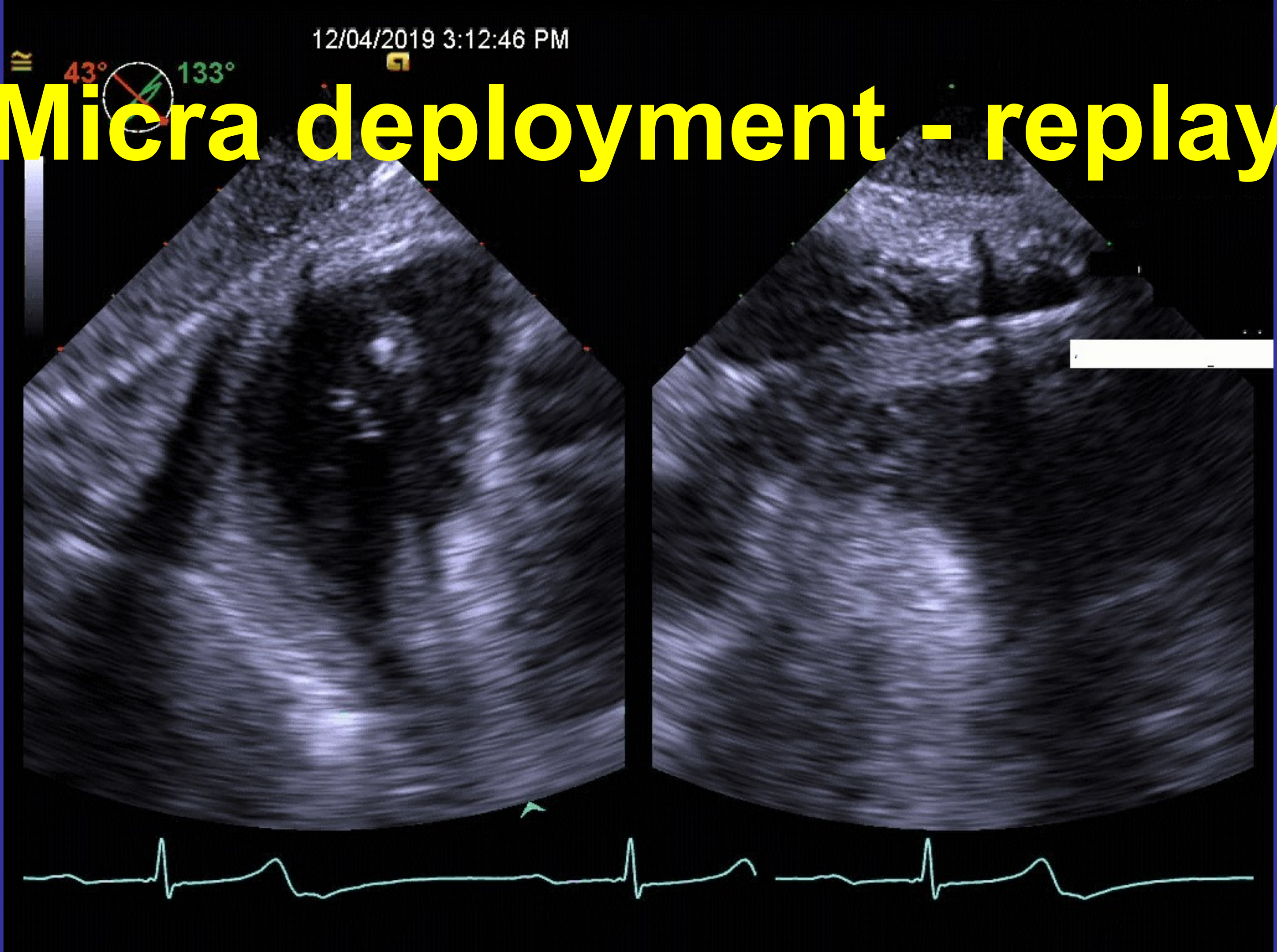

Micra deployment - demo

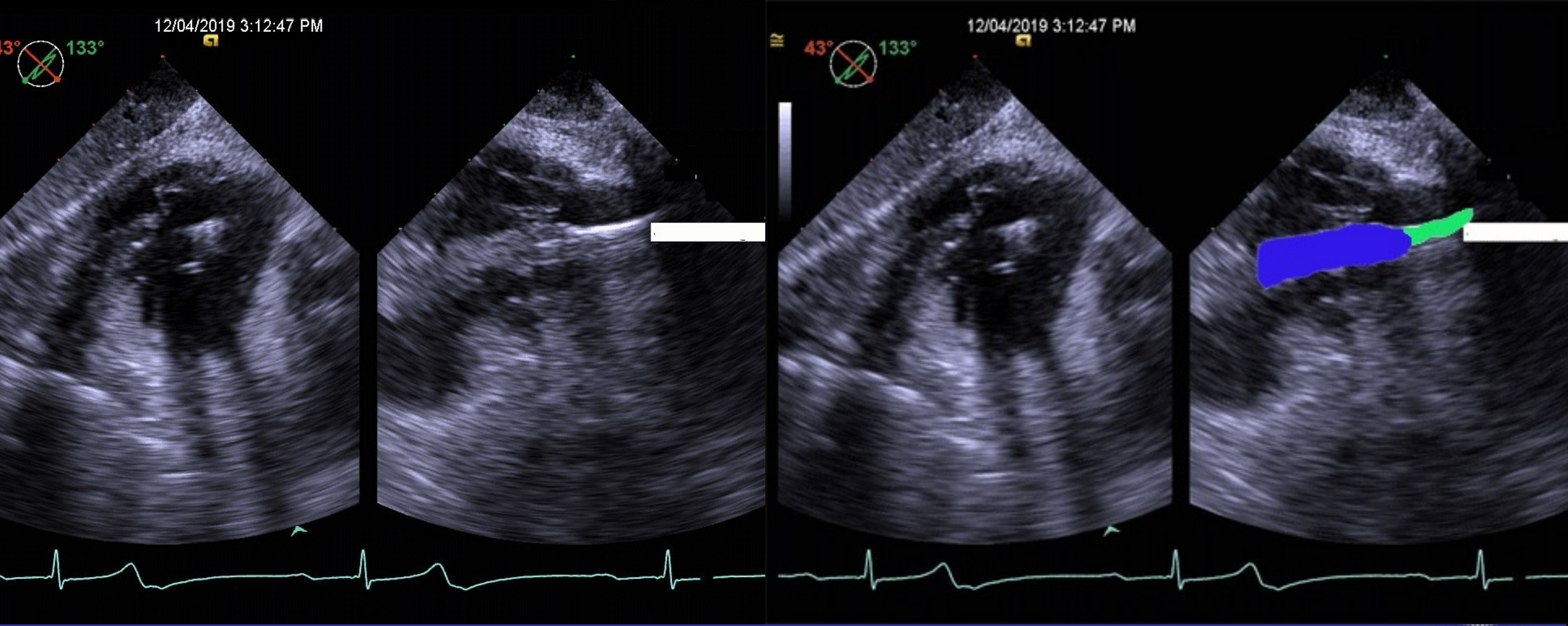

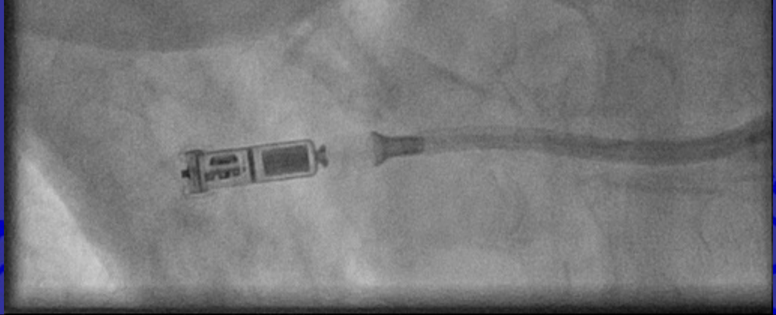

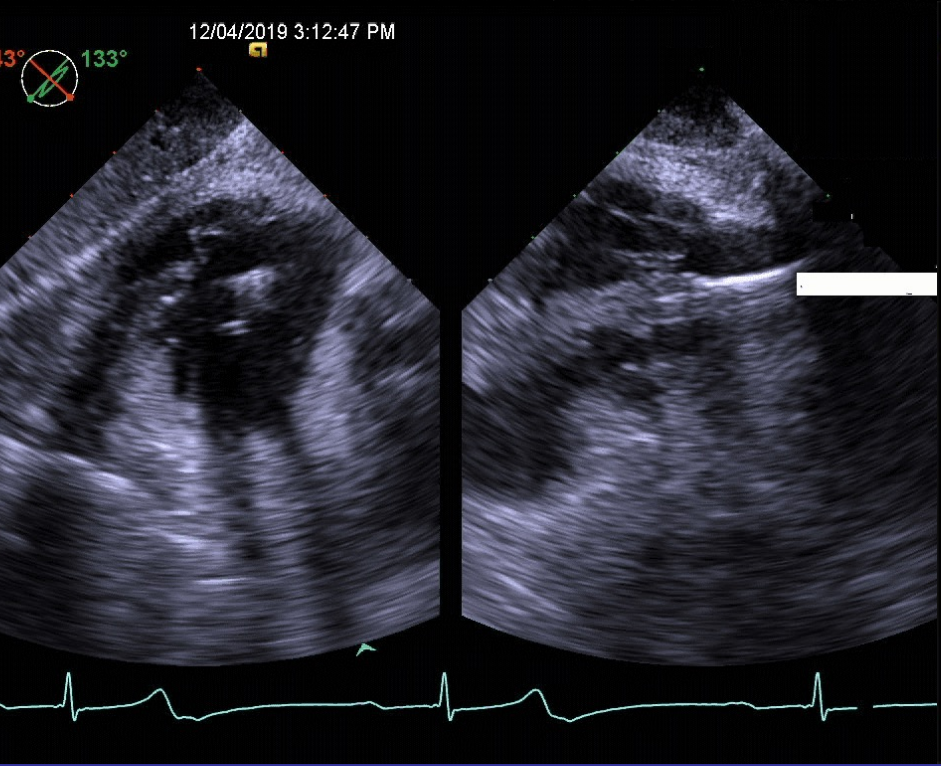
12/04/2019 3:12:47 PM
133°

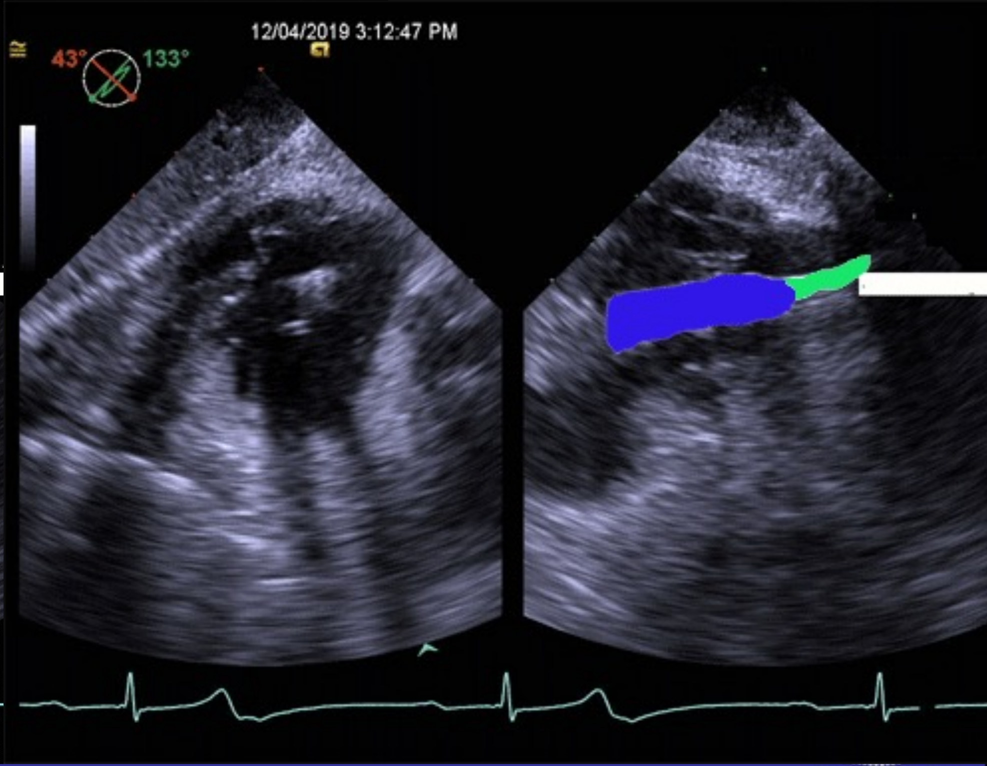
12/04/2019 3:12:47 PM
43°
133°

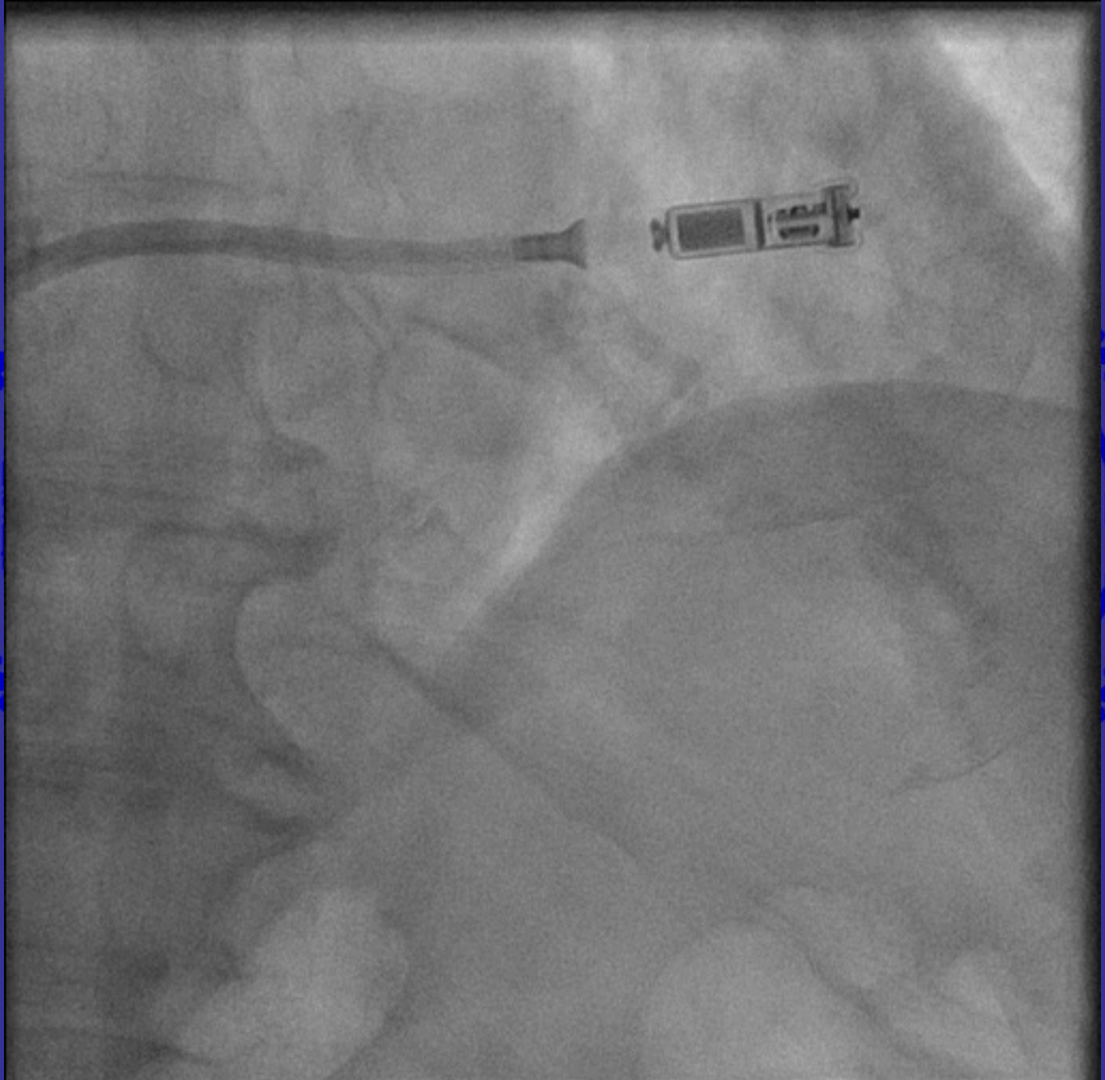

Micra releasing moment: tether still attached

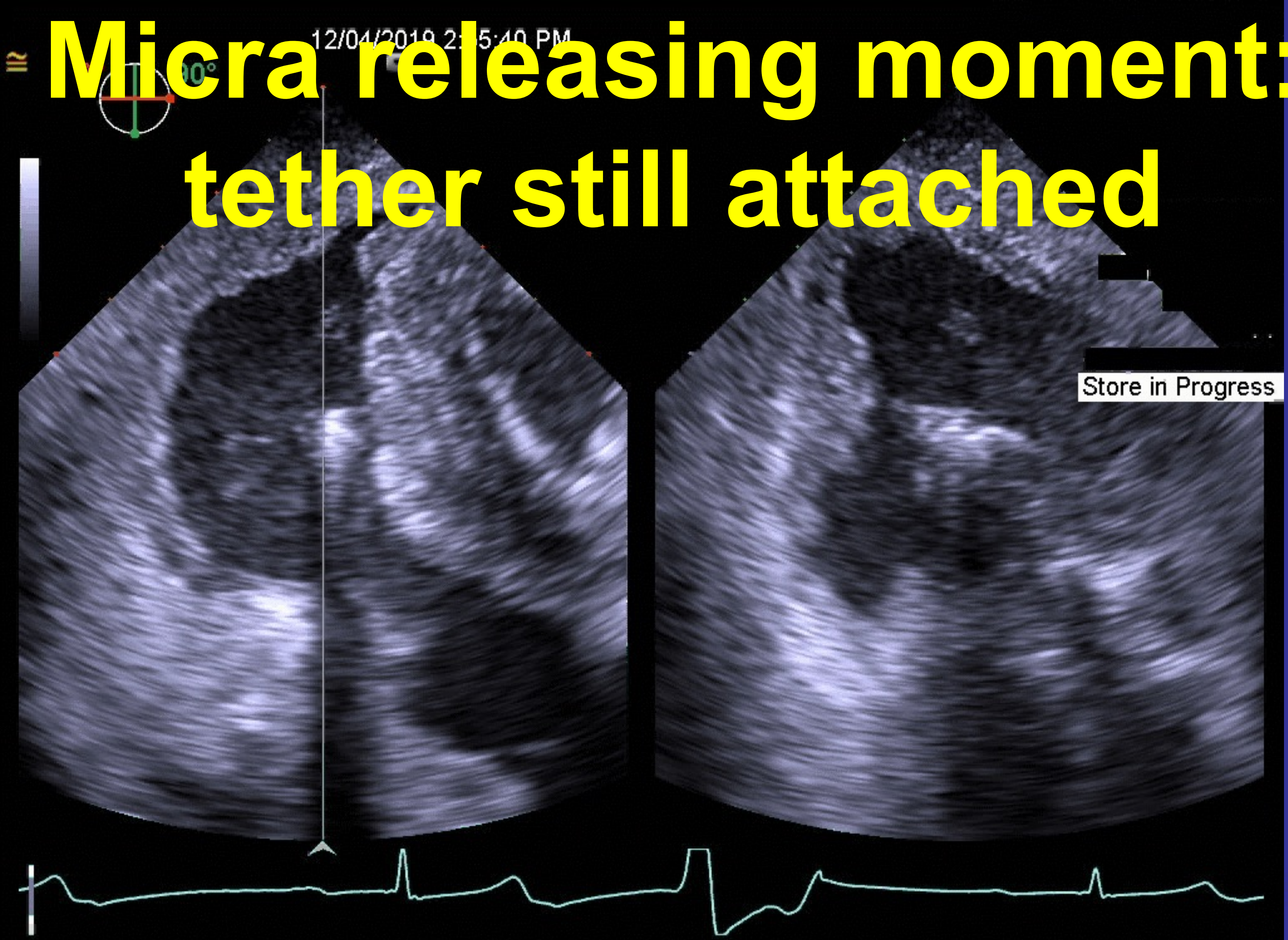

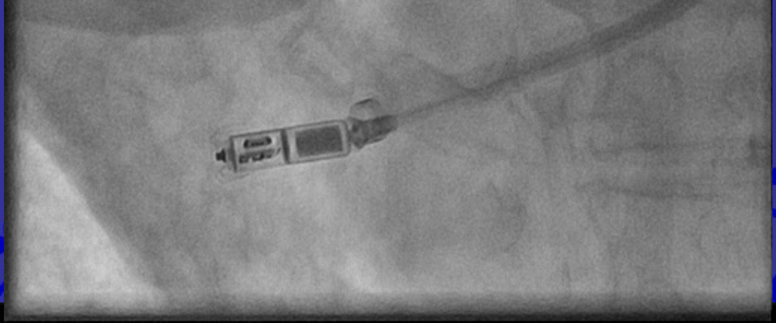

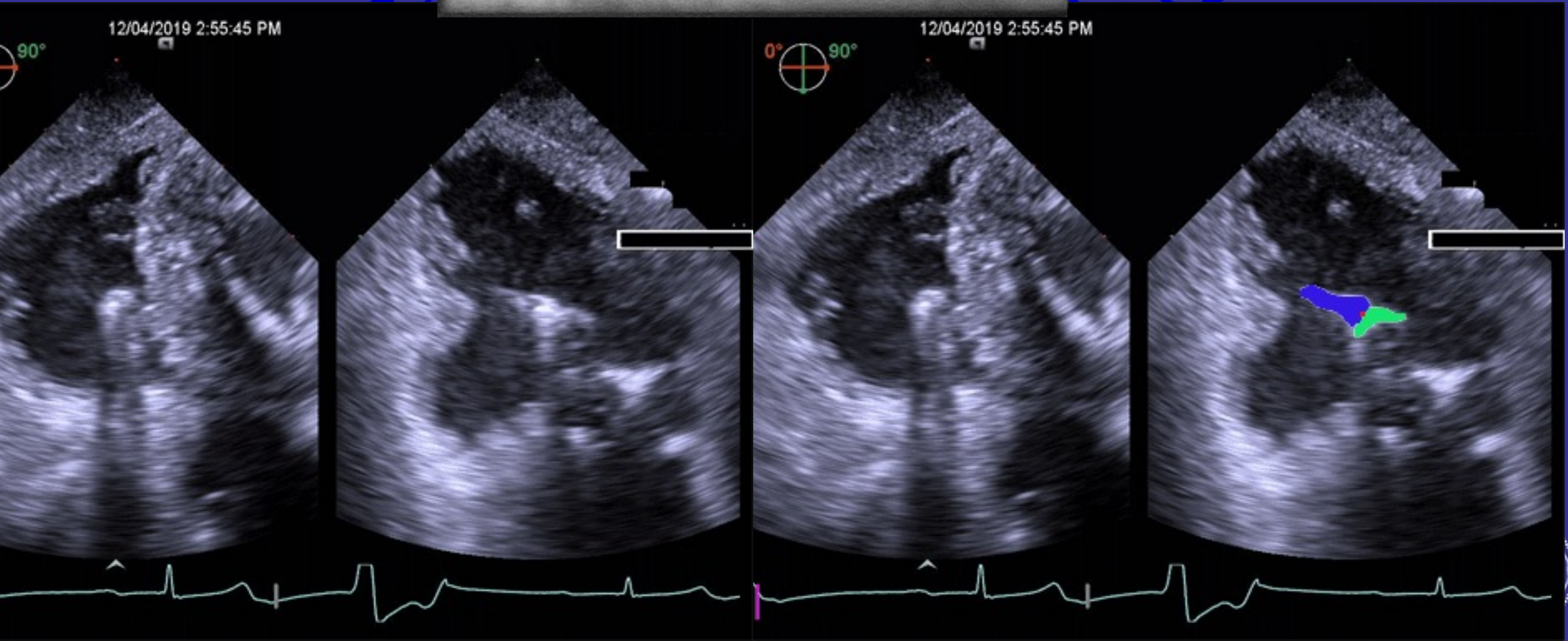
12/04/2019 2:55:45 PM
90°
12/04/2019 2:55:45 PM
0°
90°

Micra releasing moment: tether still attached - Demo

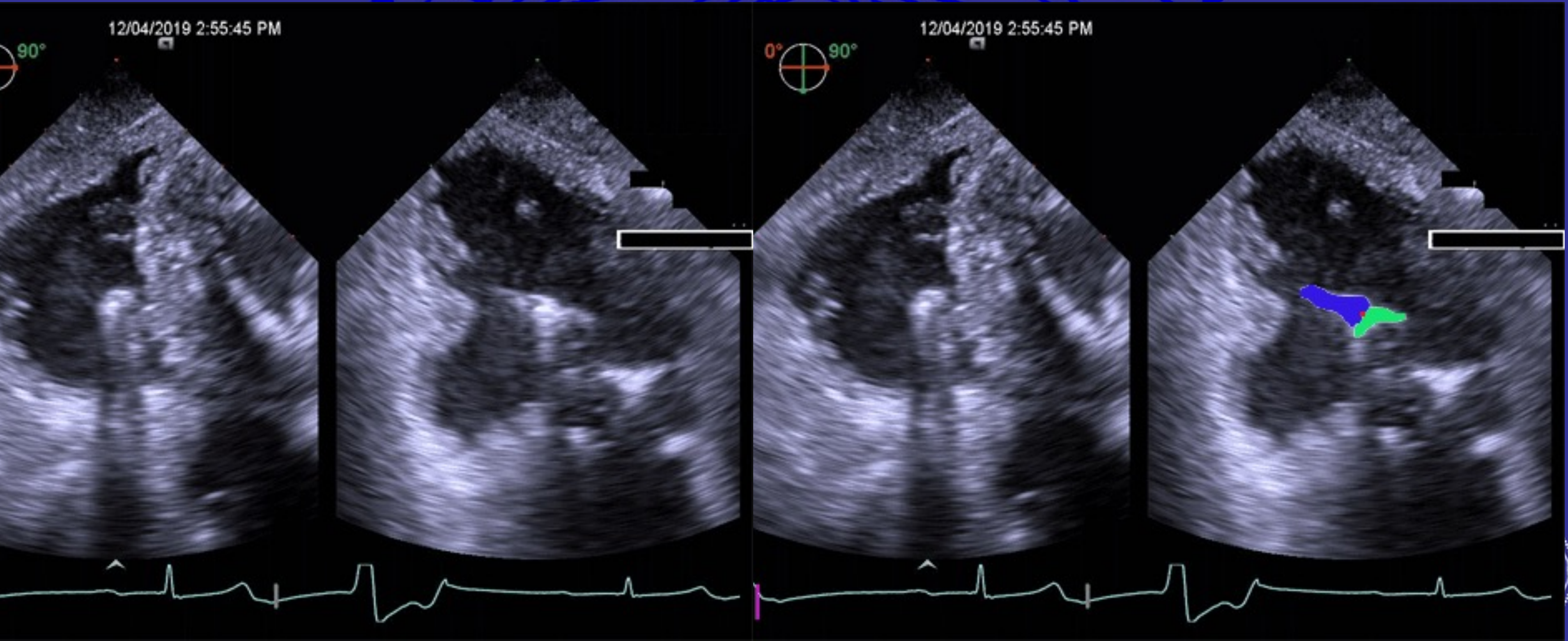

Micra released tether still attached
Septum well seen

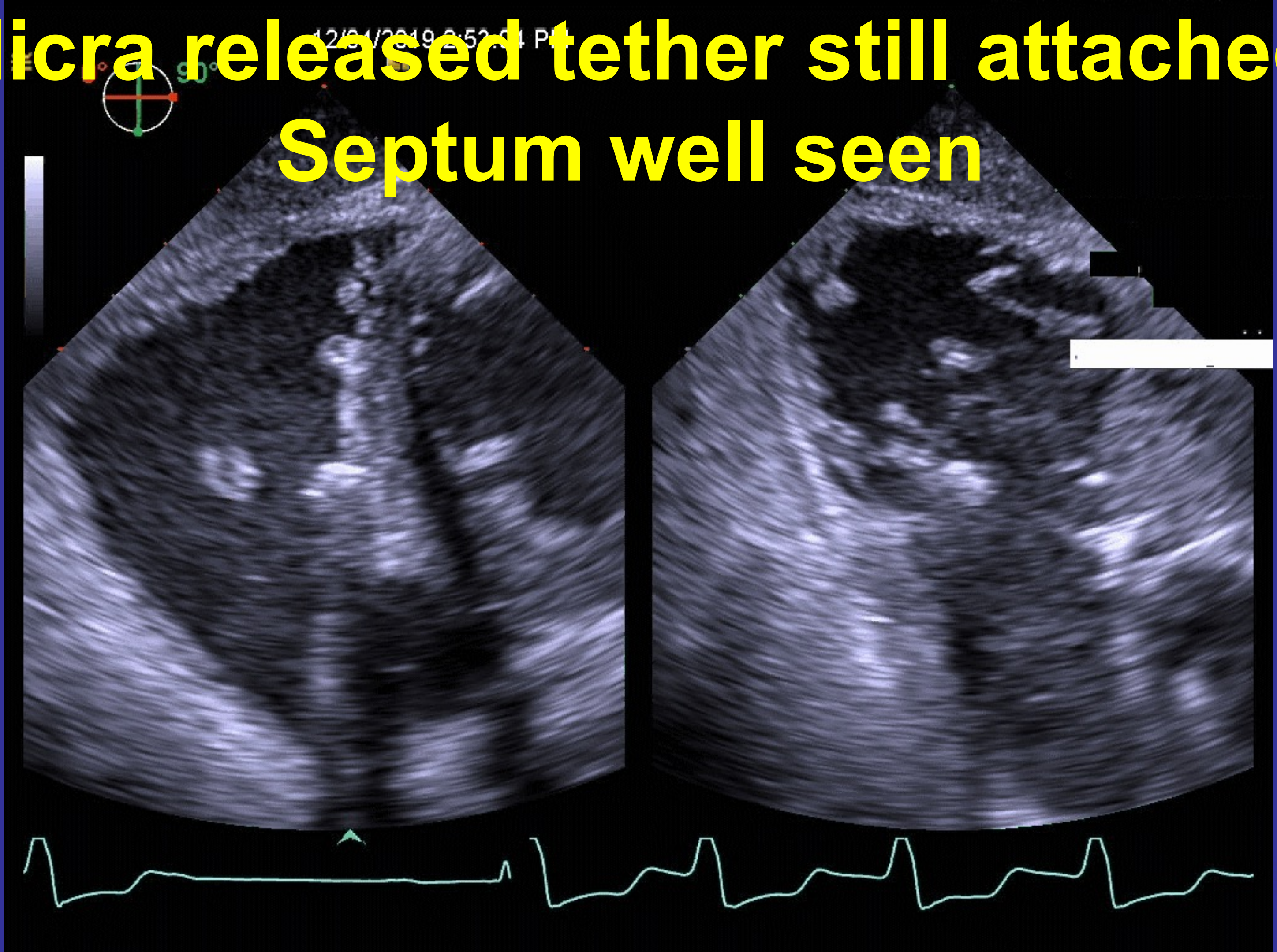

Micra released tether still attached
Septum in and out

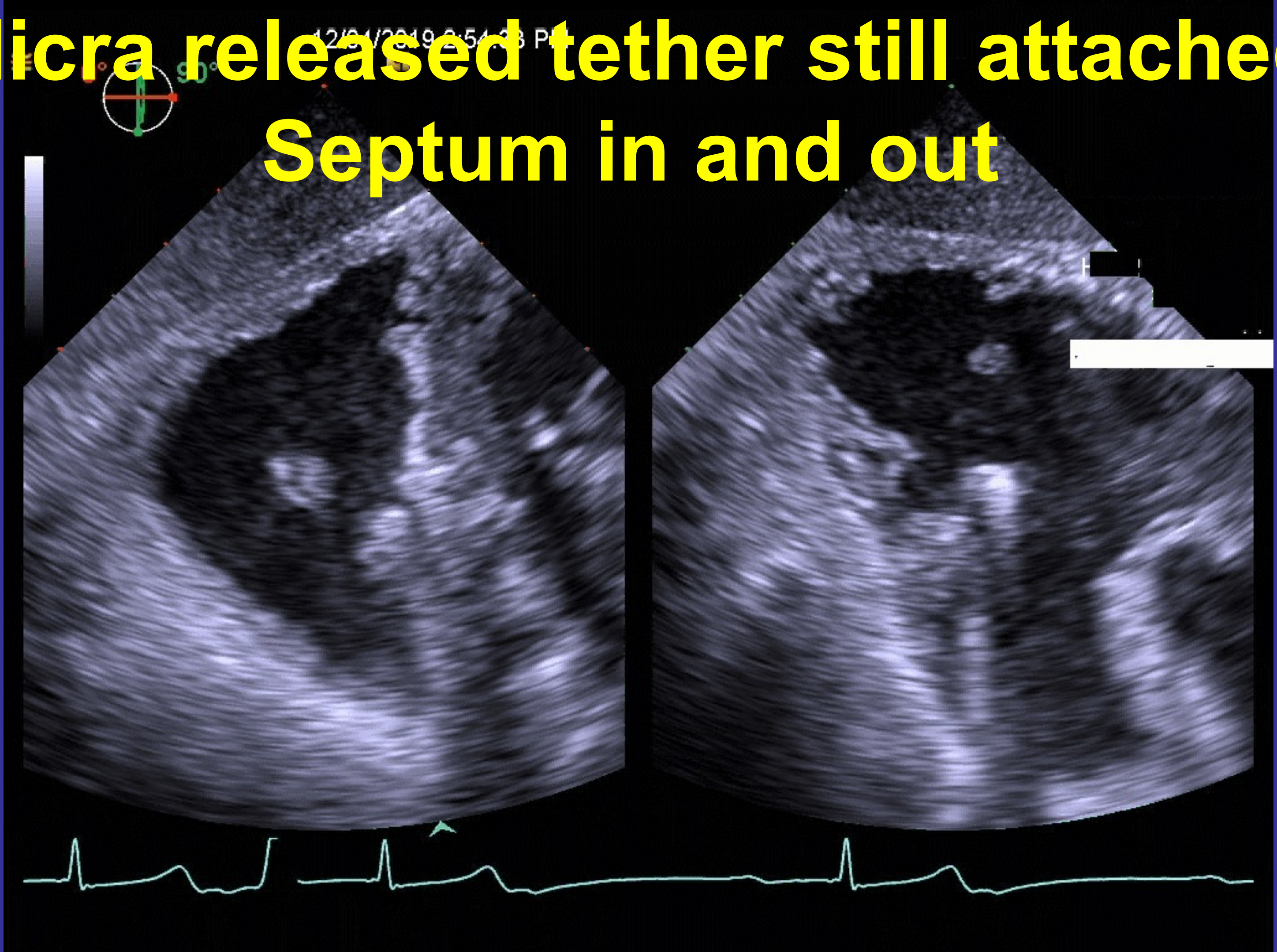

Micra released tether still attached
Tether in and out

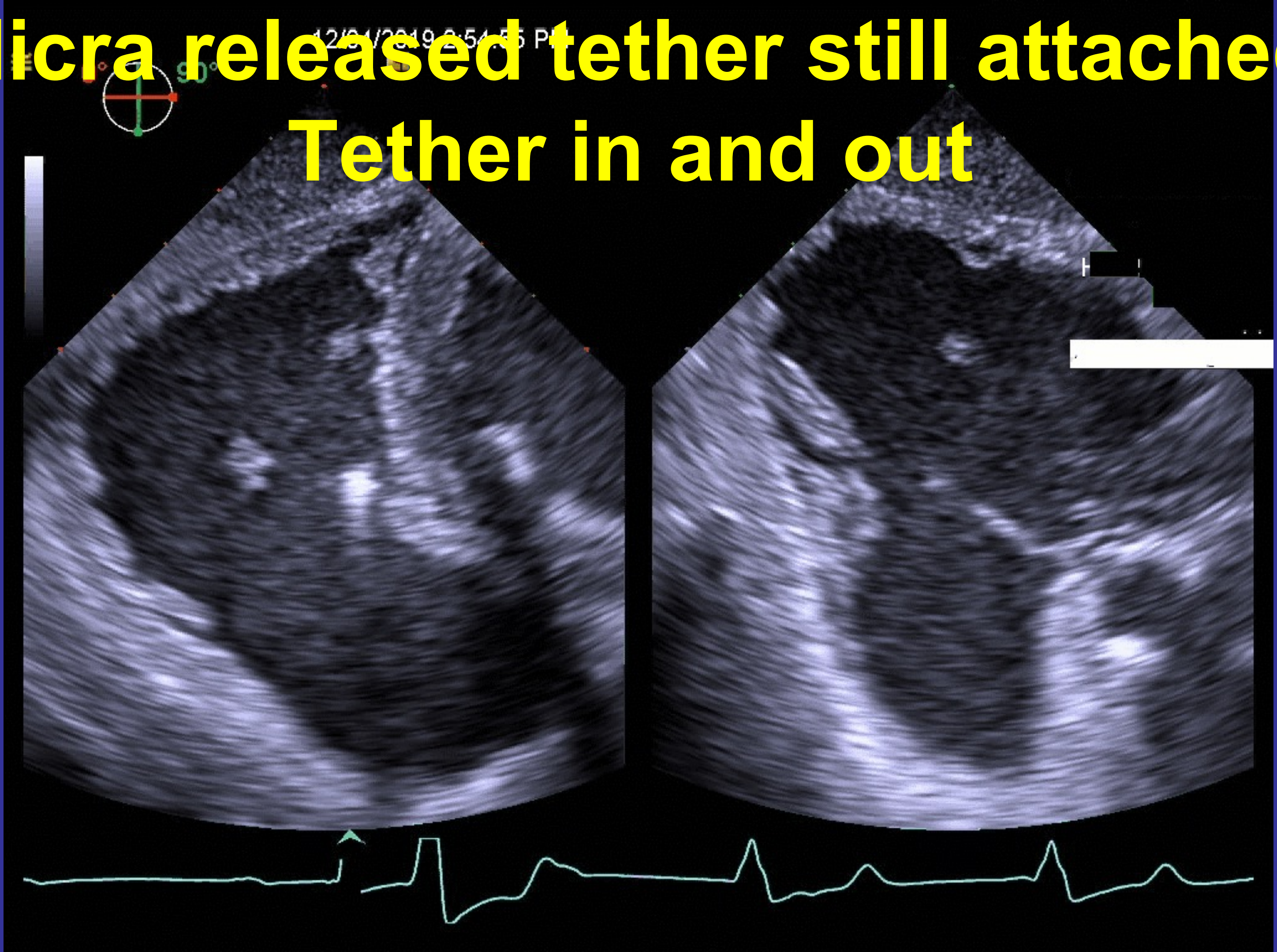

Micra released Sweep: Tether to septum

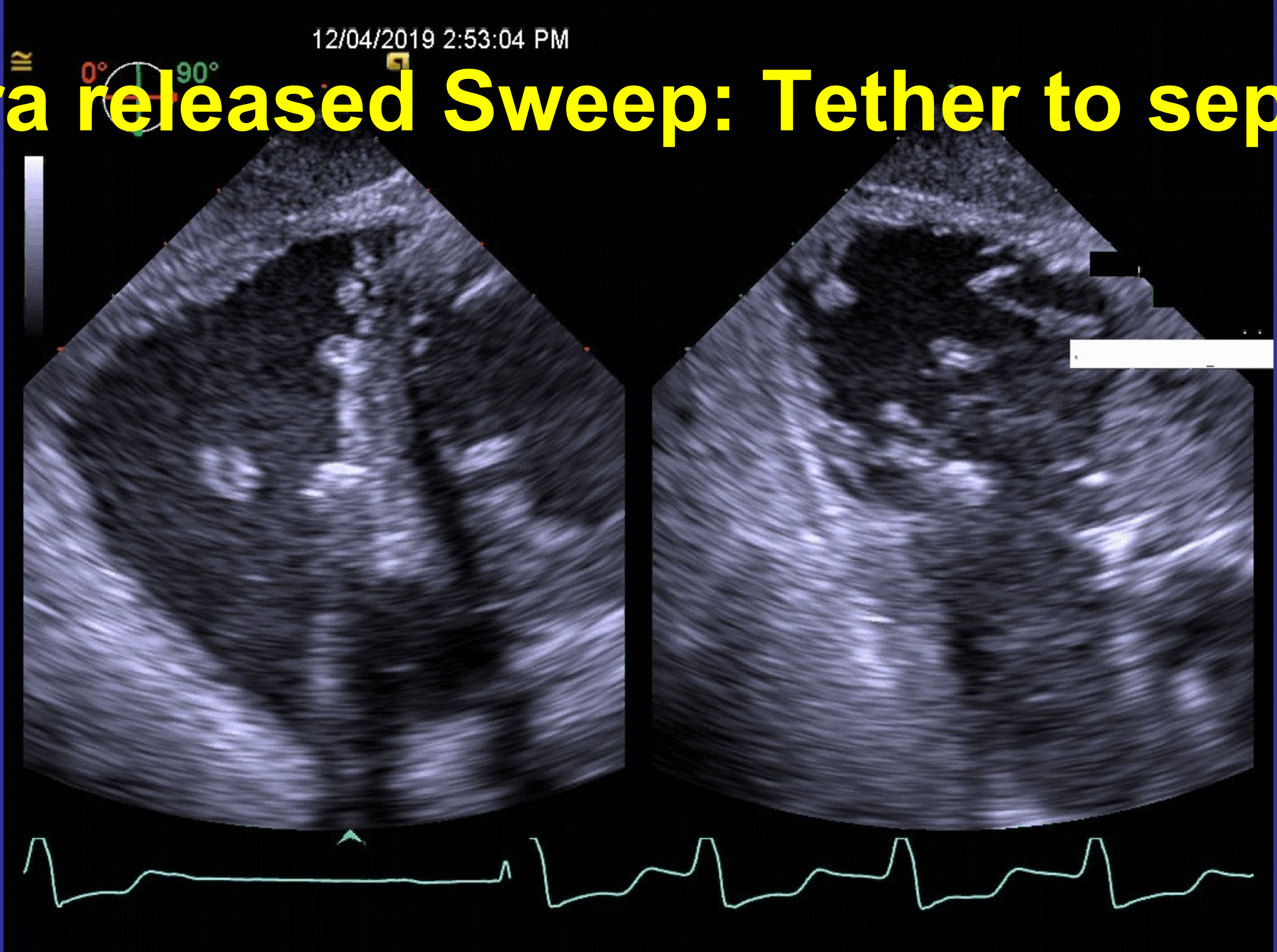

Micra released: Tether to septum, Delivery system still in the RV

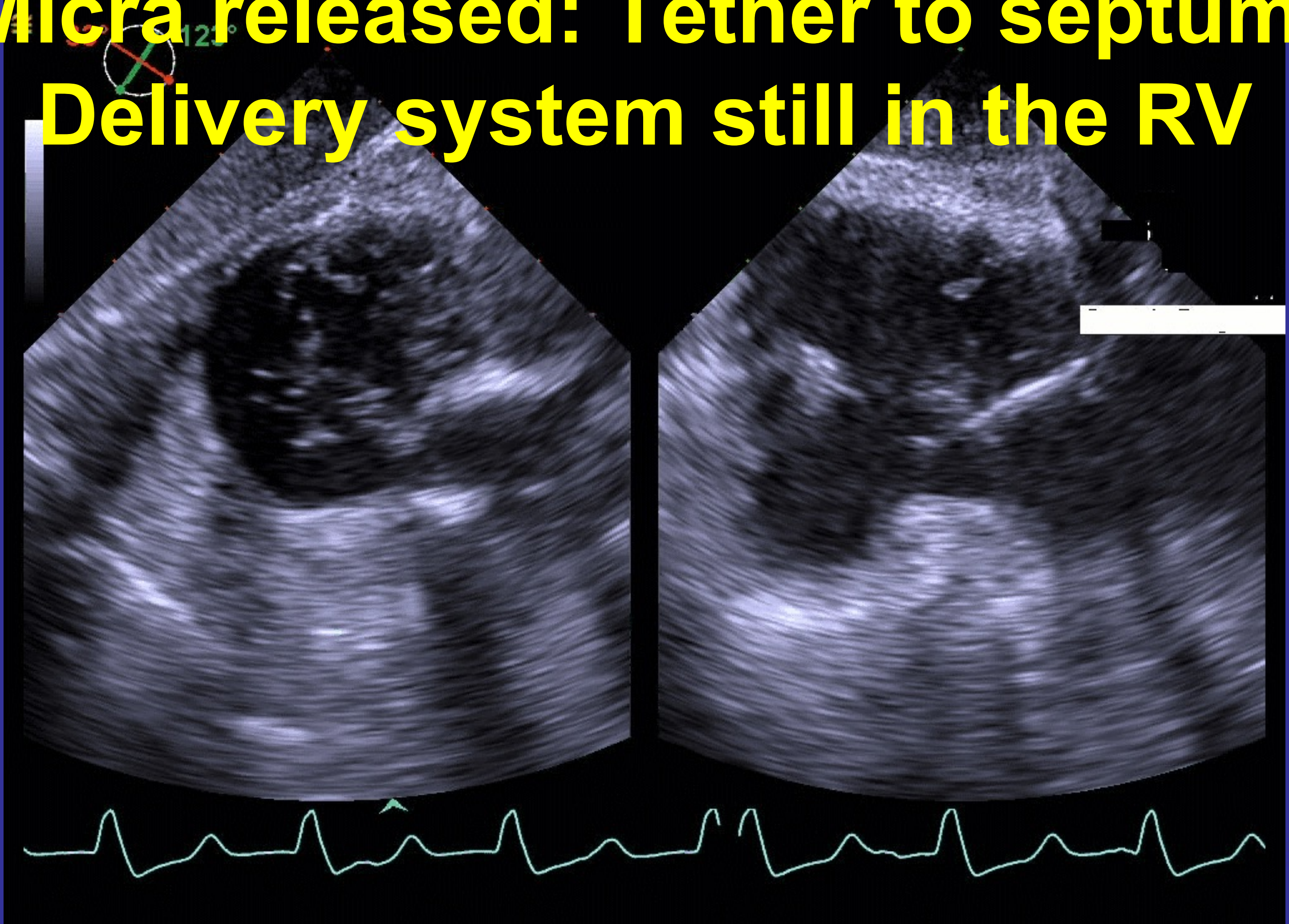

Micra released: Tether to septum, Delivery system out to the RA

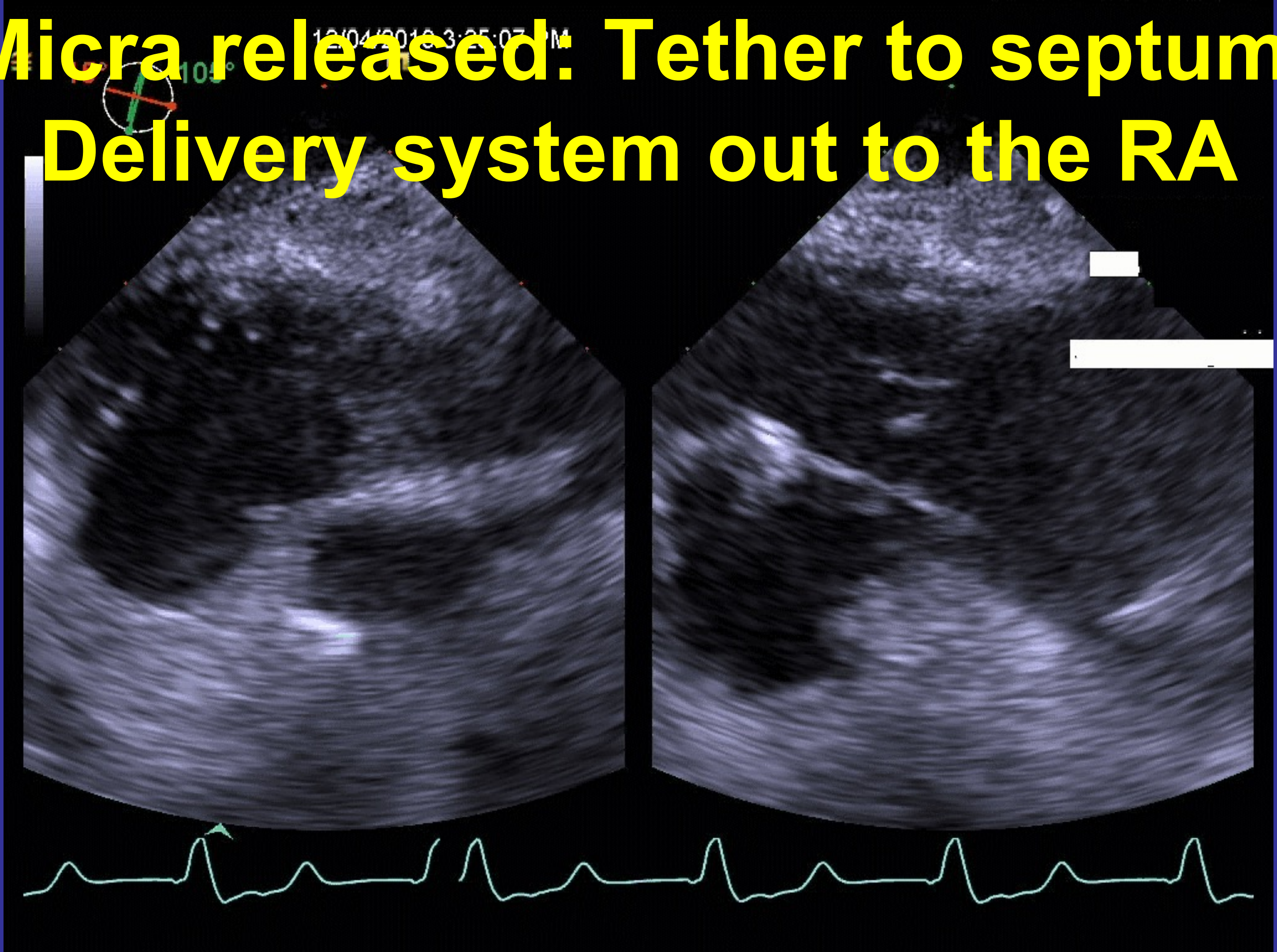

Micra released:

Delivery System – RV Delivery system - RA

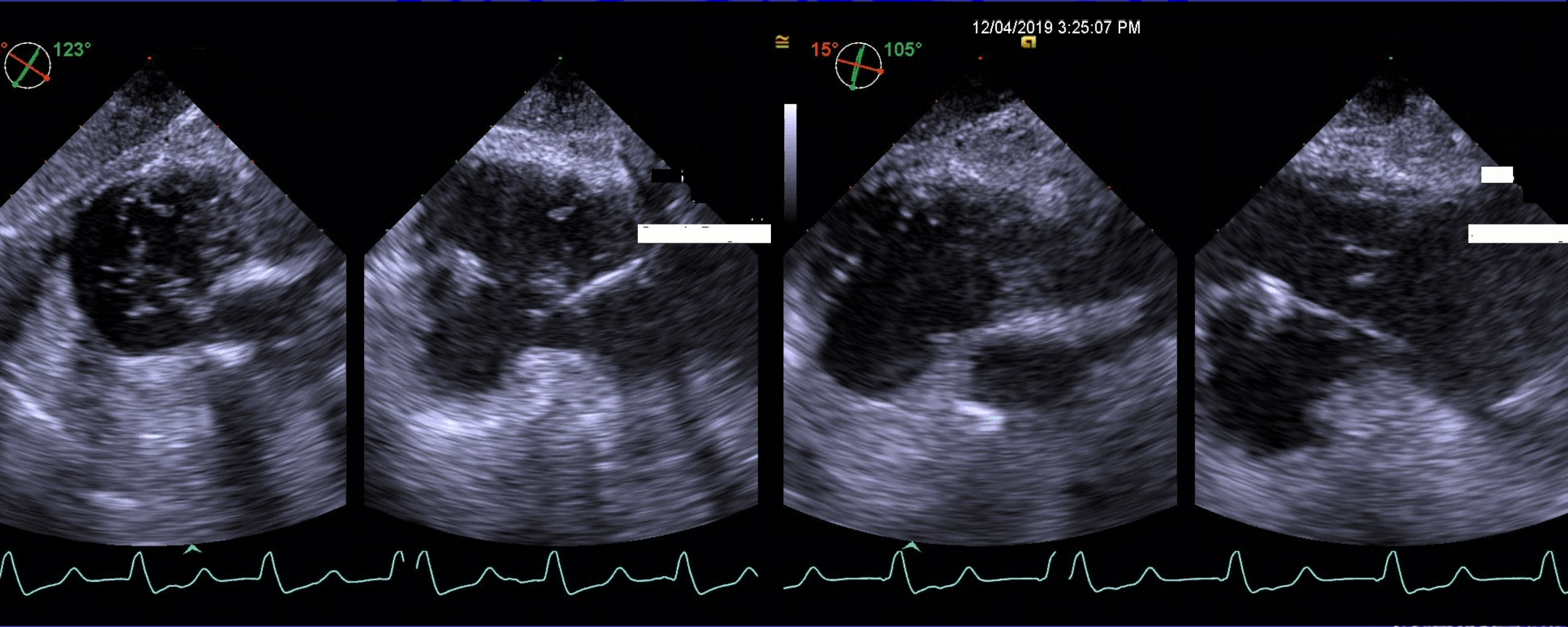

Micra released:

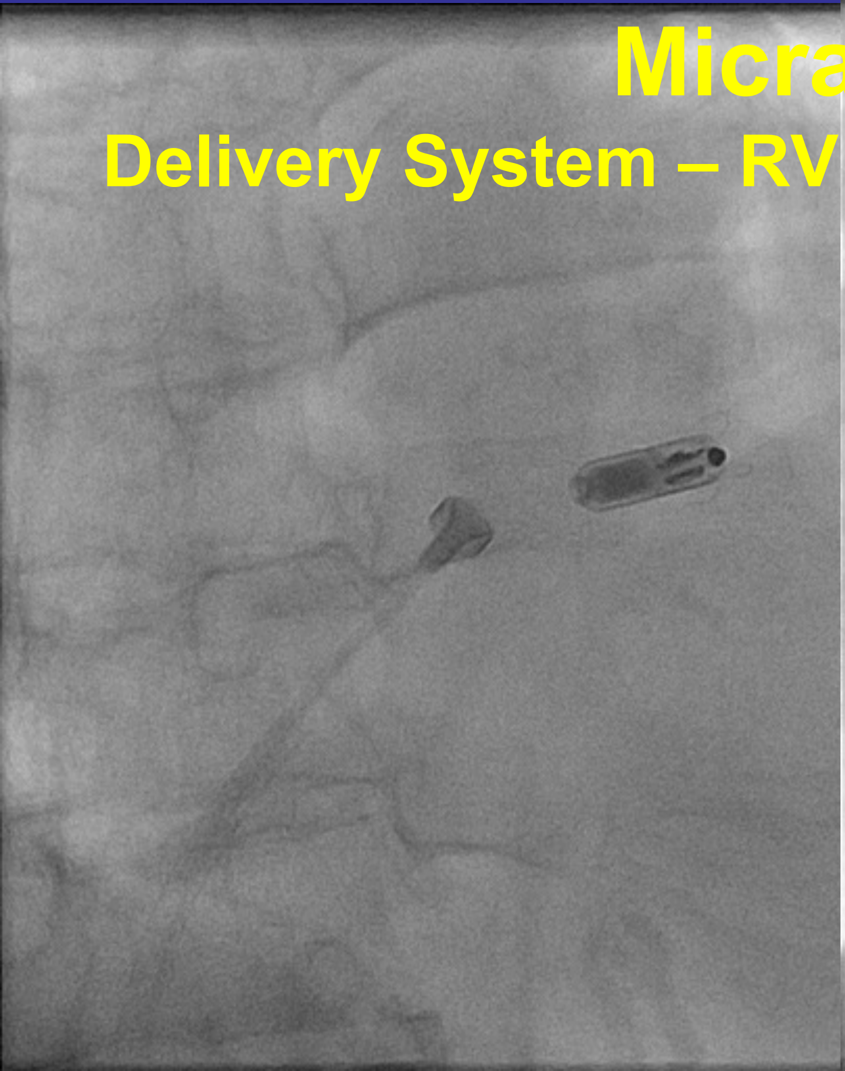

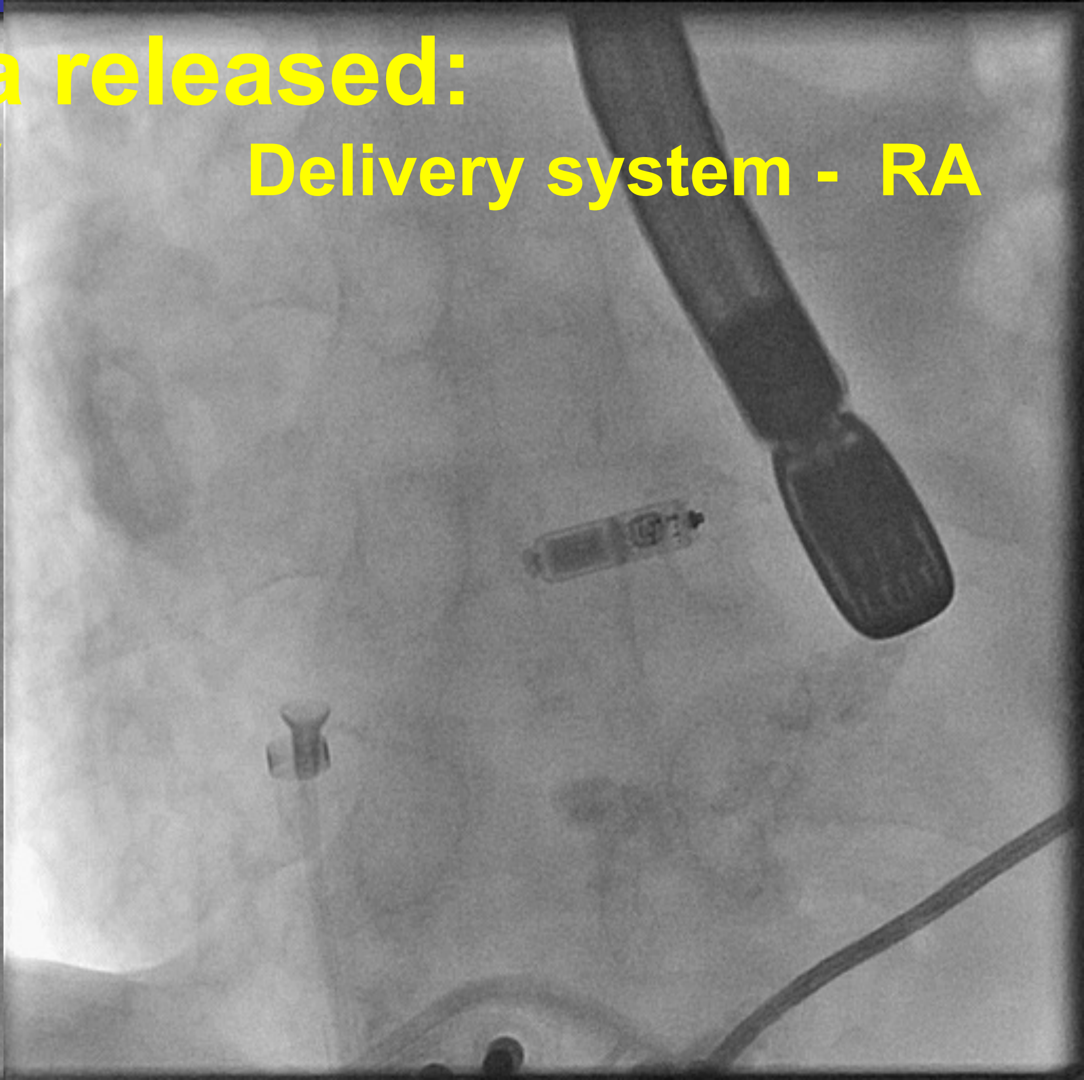

Micra released: Delivery System – RV

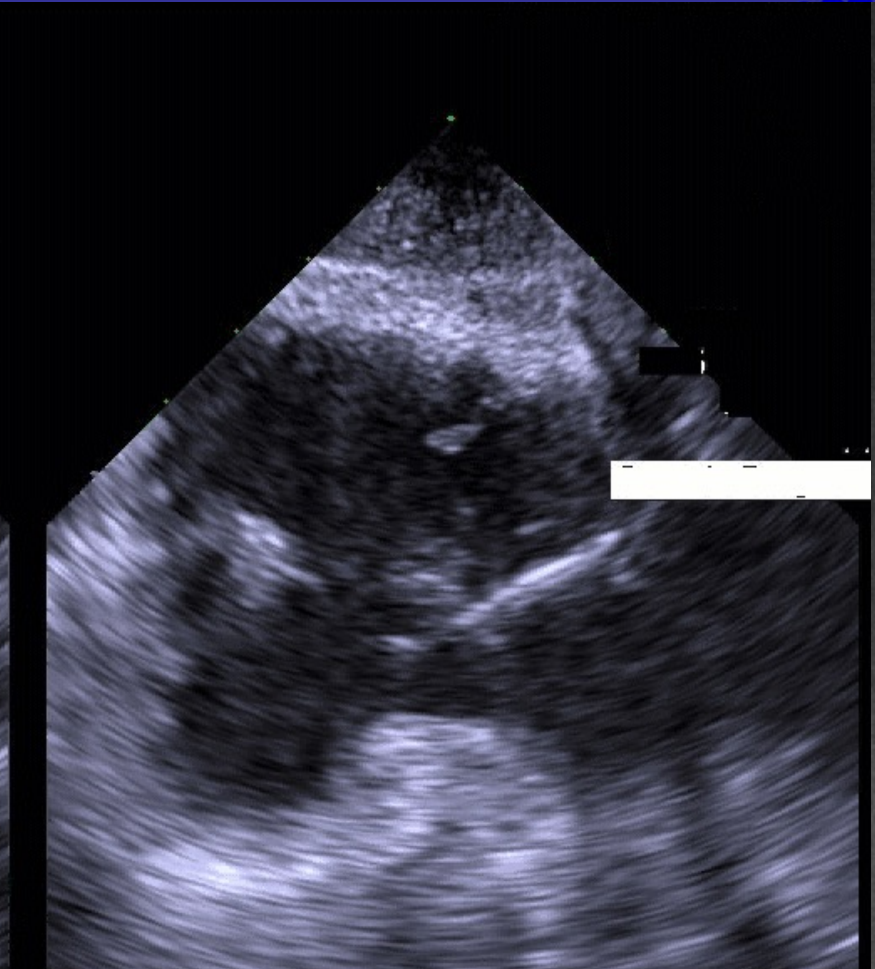

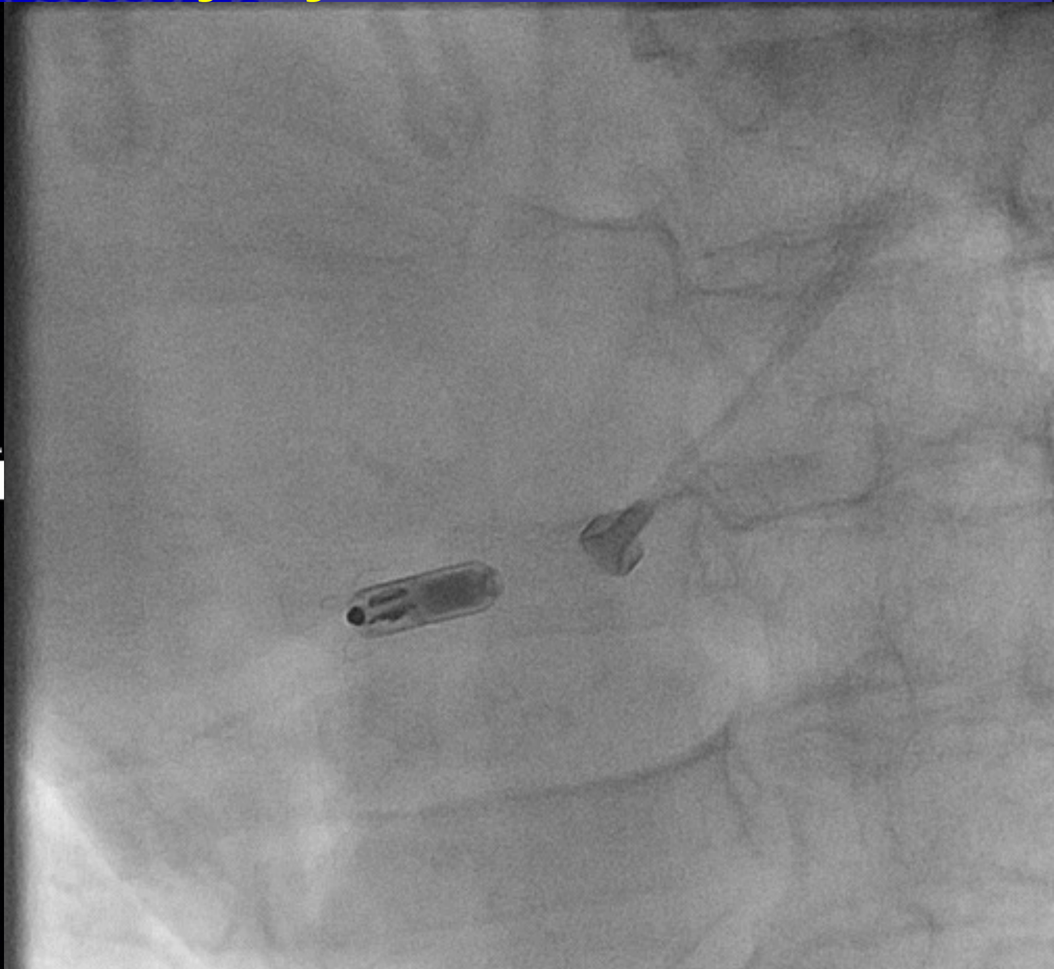

Micra released: Delivery system - RA

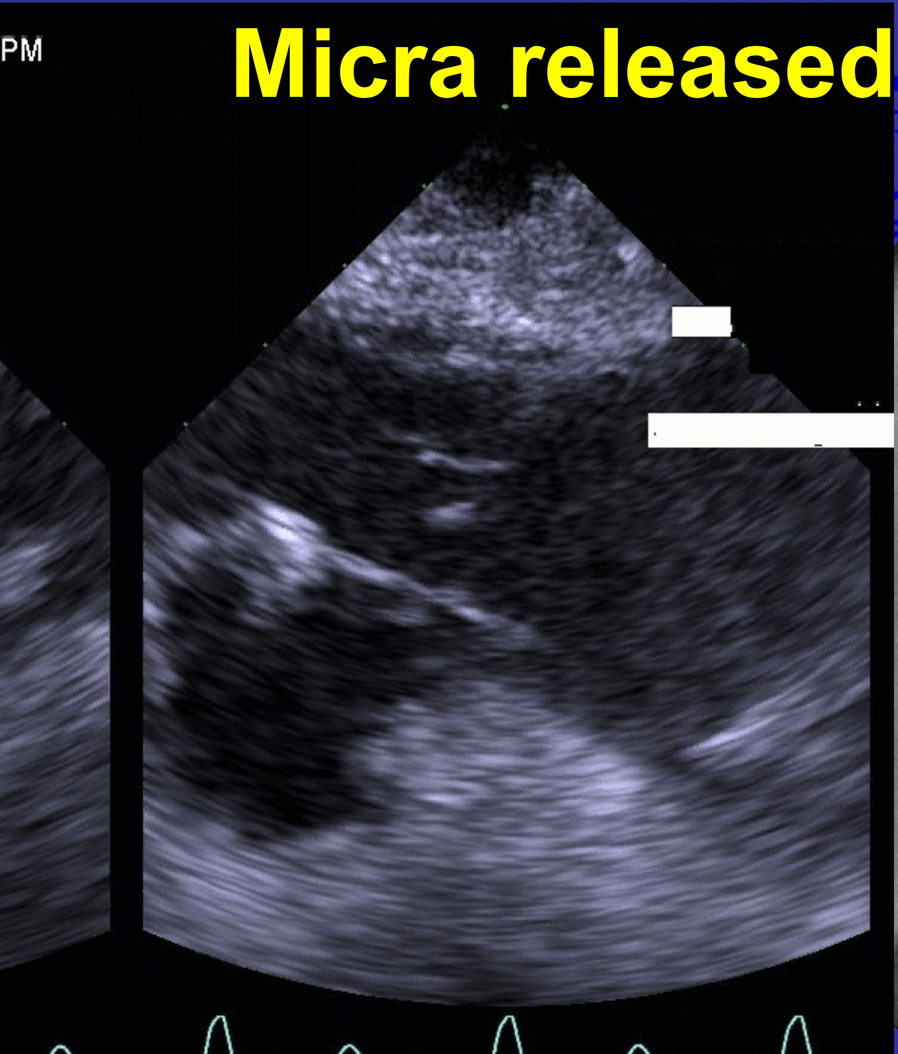

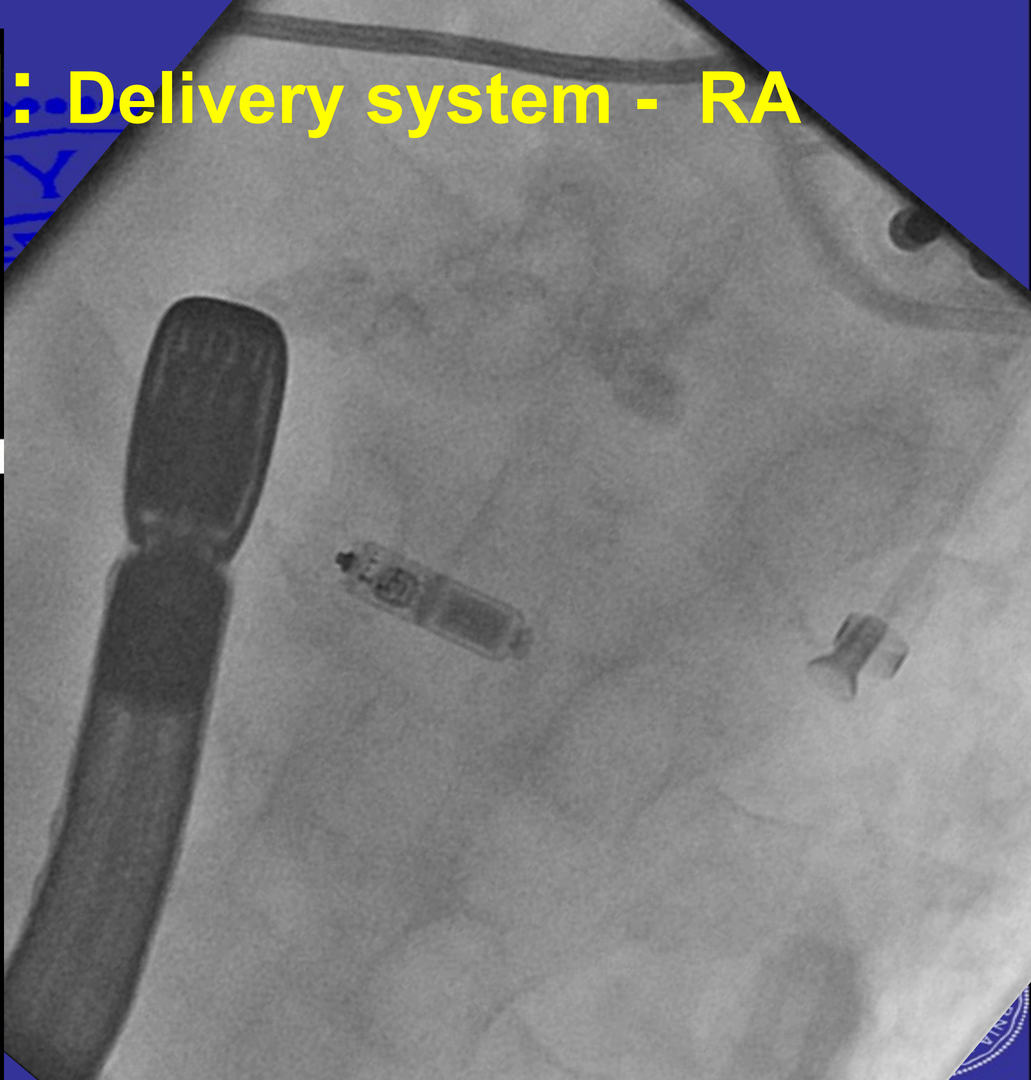

If Unable to Attain TG-View: Mid Esophageal 2D, Biplan, 3D

Micra Delivery Sheath Mid Esophageal 2D vs 3D

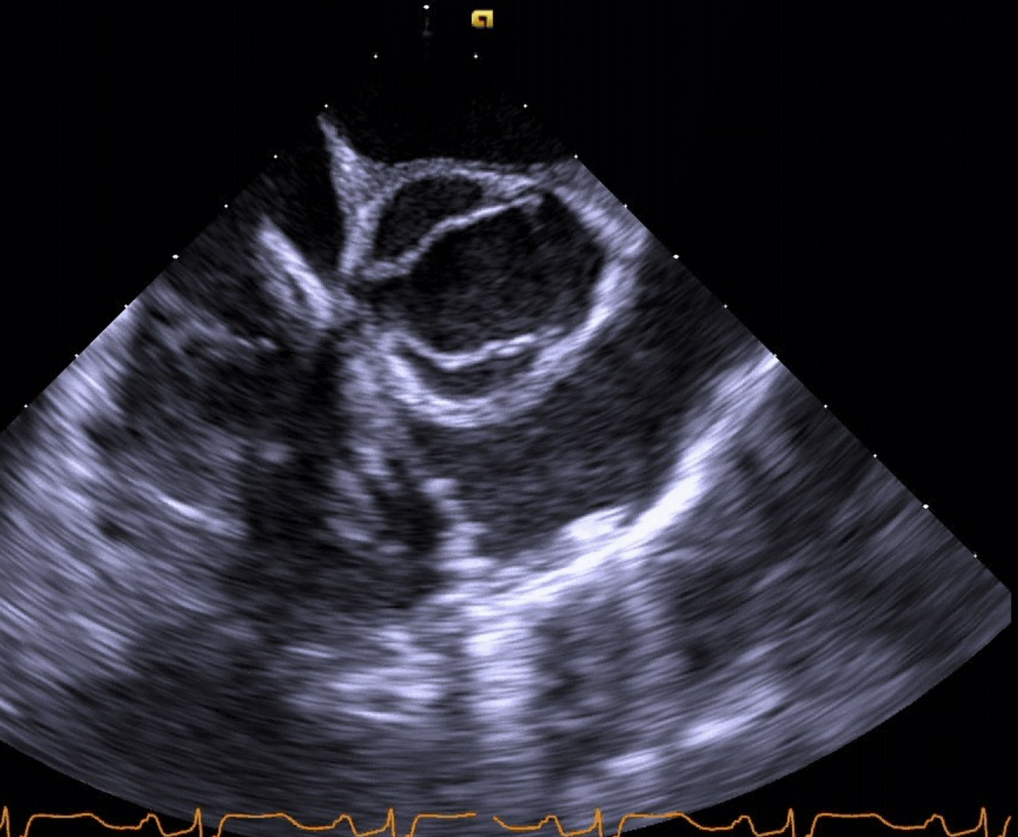

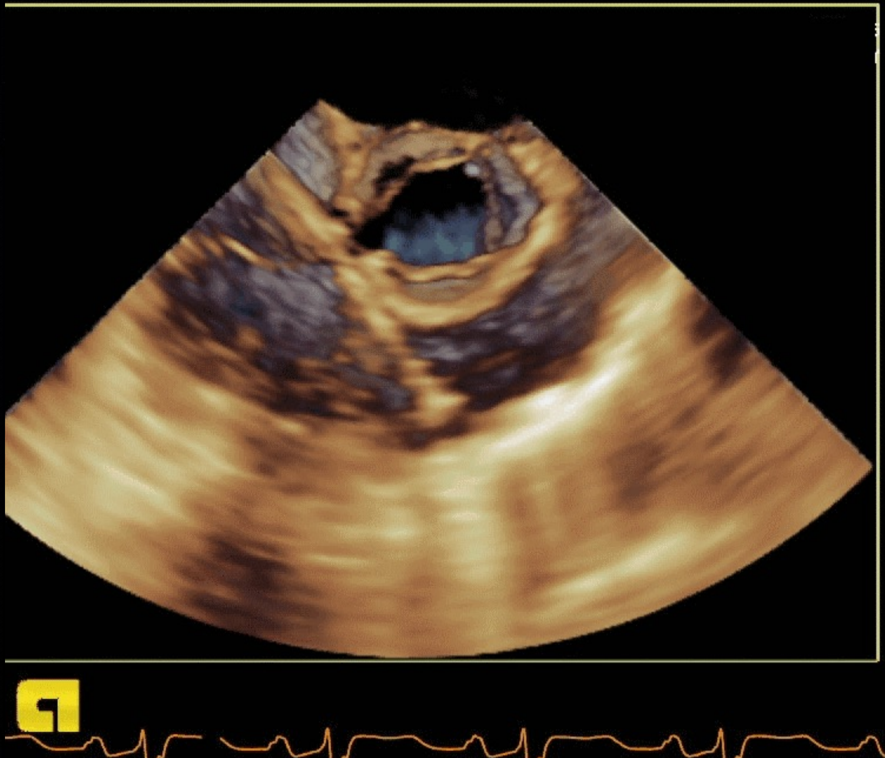

Micra Deployment
Mid Esophageal 2D vs 3D

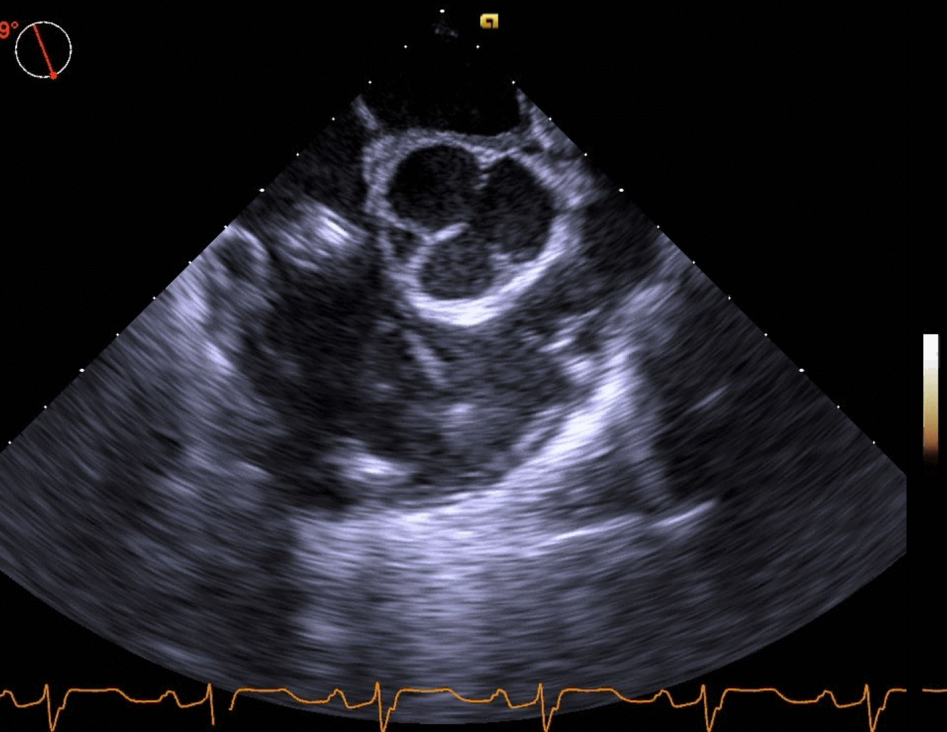

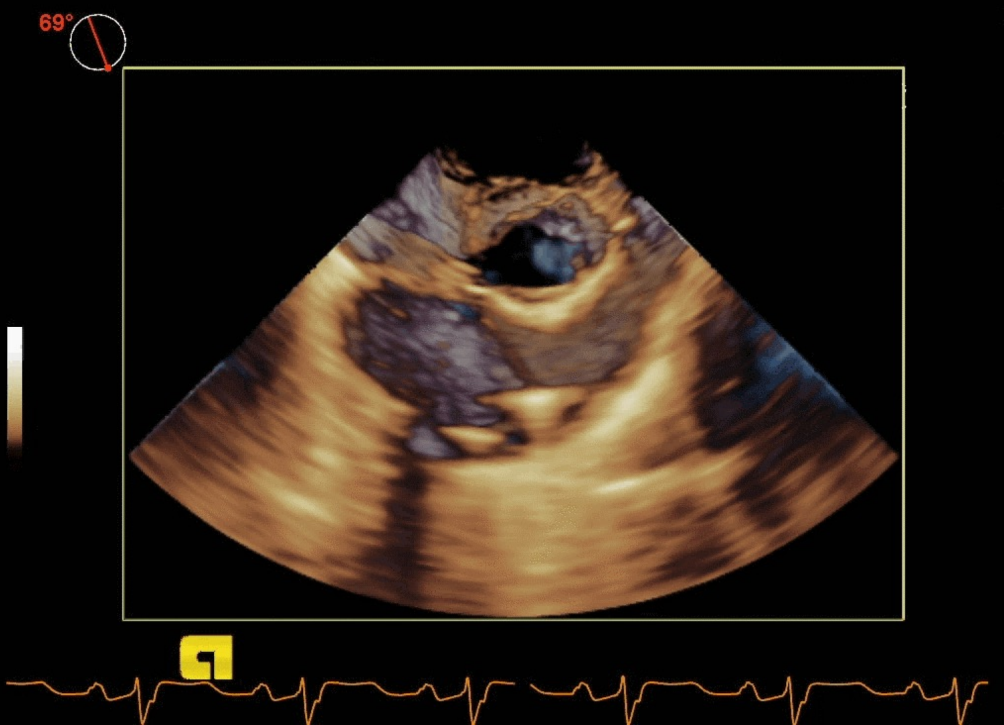

Tether Test & Removal

- Tether (“Tug) Test/ Tether Removal
  - Observe in same trans-gastric biplane view, tether and device at deployed location.
  - If appropriate tether test, monitor device and tether as tether cut and removed
  - If not appropriate tether test, delivery sheath is re-aligned with micra device for re-capture. TEE images monitoring sheath, device, and tether until capture is completed. Then reattempt pre-delivery and deployment views.

Tether testing

Micra released: Delivery System re-enter RV

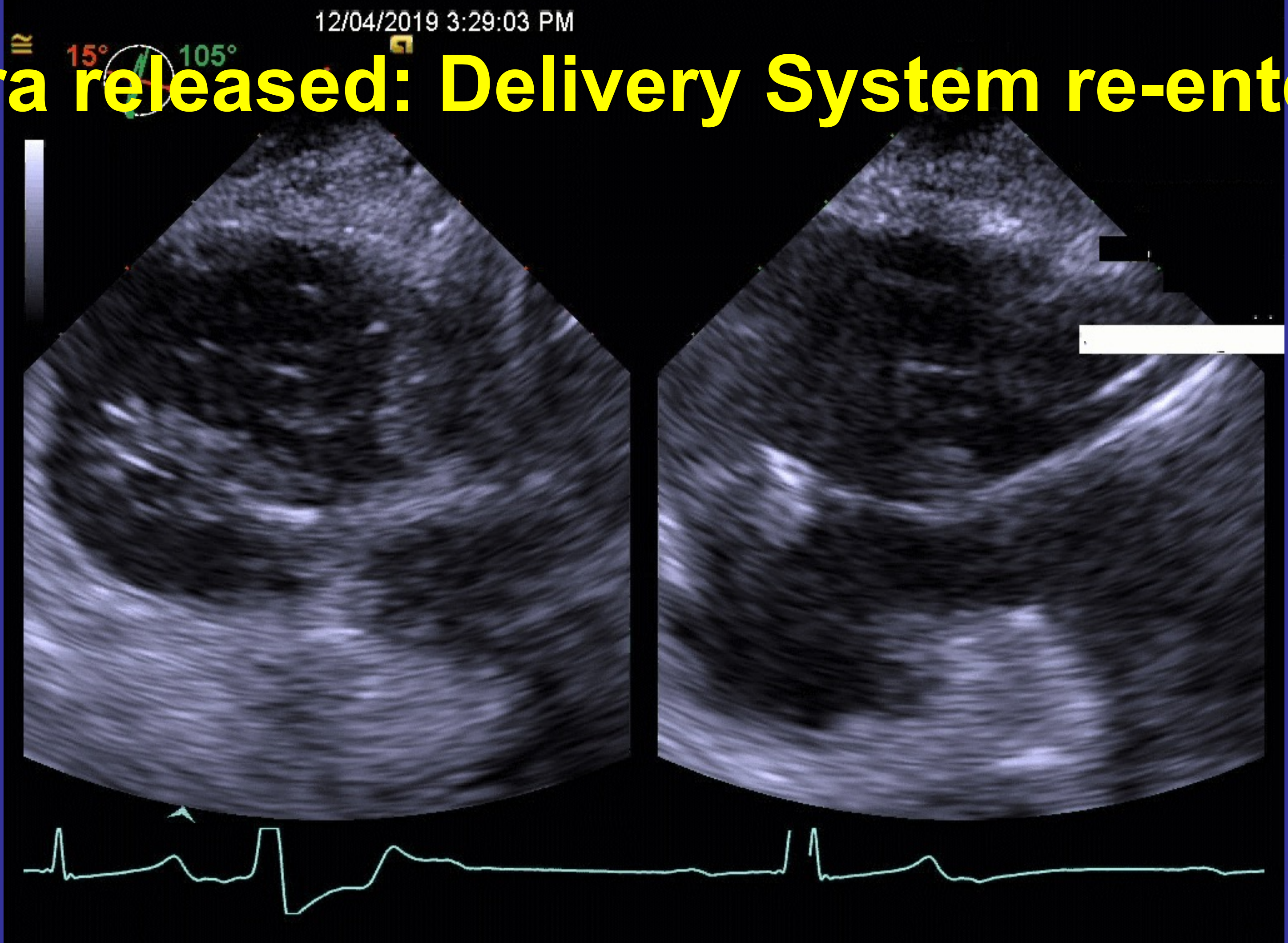

Micra Tether Tug Test

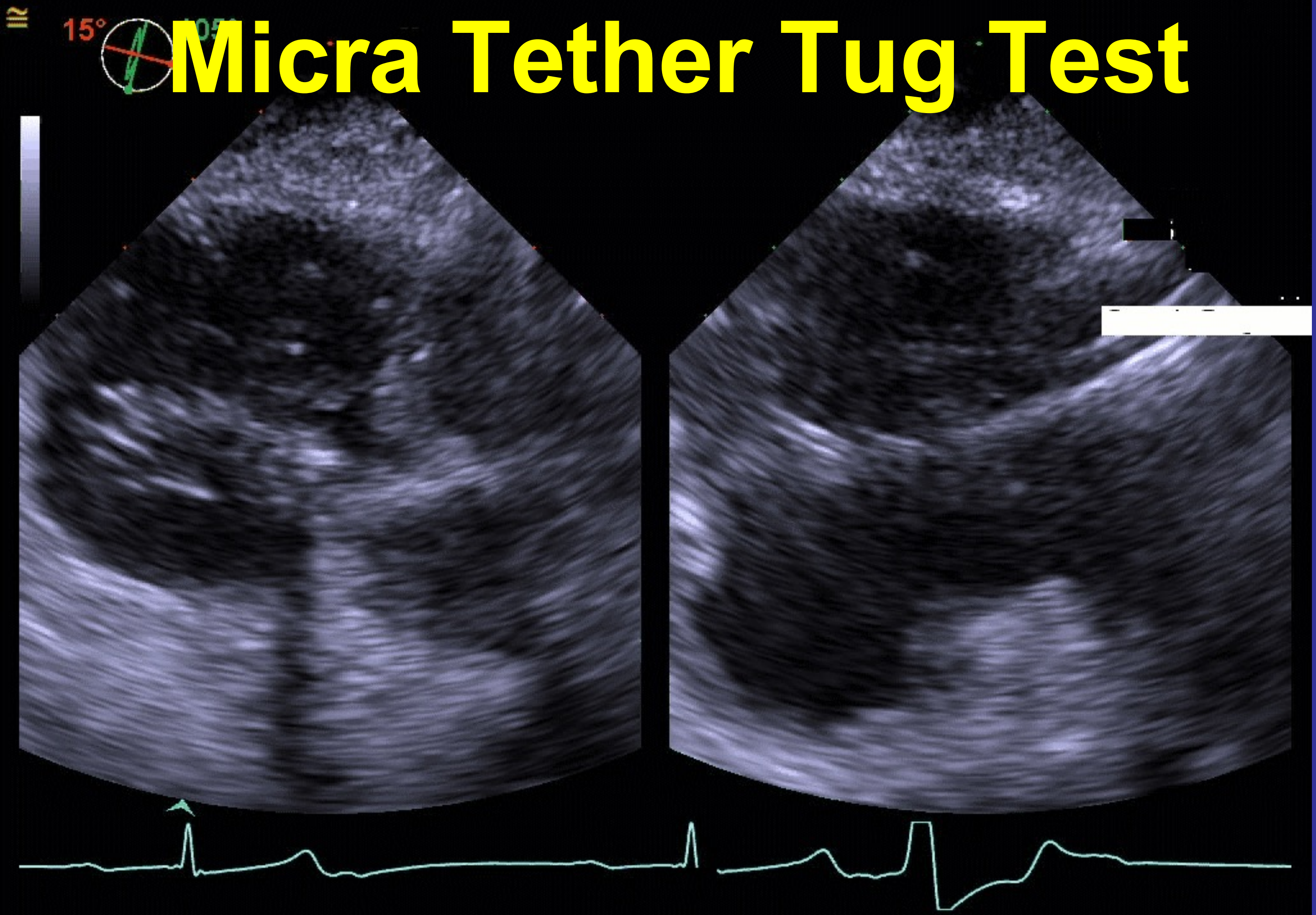

Micra Tether Test Continued

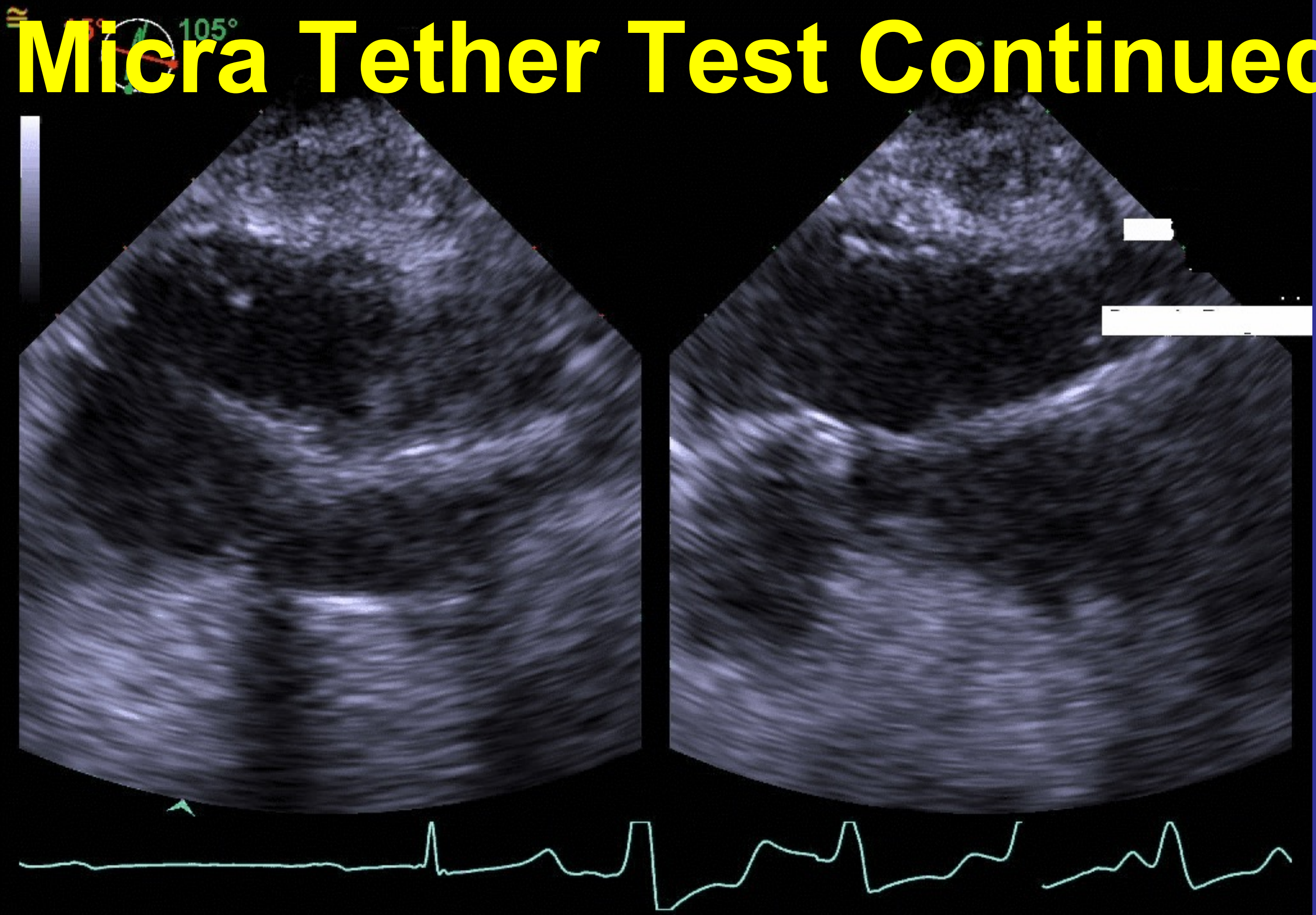

Micra Tether testing

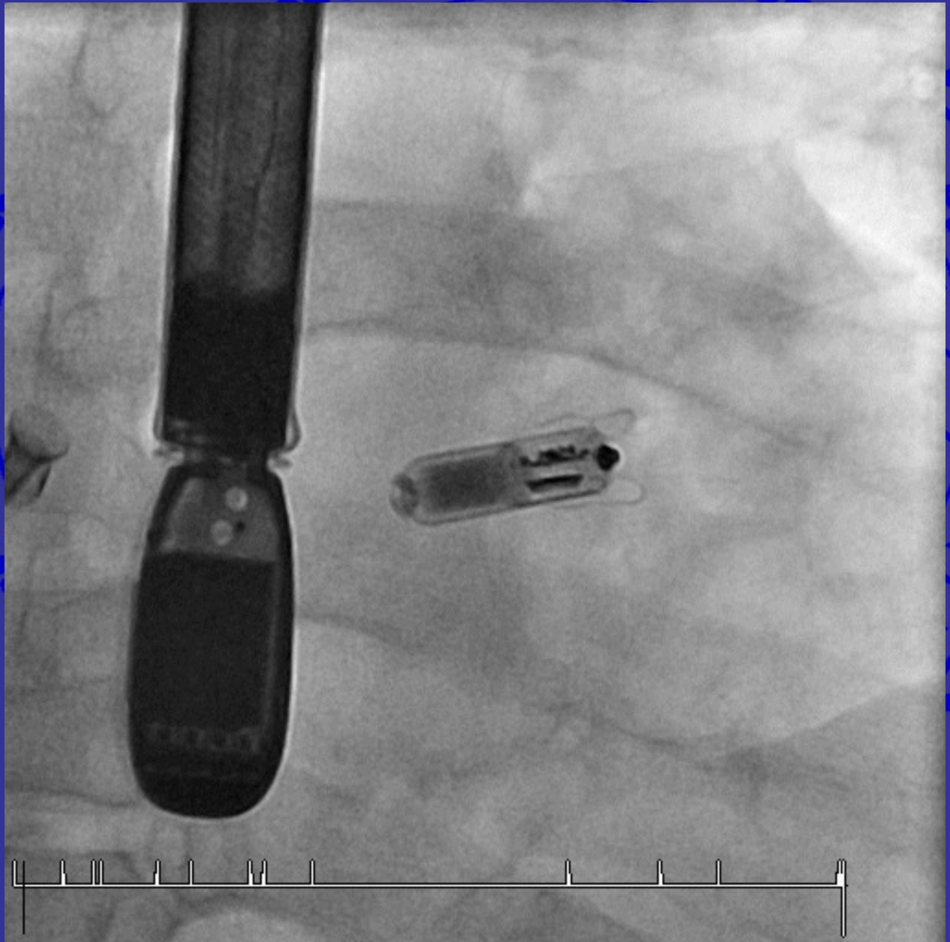

Micra Tether testing failure: too hard, pulled off

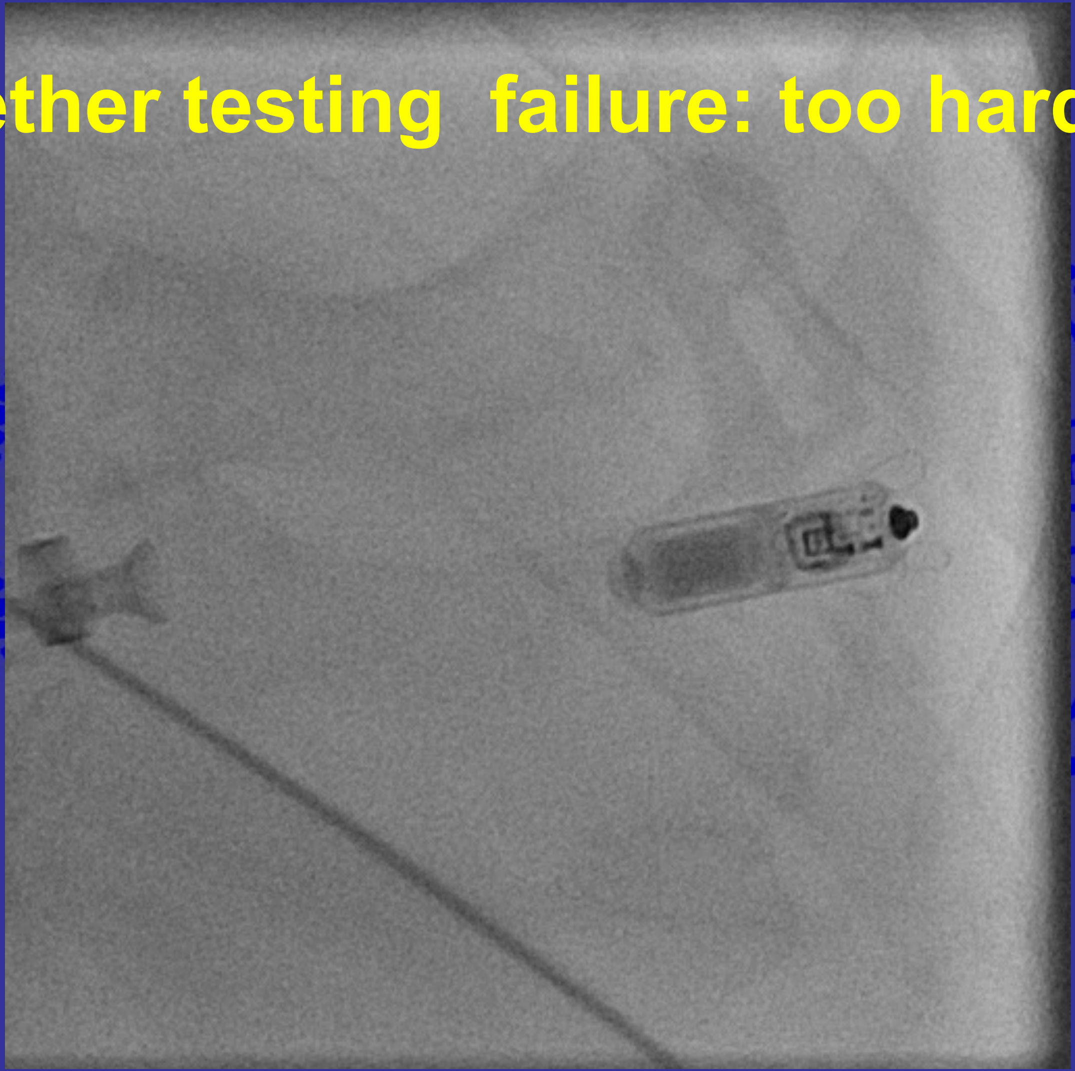

Recapture: Try to re-depoly

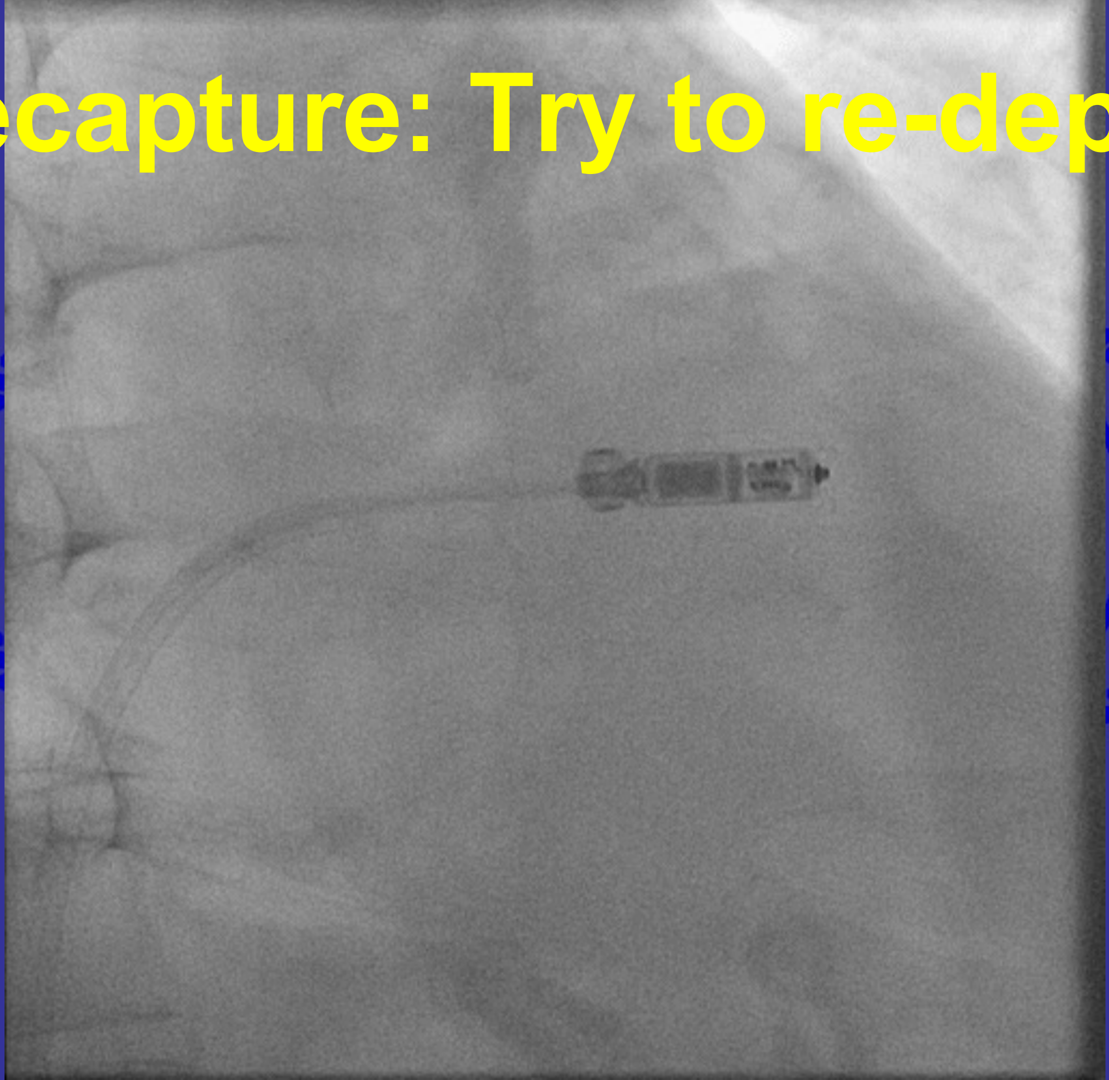

The Delivery Sheath Re-align with Micra for re-capture

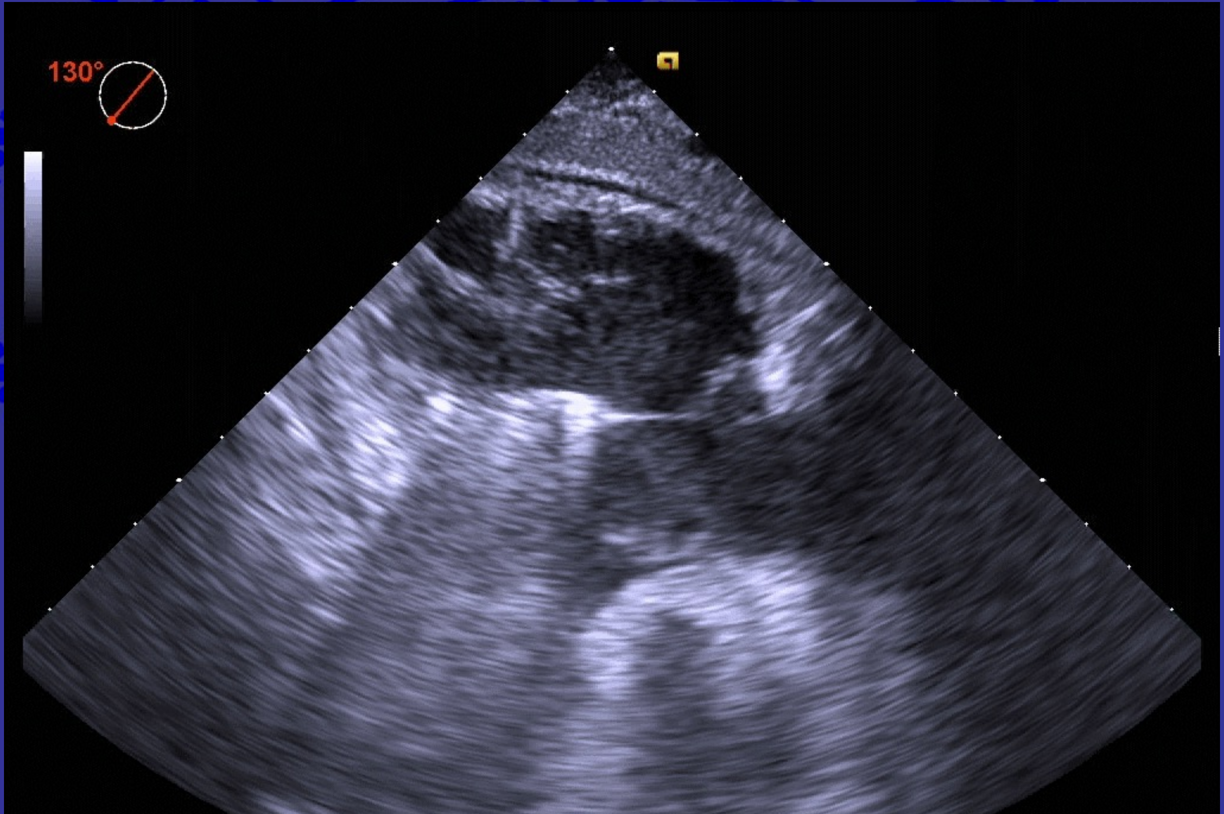

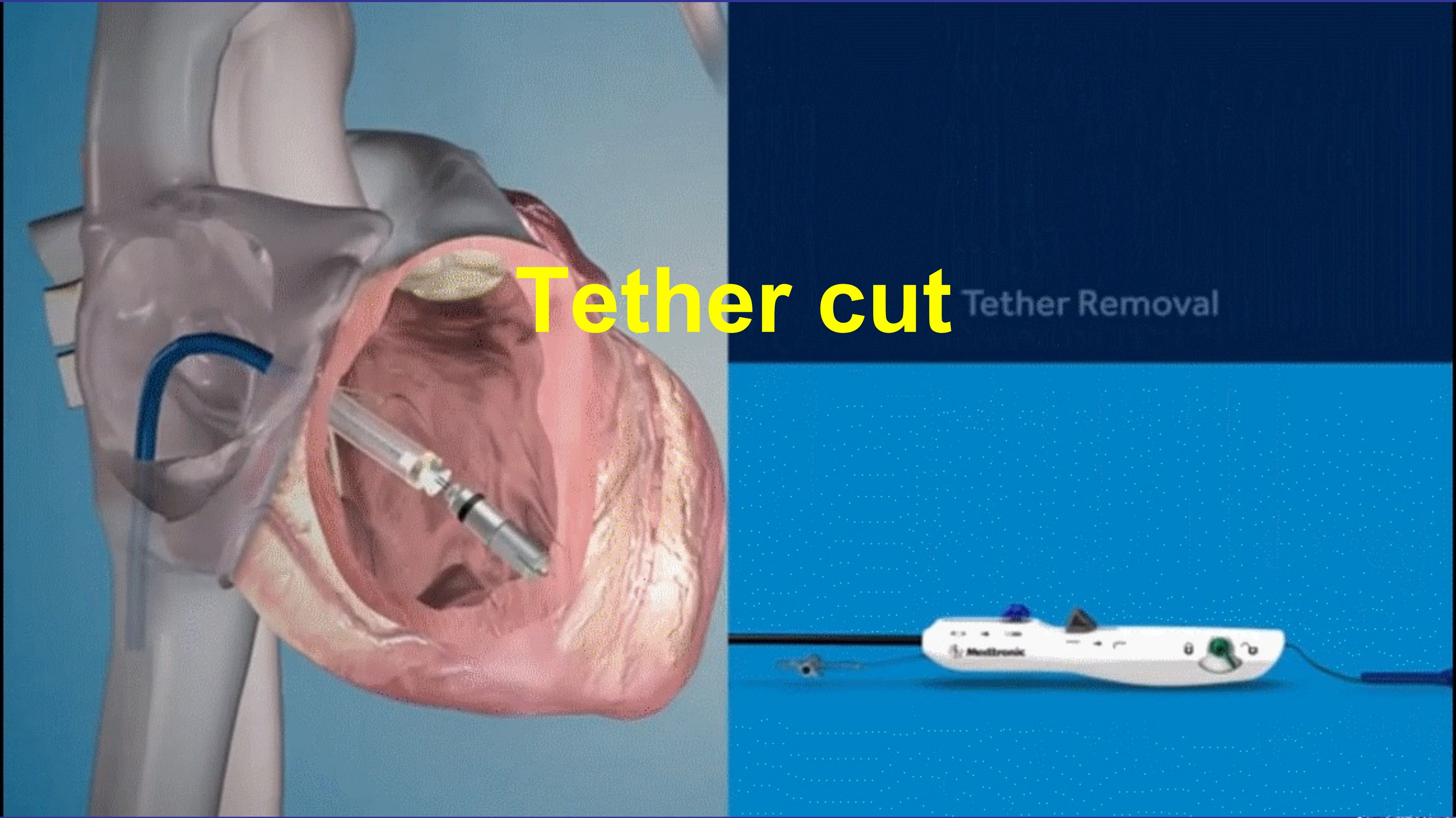
Tether cut
Tether Removal

Micra Tether Removal

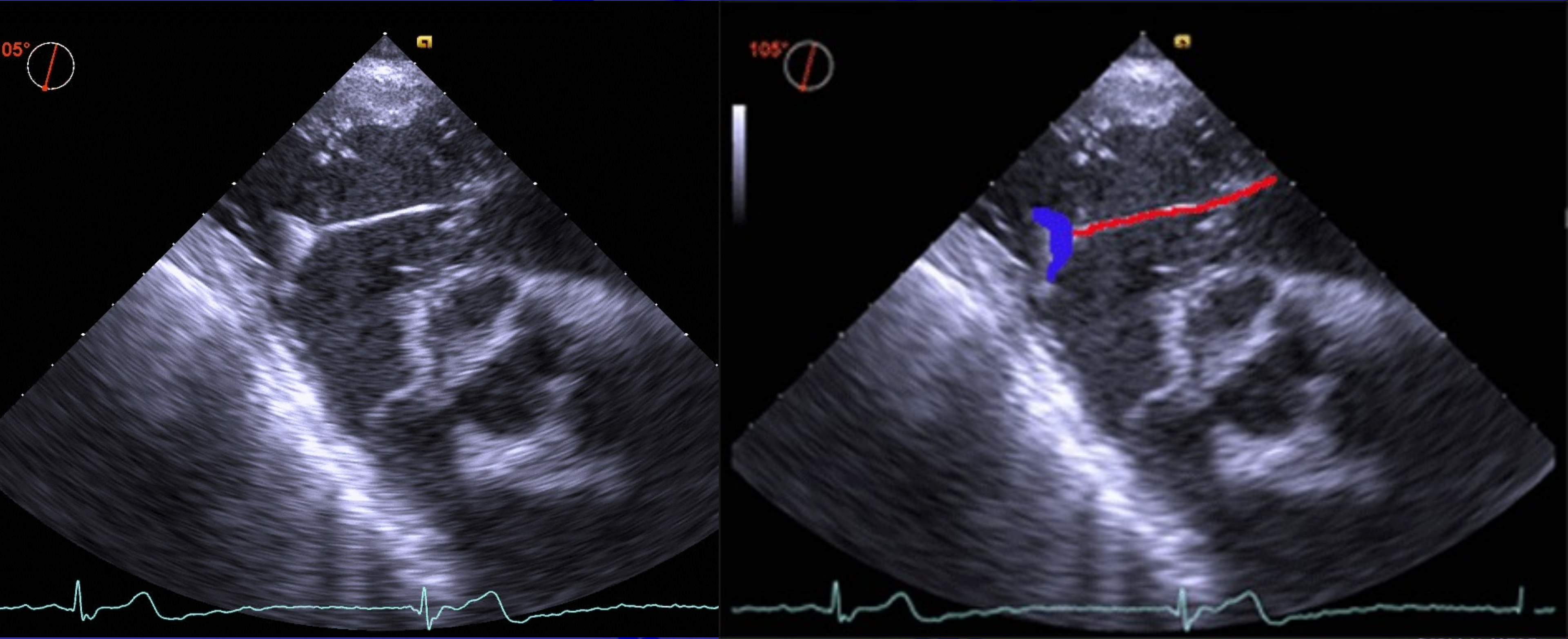

Micra Tether Removed

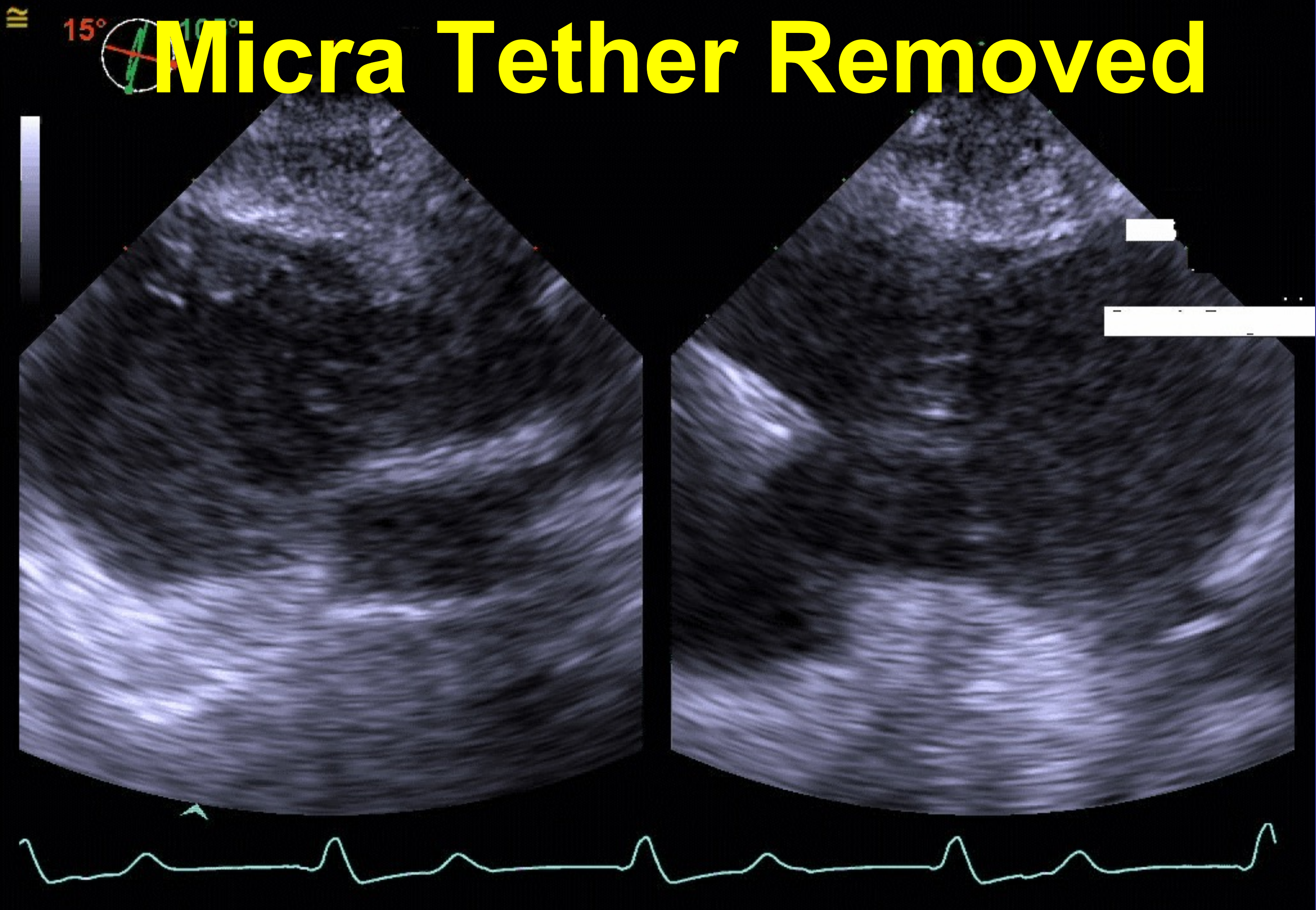

Pull the delivery system back into the Sheath

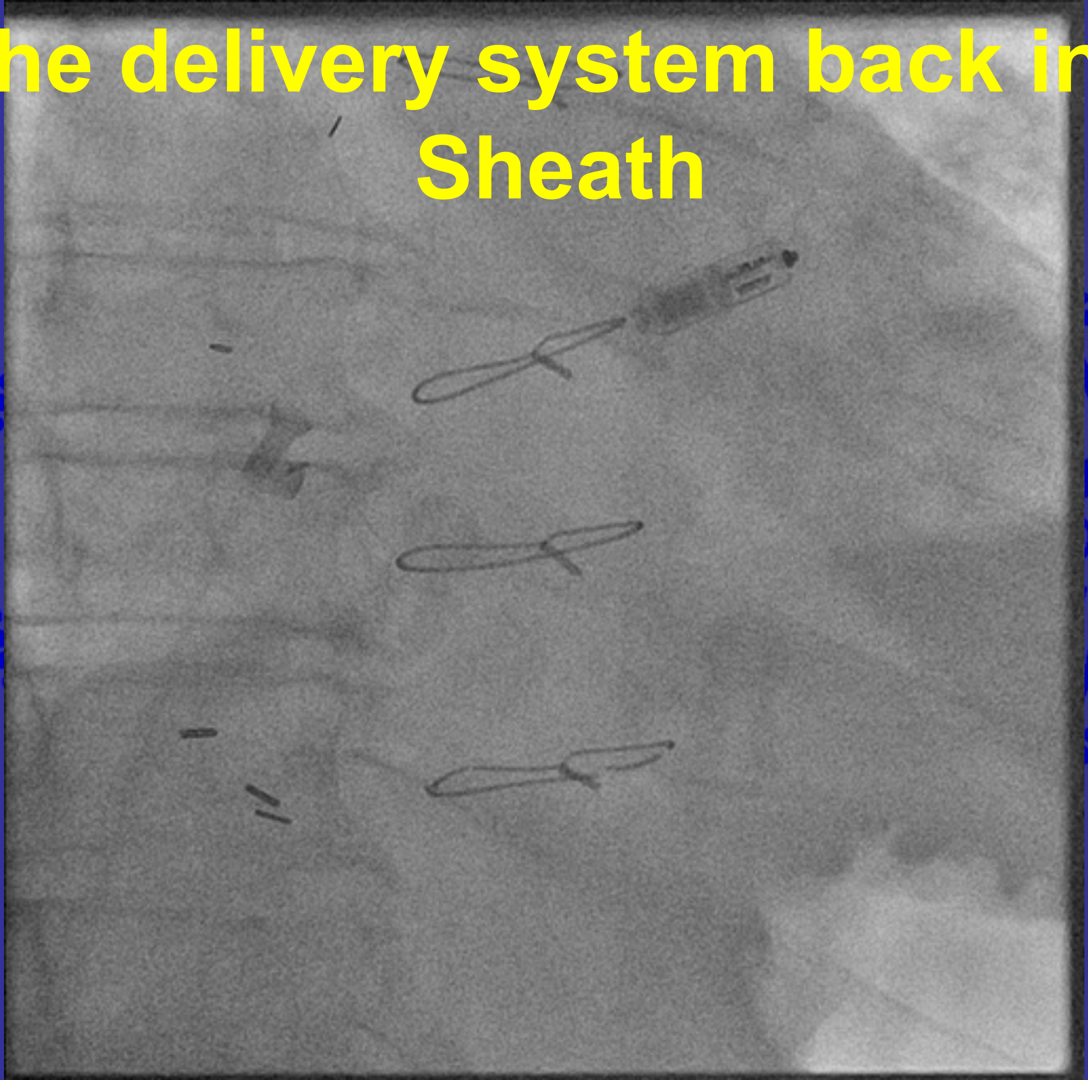

Final Micra Position

Post-Implant

- Monitor and Observe Micra Position Post-Final Implant
  - Trans-gastric view (long and short axis of RV) with optimal view of long axis with visualization of pulmonary valve, device, and tricuspid valve all in same view. Use biplane to assist. In 2d (not biplane), LP-TV and LP-PV measurements made at end-diastole.
  - Mid-esophageal Short Axis View: Optimal view of tricuspid valve, device, and pulmonary valve all in same view. Biplane can additionally assist to optimize image.
  - Mid-esophageal 4-Chamber View (RV focused): Visualize RV, septum, device, and tricuspid valve.
  - Rule out Effusion: Standard mid-esophageal 4-Chamber View.

Gastric View

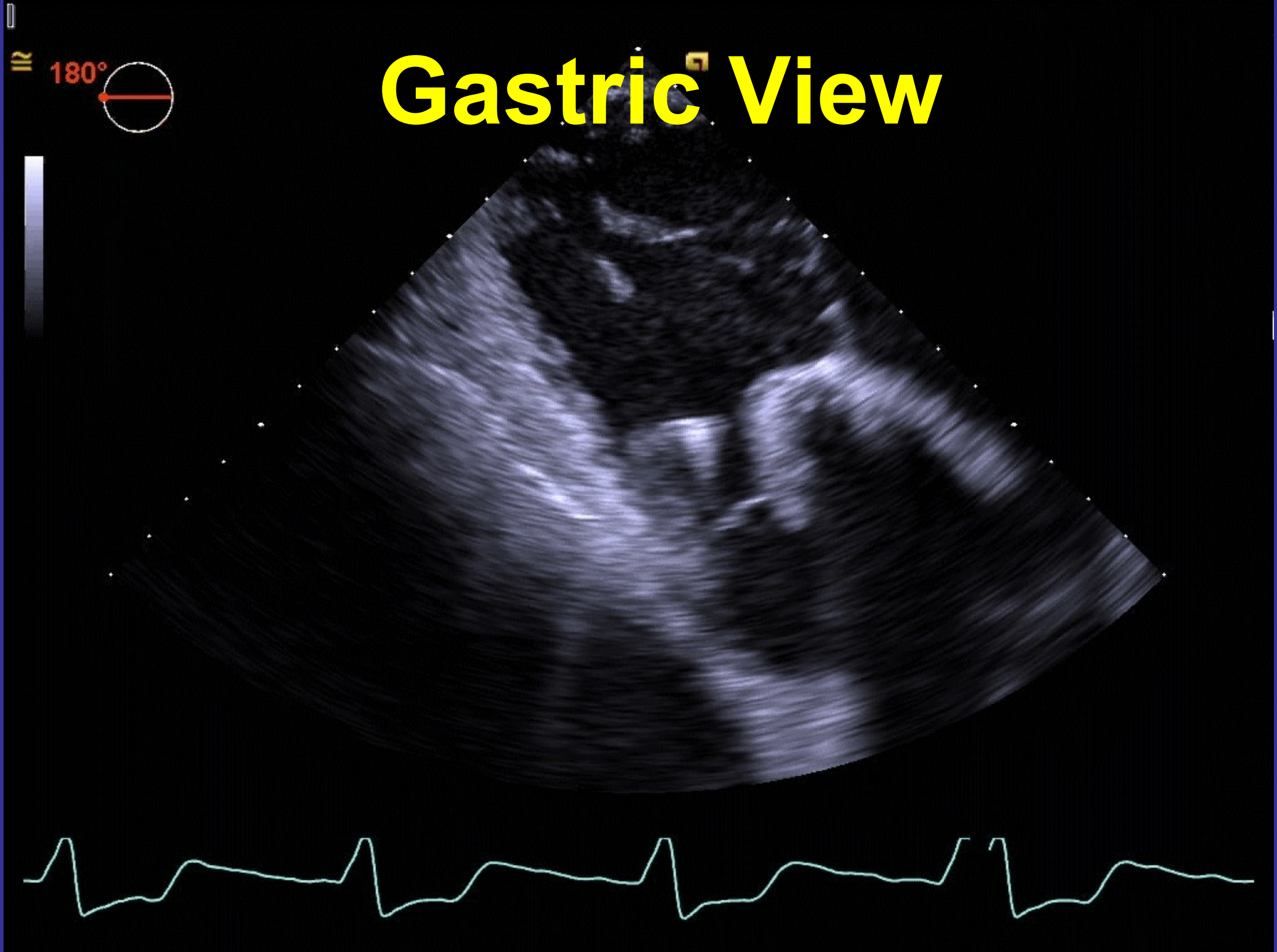

Gastric View

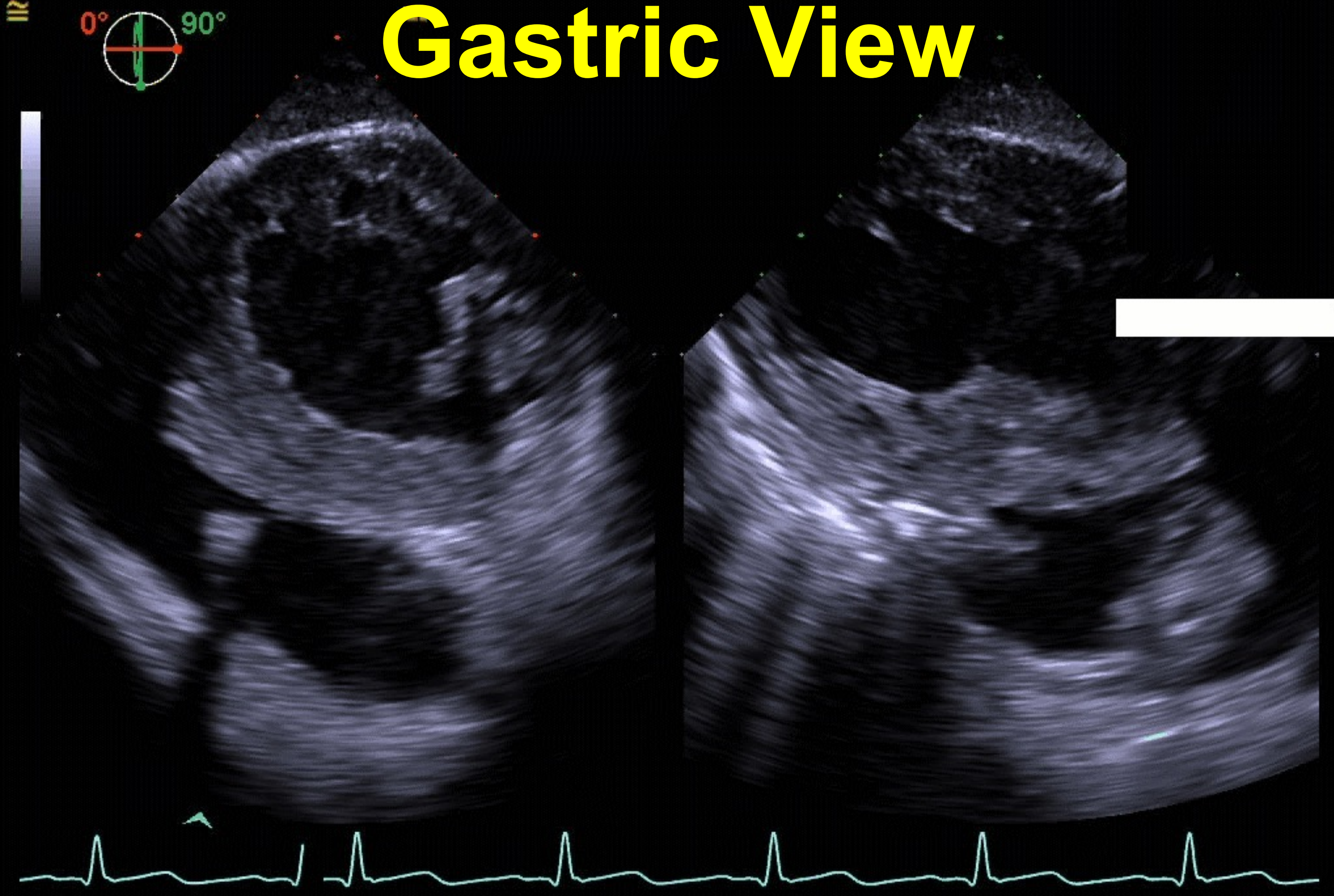

Gastric View Biplane

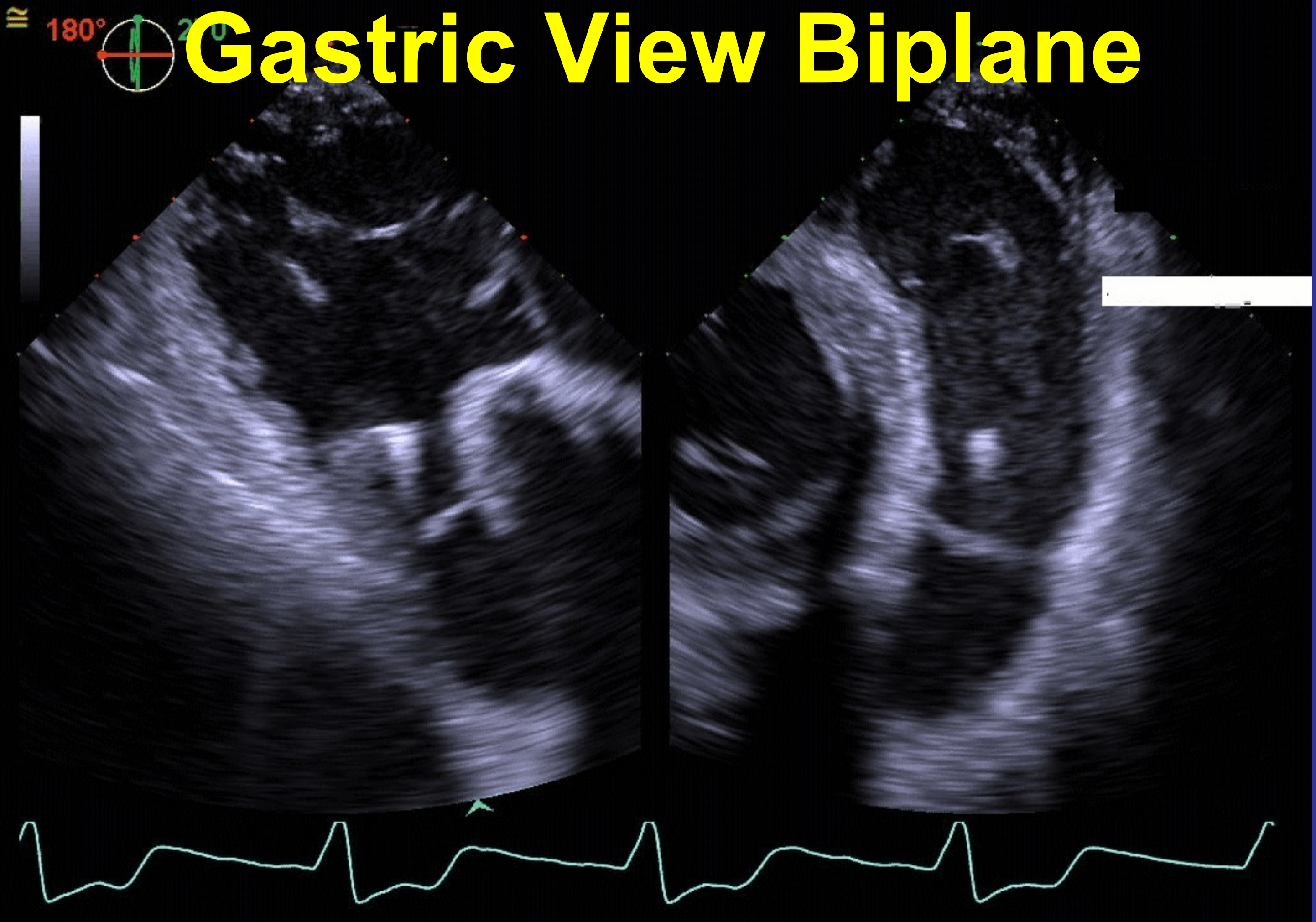

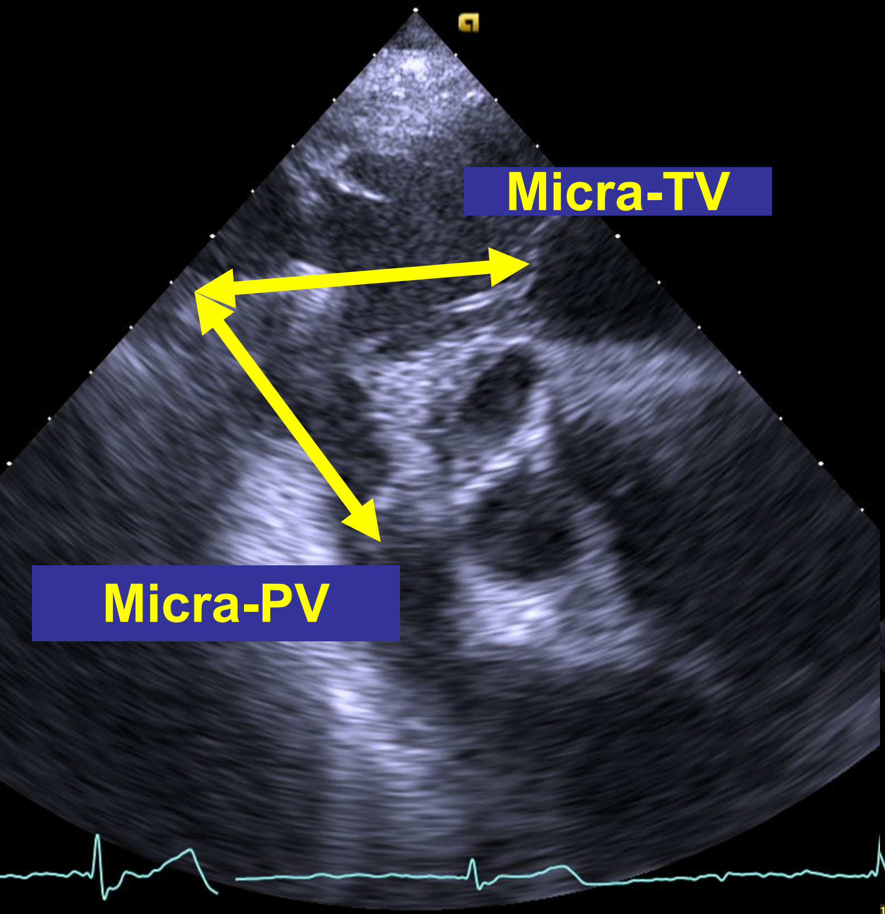
Micra-TV
Micra-PV

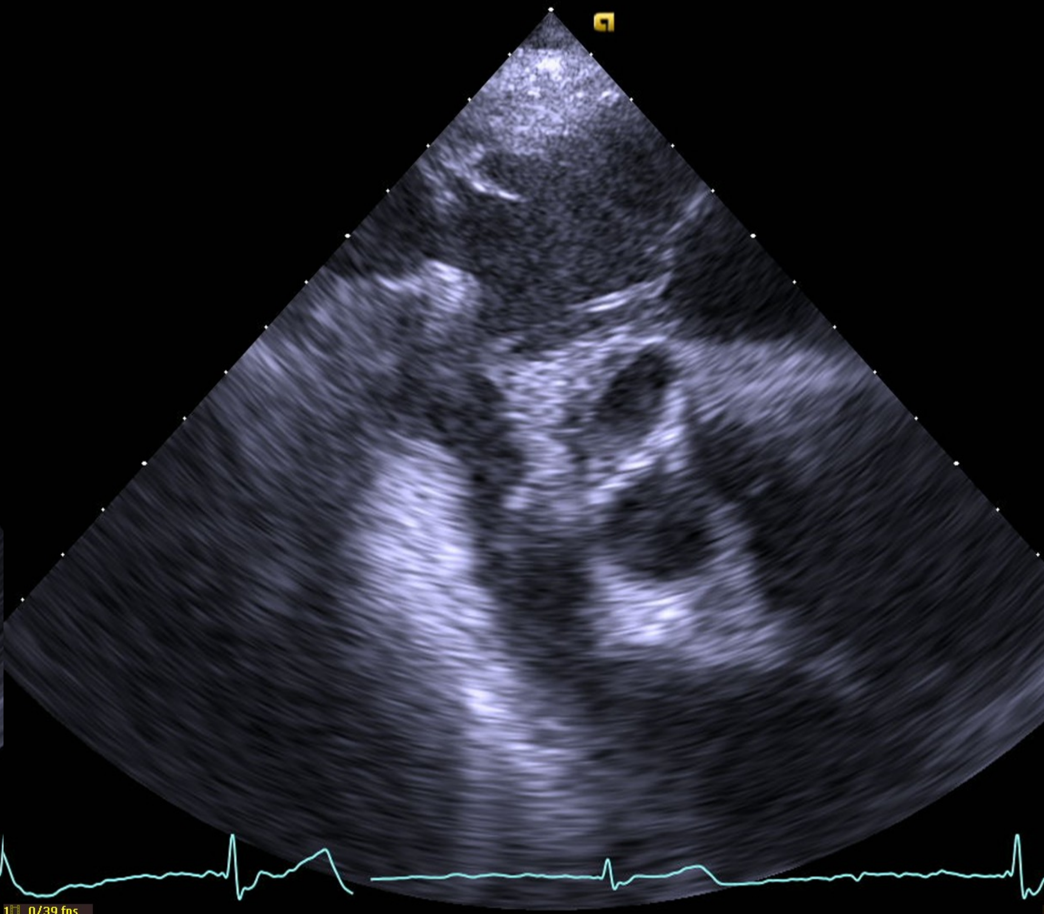

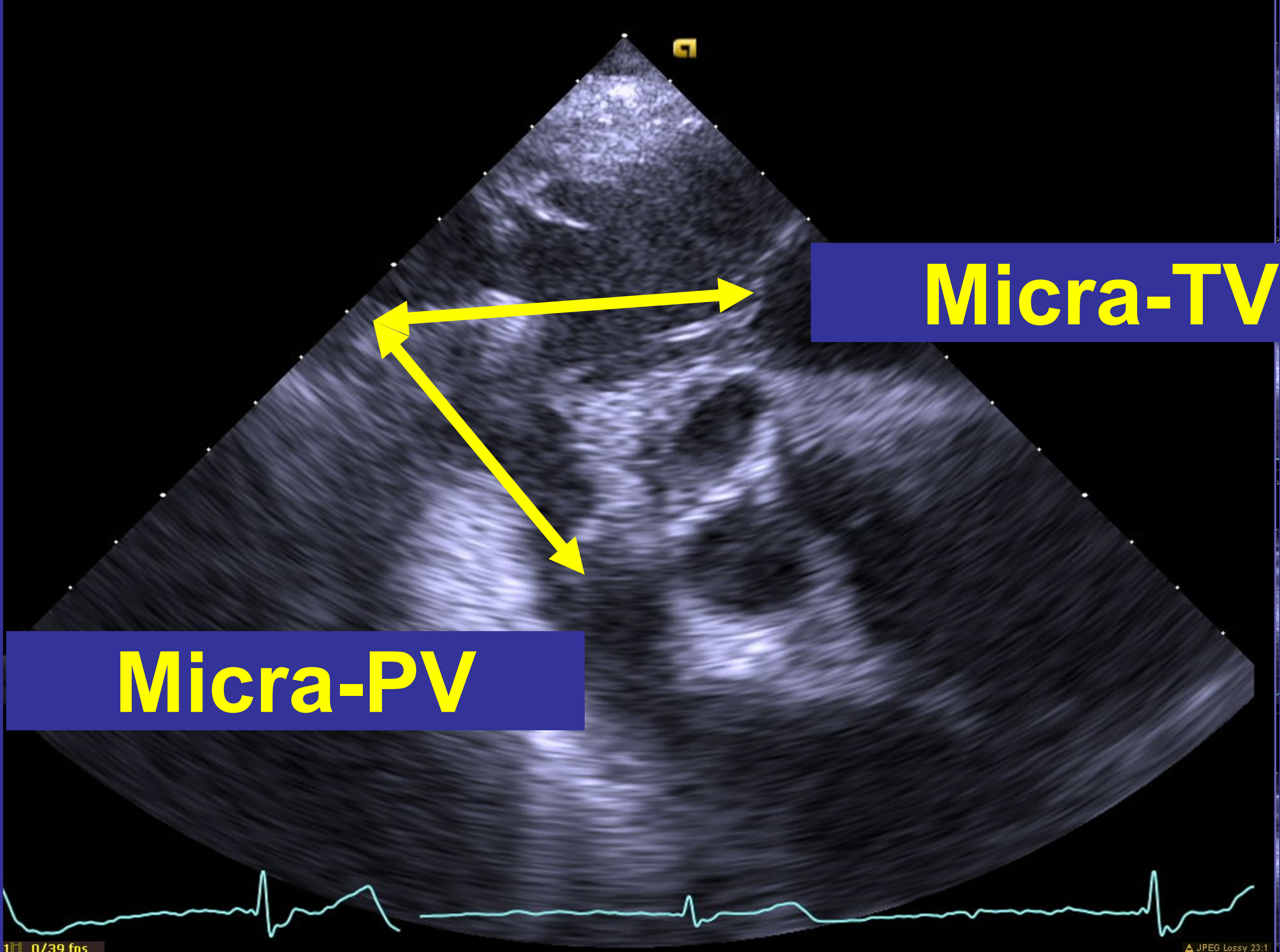
Micra-TV
Micra-PV

Mid Esophageal SAX View

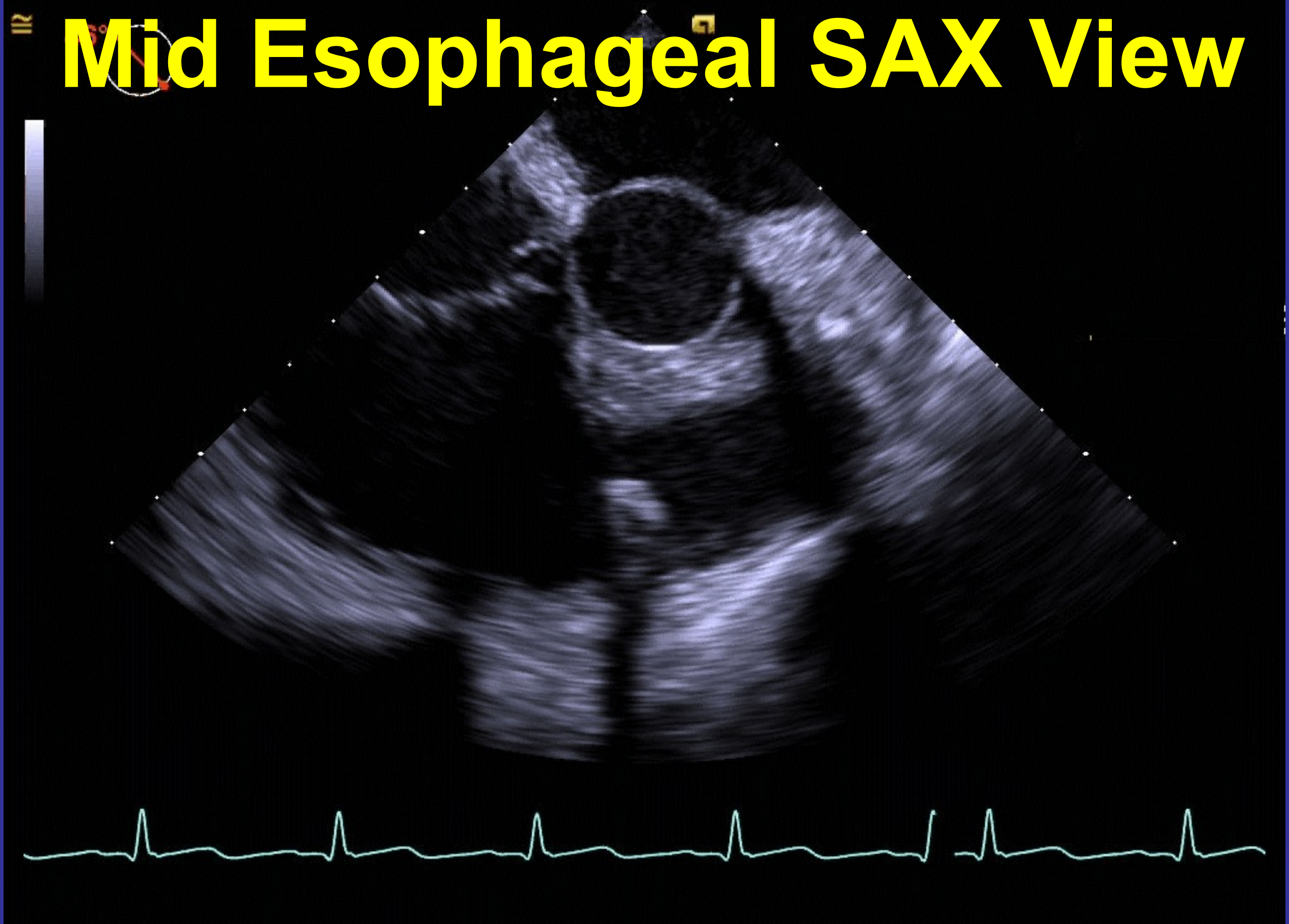

Mid Esophageal SAX View Biplane

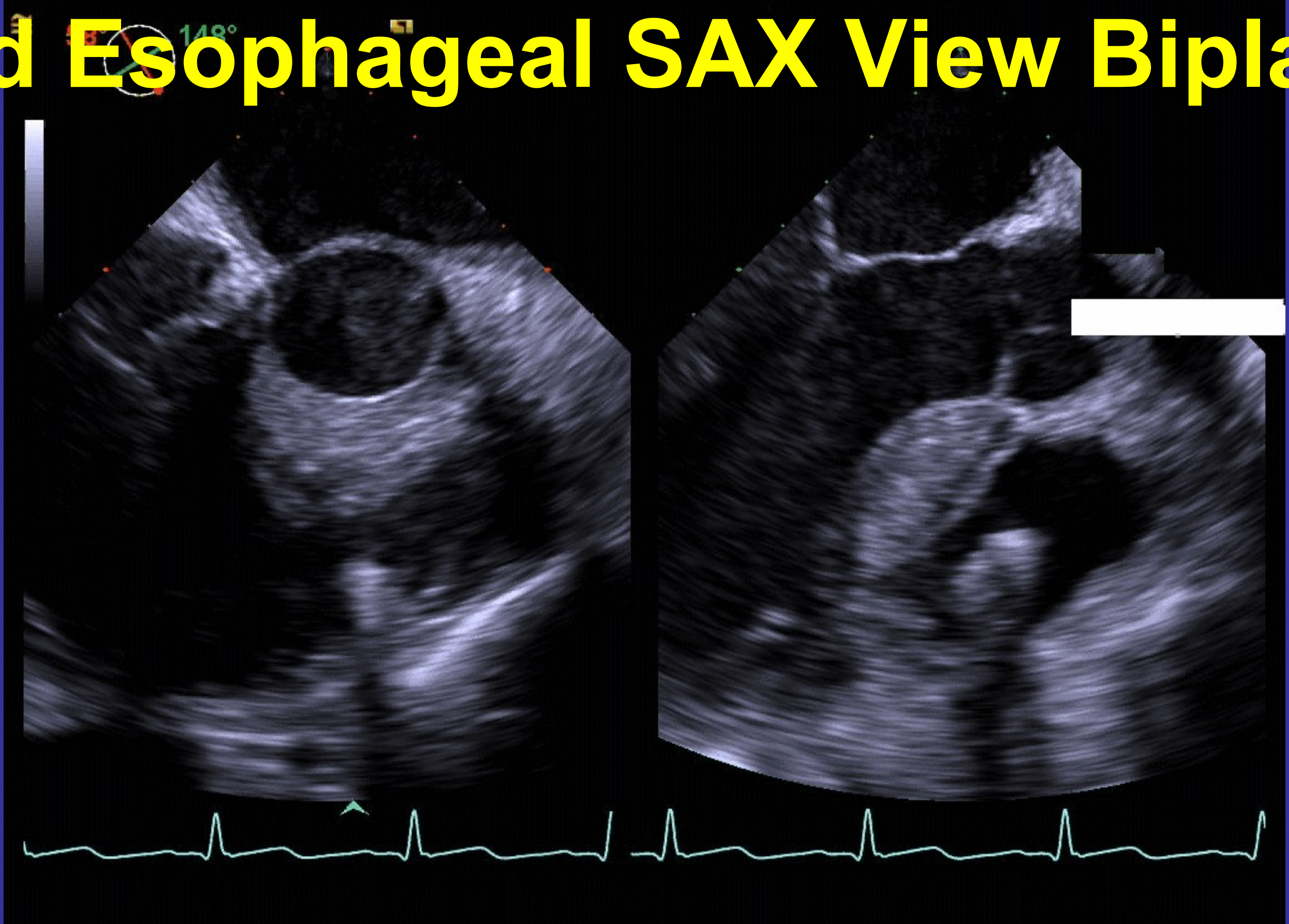

Mid Esophageal View 4 Chamber

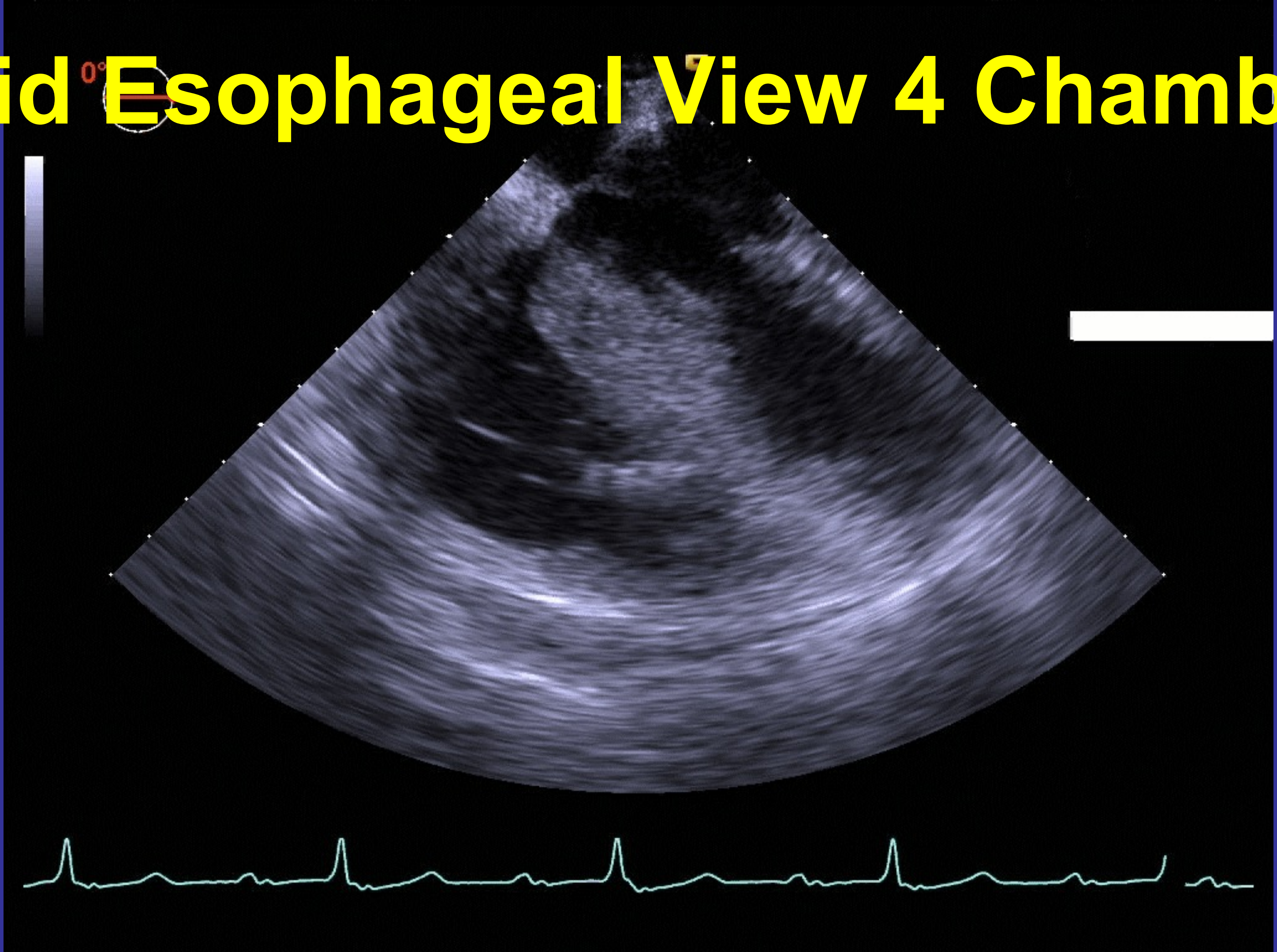

Post Micra: no pericardial Effusion

Post: no effusion

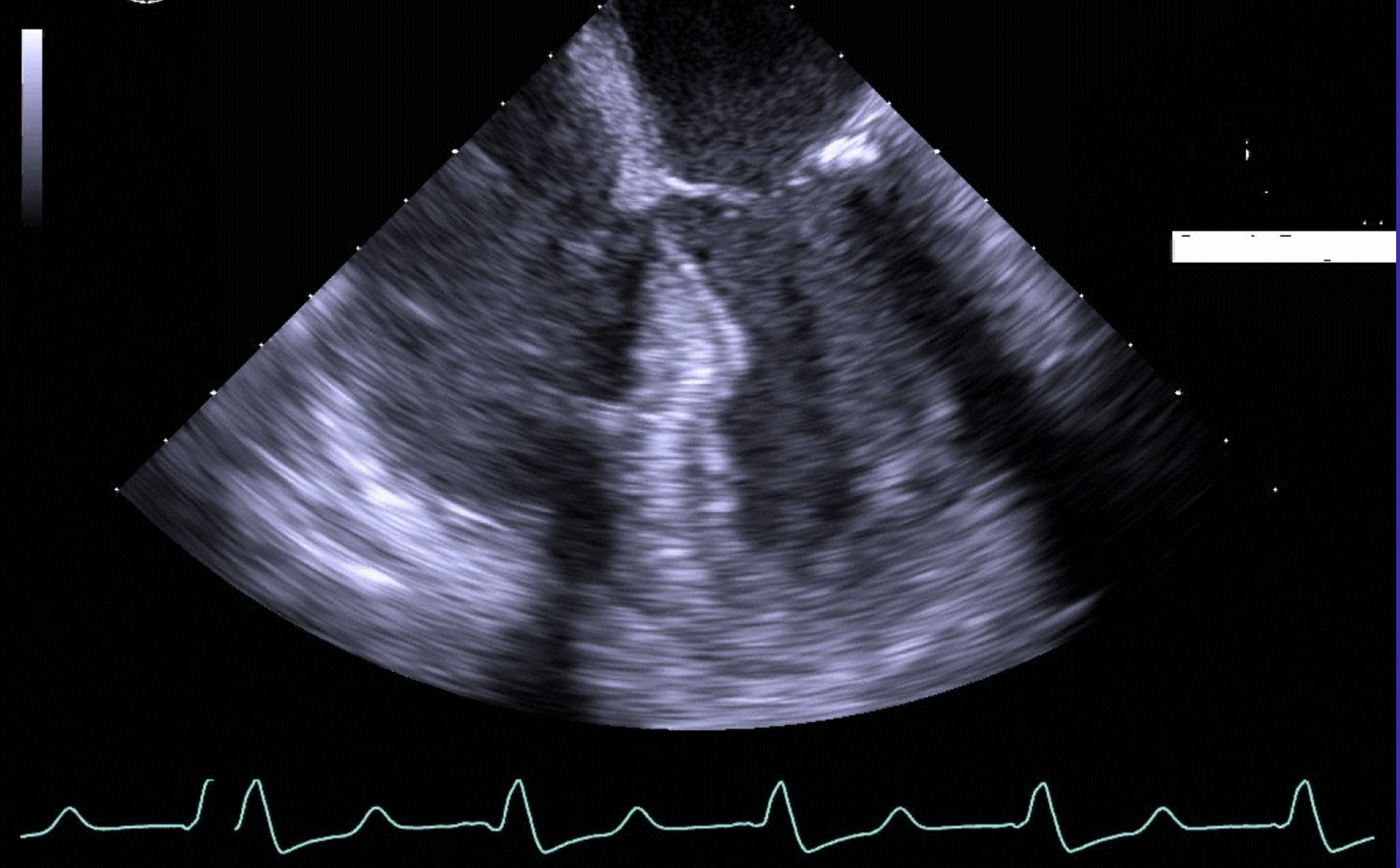

In Review

- Guidewire/Sheath Deployment
- The Delivery System
- Pre-Delivery: Anchor Location Optimization
- Deployment
- Tether (“Tug) Test/ Tether Removal
- Monitor and Measure Micra Position Post-Final Implant
